# Deconstructing the differences: a comparison of GBD 2010 and CHERG’s approach to estimating the mortality burden of diarrhea, pneumonia, and their etiologies

**DOI:** 10.1186/s12879-014-0728-4

**Published:** 2015-01-16

**Authors:** Stephanie D Kovacs, Kim Mullholland, Julia Bosch, Harry Campbell, Mohammad H Forouzanfar, Ibrahim Khalil, Stephen Lim, Li Liu, Stephen N Maley, Colin D Mathers, Alastair Matheson, Ali H Mokdad, Kate O’Brien, Umesh Parashar, Torin T Schaafsma, Duncan Steele, Stephen E Hawes, John T Grove

**Affiliations:** Strategic Analysis, Research and Training Program, University of Washington, Seattle, WA USA; London School of Hygiene & Tropical Medicine, Melbourne, Australia; Murdoch Childrens Research Institute, Melbourne, Australia; Menzies School of Health Research, Charles Darwin University, Darwin, Australia; Bill & Melinda Gates Foundation, Seattle, WA USA; University of Edinburgh, Edinburgh, Scotland; Institute for Health Metrics and Evaluation, University of Washington, Seattle, WA USA; Institute of International Programs, Johns Hopkins Bloomberg School of Public Health, Baltimore, MD USA; Department of Health Statistics and Informatics, World Health Organization, Geneva, Switzerland; Department of International Health, Johns Hopkins Bloomberg School of Public Health, Baltimore, MD USA; Division of Viral Diseases, Centers for Disease Control and Prevention, Atlanta, GA USA

## Abstract

**Background:**

Pneumonia and diarrhea are leading causes of death for children under five (U5). It is challenging to estimate the total number of deaths and cause-specific mortality fractions. Two major efforts, one led by the Institute for Health Metrics and Evaluation (IHME) and the other led by the World Health Organization (WHO)/Child Health Epidemiology Reference Group (CHERG) created estimates for the burden of disease due to these two syndromes, yet their estimates differed greatly for 2010.

**Methods:**

This paper discusses three main drivers of the differences: data sources, data processing, and covariates used for modelling. The paper discusses differences in the model assumptions for etiology-specific estimates and presents recommendations for improving future models.

**Results:**

IHME’s Global Burden of Disease (GBD) 2010 study estimated 6.8 million U5 deaths compared to 7.6 million U5 deaths from CHERG. The proportional differences between the pneumonia and diarrhea burden estimates from the two groups are much larger; GBD 2010 estimated 0.847 million and CHERG estimated 1.396 million due to pneumonia. Compared to CHERG, GBD 2010 used broader inclusion criteria for verbal autopsy and vital registration data. GBD 2010 and CHERG used different data processing procedures and therefore attributed the causes of neonatal death differently. The major difference in pneumonia etiologies modeling approach was the inclusion of observational study data; GBD 2010 included observational studies. CHERG relied on vaccine efficacy studies.

**Discussion:**

Greater transparency in modeling methods and more timely access to data sources are needed. In October 2013, the Bill & Melinda Gates Foundation (BMGF) hosted an expert meeting to examine possible approaches for better estimation. The group recommended examining the impact of data by systematically excluding sources in their models. GBD 2.0 will use a counterfactual approach for estimating mortality from pathogens due to specific etiologies to overcome bias of the methods used in GBD 2010 going forward.

**Electronic supplementary material:**

The online version of this article (doi:10.1186/s12879-014-0728-4) contains supplementary material, which is available to authorized users.

## Background

Historically, pneumonia and diarrheal disease are the leading causes of death for children under five (U5) worldwide after neonatal causes [1-3]. However, accurate quantification of cause-specific mortality fractions for pneumonia and diarrheal diseases is difficult to achieve; current estimates vary substantially across modeling groups. Differences in etiology-specific mortality estimates are of considerable concern as global and national priorities for interventions including vaccination campaigns, routine immunizations systems, and allocation of health resources are informed by these estimates. The introduction of the United Nations (UN) Millennium Development Goals (MDGs) turned additional attention to the need for improved burden of disease estimates to track global and individual countries’ progress toward achievements in the reduction of child deaths [4]. These factors have led to efforts in mathematical disease burden modeling to provide estimates of cause-specific disease morbidity and mortality. Two major efforts have been undertaken to address the shortcomings in this area, both building on previous attempts at comprehensive estimates of disease burden: 1) the Global Burden of Disease (GBD) 2010 study led by the Institute for Health Metrics and Evaluation (IHME), and 2) the global pediatric disease burden modeling work by the WHO and UNICEF/Child Health Epidemiology Reference Group (CHERG).

The GBD 2010 study is led by IHME at the University of Washington, Seattle, in partnership with the Imperial College London, the University of Queensland, Harvard University, and many other institutions, and its estimates include total and cause-specific mortality for children U5. IHME estimated the global and country-level U5 mortality from all causes, including acute respiratory infections (ARI) that they further divided into lower and upper respiratory infection (LRI and URI, respectively) and diarrhea. Selected etiology-specific estimates of LRI and diarrhea were also made at the country and global levels. Only the global estimates are currently in the public domain [5,6].

CHERG was established by WHO and UNICEF in 2001, but is independent of the various WHO program areas. CHERG has undertaken comprehensive disease burden modeling of causes of death in children U5 and has provided sequential annual estimates from 2001 through 2011. During each revision, the CHERG model makes improvements and includes newly available data. In 2012, CHERG published year 2010 causes of death, which built upon previously models published in 2010. In 2013, CHERG published year 2011 causes of death, as well as estimates for selected etiologies of pneumonia and diarrhea [7-10].

In this paper we present an overview of the magnitude of the differences between GBD 2010 and CHERG estimates of U5 mortality related to pneumonia and diarrhea, followed by a discussion of three broad areas that may drive the variation in these estimates: data sources, data processing, and the use of risk factor data (covariates). Next, we present conceptual models for the two groups’ pneumonia and diarrhea etiology models and discuss differences in the modeling assumptions. Finally, we conclude with recommendations from an expert meeting, hosted by the Bill & Melinda Gates Foundation (BMGF) in October 2013, for improving the estimates for the upcoming CHERG and GBD 2.0 publications.

### Magnitude of the differences in U5 mortality

Both the GBD 2010 and the CHERG cause-specific mortality estimation methods use total U5 mortality as the denominator, but their sources for this denominator differ, which has great influence on their final cause-specific estimates. Tables 1, 2 and 3 show GBD 2010 and CHERG estimates for year 2010 total U5 mortality due to pneumonia, diarrhea, and their etiologies. CHERG used the Interagency Group for Child Mortality Estimation (IGME) year 2010 U5 mortality estimate of 7.660 million deaths [11] while GBD 2010 provided its own estimate of 6.880 million deaths, a difference of nearly 760,000 deaths (11%).

**Table 1:** **Estimates of the global burden due to pneumonia and diarrhea in children under five in 2010** [6,7]

	**CHERG**		**GBD 2010**		
	**Deaths (×1,000)**	**% total U5 mortality**	**Deaths (×1,000)**	**% total U5 mortality**	% difference*
Total U5 mortality	7,622	100%	6,841	100%	11%
Pneumonia/LRI** U5	1,396	18.3%	847	12.4%	49%
Pneumonia/LRI** neonatal (0–27 days)	325	4.3%	194	2.8%	51%
Pneumonia/LRI** postneonatal	1,071	14.1%	654	9.6%	48%
Diarrhea U5	801	10.5%	666	9.7%	18%
Diarrhea neonatal (0–27 days)	50	0.7%	77	1.1%	−43%
Diarrhea postneonatal	751	9.9%	589	8.6%	24%

*Percent difference calculated: (CHERG - GBD 2010)/((CHERG + GBD 2010)/2) × 100.

**LRI: Lower respiratory infection.

**Table 2:** **Estimates of the global burden due to pneumonia etiologies in children under five in 2010/2011** [6,9]

	**CHERG (2011)**		**GBD 2010**	
**Pneumonia/LRI etiology**	**Deaths (×1,000)**	**% U5 pneumonia mortality**	**Deaths (×1,000)**	**% U5 LRI mortality**
All	1,257	100.0%	847.1	100.0%
Pneumococcal pneumonia	412	32.7%	168	19.8%
*H. influenzae* type B	197	15.7%	184	21.7%
Respiratory syncytial virus	-	-	234	27.6%
Influenza	-	*-*	119	14.0%
Other lower respiratory infections	-	-	143	16.9%

**Table 3:** **Estimates of the global burden due to diarrhea etiologies in children under five in 2010/2011** [6,10]

	**CHERG (2011)**		**GBD 2010**	
**Diarrhea etiology**	**Deaths (×1,000)**	**% U5 diarrhea mortality**	**Deaths (×1,000)**	**% U5 diarrhea mortality**
Total	712*	100%	666*	100%
Cholera**	9	1.3%	42.5	6.4%
Other salmonella infections			29	4.4%
Shigellosis	28	3.9%	43.6	6.5%
Enteropathogenic *E. coli* infection	79	11.1%	72.8	10.9%
Enterotoxigenic *E. coli* infection	42	6.0%	38.7	5.8%
Campylobacter enteritis	22	3.2%	63.6	9.5%
Amoebiasis			9.2	1.4%
Cryptosporidiosis	14	2.0%	83	12.5%
Rotaviral enteritis	197	27.8%	173	26.0%
Other diarrheal diseases			109	16.4%
Calicivirus	71	9.9%		
Astrovirus	15	2.1%		
Adenovirus	22	3.1%		
All Salmonella	18	2.5%		
*Giardia lamblia*	16	2.3%		
*Entamoeba histolytica*	1	0.2%		
Unknown etiology	176	24.5%		

*CHERG death estimates sum up to 710,000 and those of GBD2010 to 664,000 due to rounding adjustments of individual figures.

**CHERG cholera estimate only represents Vibrio Cholera O1.

Alkema and You examined possible factors that might account for differences in the overall U5 mortality rate produced in 2011 by IHME and IGME, assessing data sources, inclusion and exclusion criteria, outlier adjustment, model fitting, and covariate adjustment [12]. They determined that the data used in the model were responsible for larger differences than the model structures themselves. The authors concluded that if the two groups used the same data set in their separate models, they would have generated very similar estimates [12].

**Figure 1:**
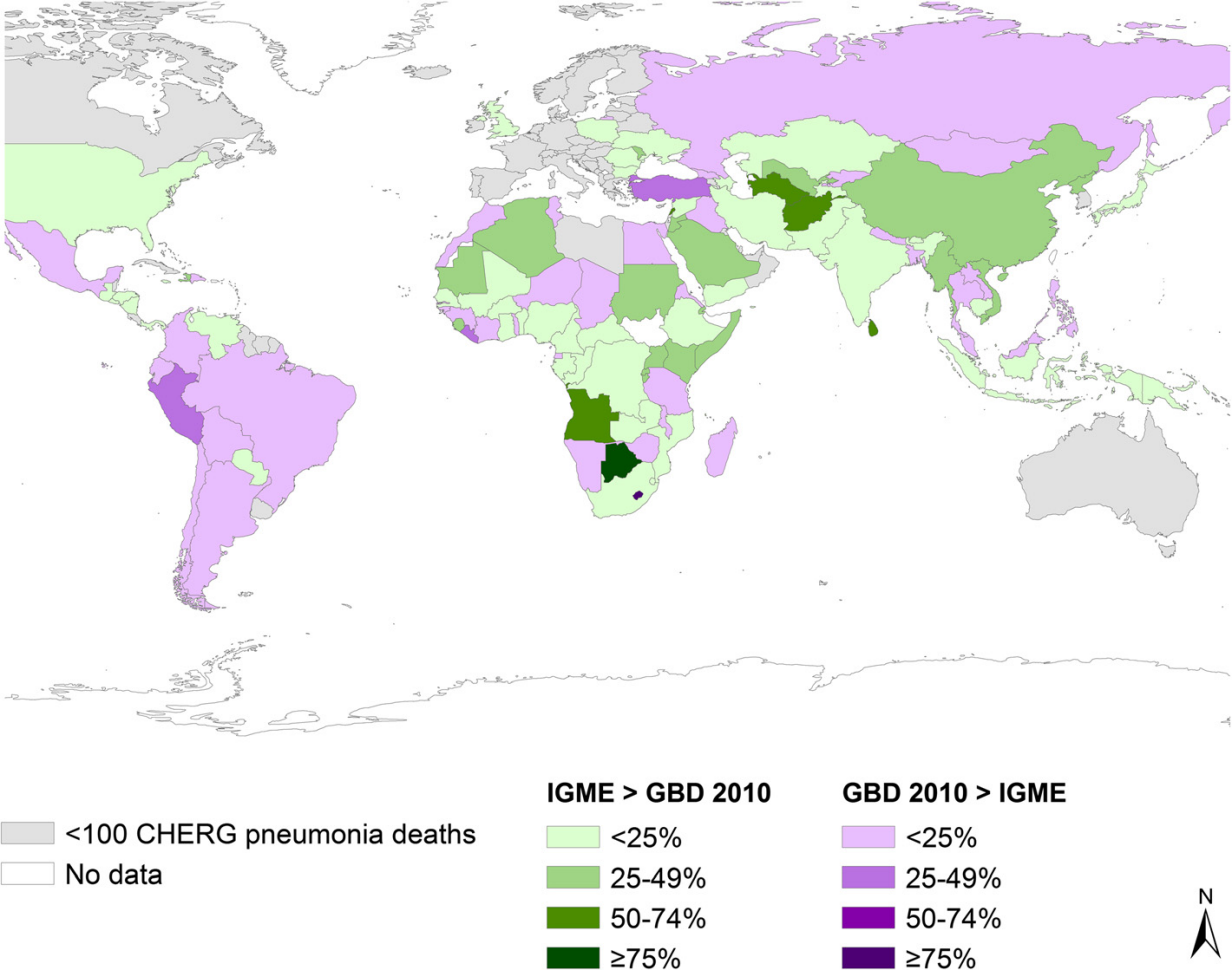
**Percent difference in IGME vs. GBD 2010 estimated U5 deaths, 2010.**

**Figure 2:**
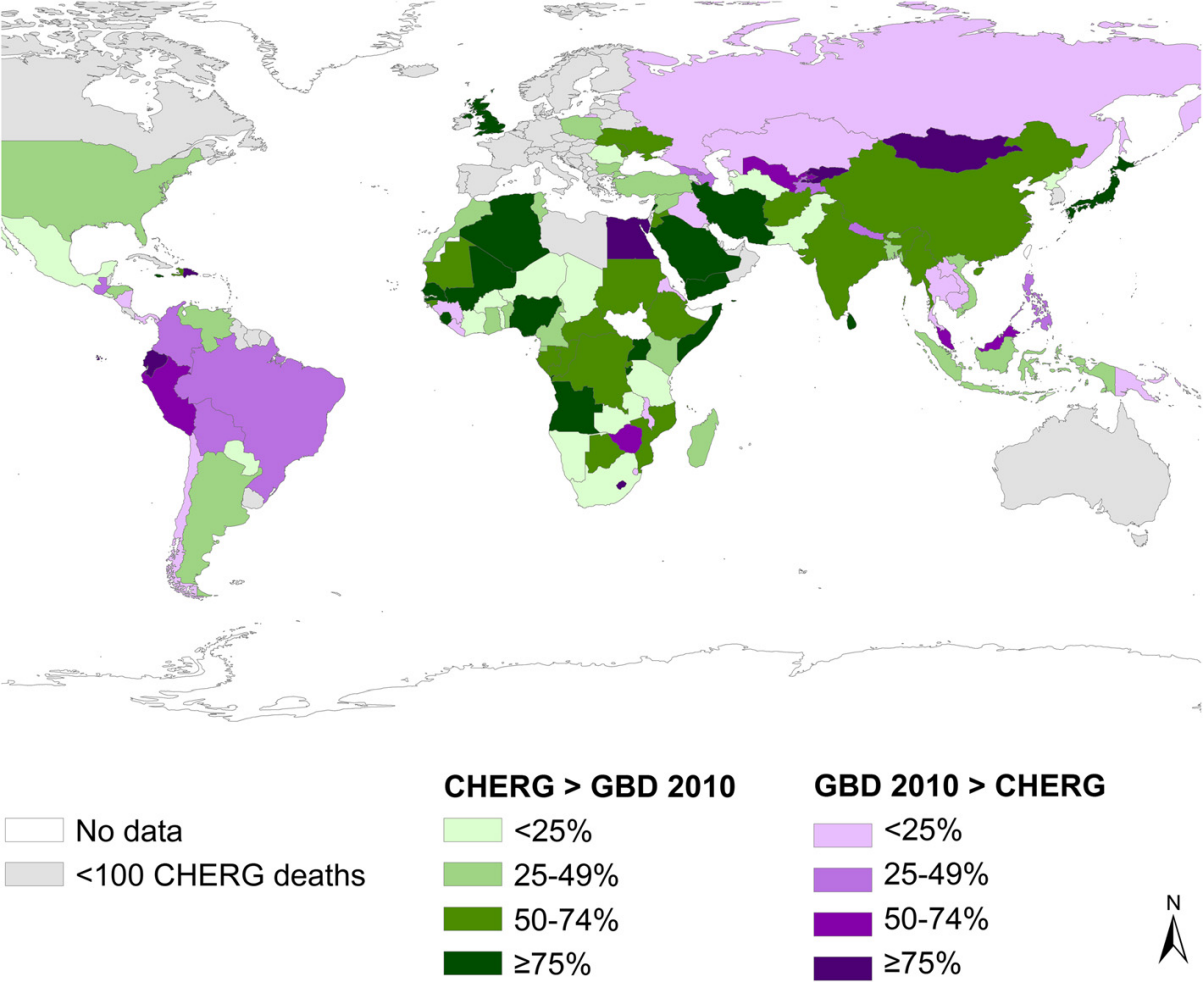
**Percent difference in CHERG vs. GBD 2010 estimated U5 pneumonia deaths, 2010.**

**Figure 3:**
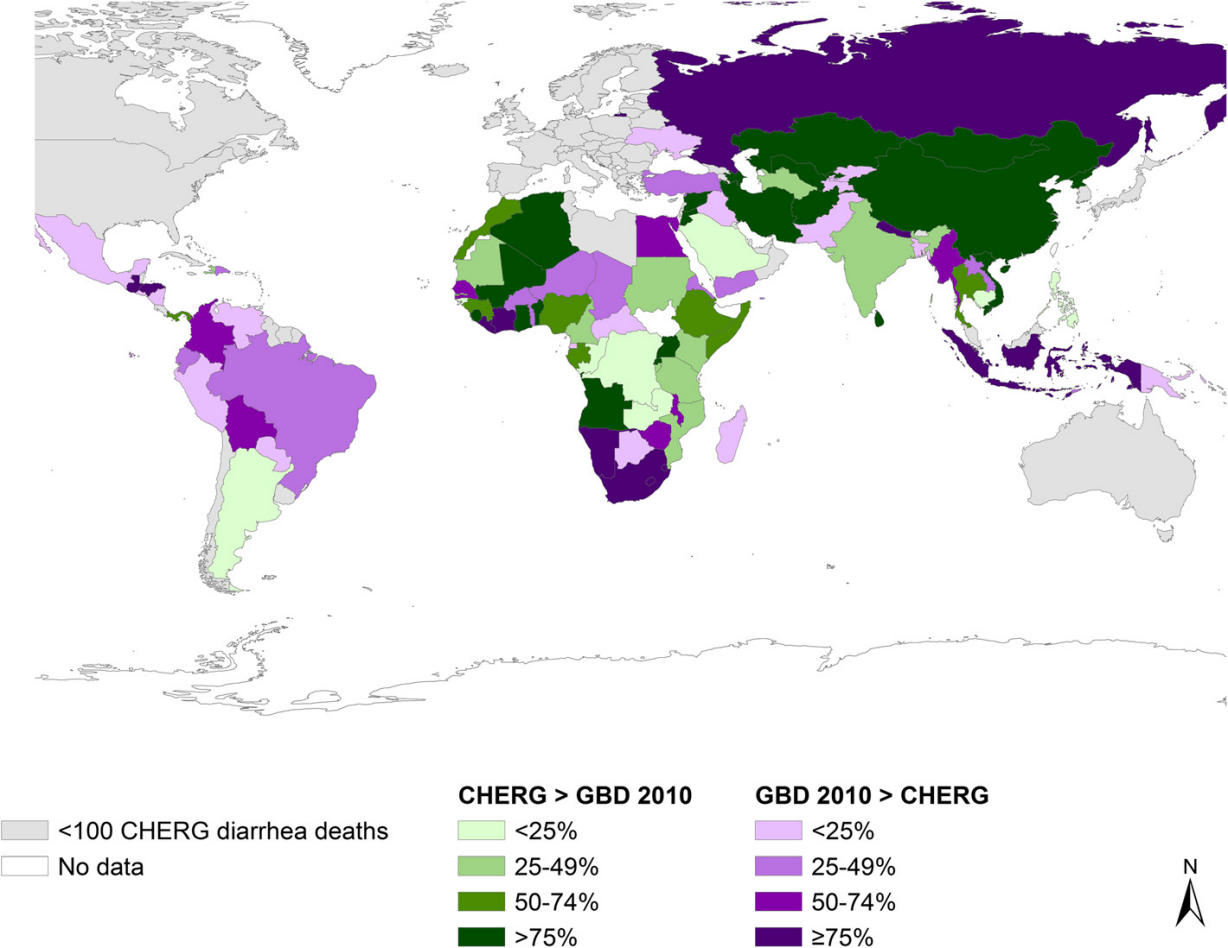
**Percent difference in CHERG vs. GBD 2010 estimated U5 diarrhea deaths, 2010.**

Differences in overall U5 mortality account for only some of the differences, as pneumonia and diarrhea cause-specific mortality fractions differ greatly between GBD 2010 and CHERG (Figures 1, 2 and 3, summarizing some of the data of Additional files 1, 2 and 3). The following sections describe drivers of the differences in the pneumonia and diarrhea mortality estimates.

### Drivers of the differences: data sources

Differences in data sources are a major driver of differences in the pneumonia and diarrhea mortality estimates. GBD 2010 and CHERG used different inclusion criteria for their models.

#### GBD 2010

**Figure 4:**
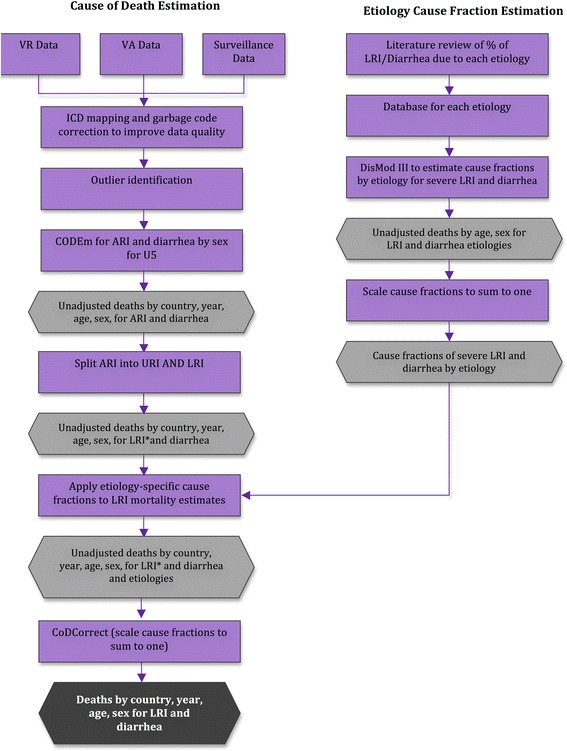
**GBD 2010 analytical approach for estimating the global mortality burden due to LRI, diarrhea and their etiologies.**

The GBD 2010 modeling process used the following steps for pneumonia and diarrhea mortality estimation: 1) data identification, 2) data processing, 3) Cause of Death Ensemble models (CODEm) for modeling acute respiratory infections (ARI) and diarrheal deaths, 4) proportional model for dividing ARI into lower respiratory infections (LRI) and upper respiratory infections (URI) deaths, 5) DisMod-MR model for estimating deaths due to LRI and diarrhea etiologies, and 6) CoDCorrect for scaling cause-specific mortality fractions to sum to one (Figure 4).

For ARI, GBD 2010 identified 2,725 data points of vital registration (VR) data, 100 verbal autopsy (VA) data points from multiple sites, and 17 data points of surveillance data. For diarrhea they used 2,696 data points of VR data and 264 data points from VA studies. A data point refers to the mortality rate for a specific cause in an individual country for a specific year. For example, if there are vital registration data for Tanzania from 1993–2003 available, this would account for 11 data points of VR data. Despite identifying many data points, some countries did not have any data available. In the CODEm models used to estimate ARI and diarrheal deaths [13], data were weighted based on their proximity to the country for which they estimated the cause-specific mortality fractions. In countries with data, the in-country data is weighted at 90%, data from the region is weighted at 9%, and data from the super-region is weighted at 1%. In countries with no data, 70% of the weight is given to data from the region and 30% of the weight is given to data from the super-region [13]. GBD 2010’s regions and super-regions group countries with similar epidemiology and economic development (e.g., high income countries were grouped together), in addition to geographic proximity. In this way, the model borrows data from neighboring countries in the region and super-region to fill in gaps where data are sparse.

GBD 2010 developed ARI and diarrhea cause of death models using all VR data, regardless of the level of registration completeness, and also developed models restricting the inclusion of VR data based on the level of registration ≥70%, ≥60%, ≥50%, ≥40%, and ≥30% completeness thresholds. All VA studies were considered for inclusion in GBD 2010 cause of death models for ARI and diarrhea. GBD 2010 researchers found very little difference in estimated U5 mortality due to ARI and diarrhea between the model including all VR data and the model that only included VR data that was at least 70% complete. The final model included all VR data that was at least 60% complete.

LRI deaths were further divided into five etiologies. LRI etiology models used data from existing systematic reviews and a database of published studies of ARI compiled by the WHO [14-16]. GBD 2010 included all studies from these reviews after applying additional exclusion criteria (details of the search strategy are available in Additional file 4, supplementary materials). An additional systematic search was conducted using the same inclusion and exclusion rules for studies published between 2006–2011, which identified an additional 54 studies for analysis (details of the search strategy are available in Additional file 4, supplementary materials) [6]. Forty-pneumonia deaths were due to pneumococcus andefficacy studies were used in the GBD 2010 LRI etiology models. (Additional file 2 lists all studies used to model pneumonia etiologies by both GBD 2010 and CHERG).

The GBD 2010 diarrhea etiology models included 189 studies from 1975–2010, with rotavirus-related data providing the largest number of studies at 126 (details of the search strategy are available in the supplementary materials and a comprehensive list of all studies used by both CHERG and GBD 2010 is available in Additional file 3) [6]. Similar to the CODEm models, the modeling procedure for LRI and diarrhea etiology models used hierarchal spatial effects by which country models were informed by data from the country, region, and super-region only. Additionally, the models were age integrated and therefore the models for U5 mortality were informed only by data using the same age ranges in the observational studies.

#### CHERG

**Figure 5:**
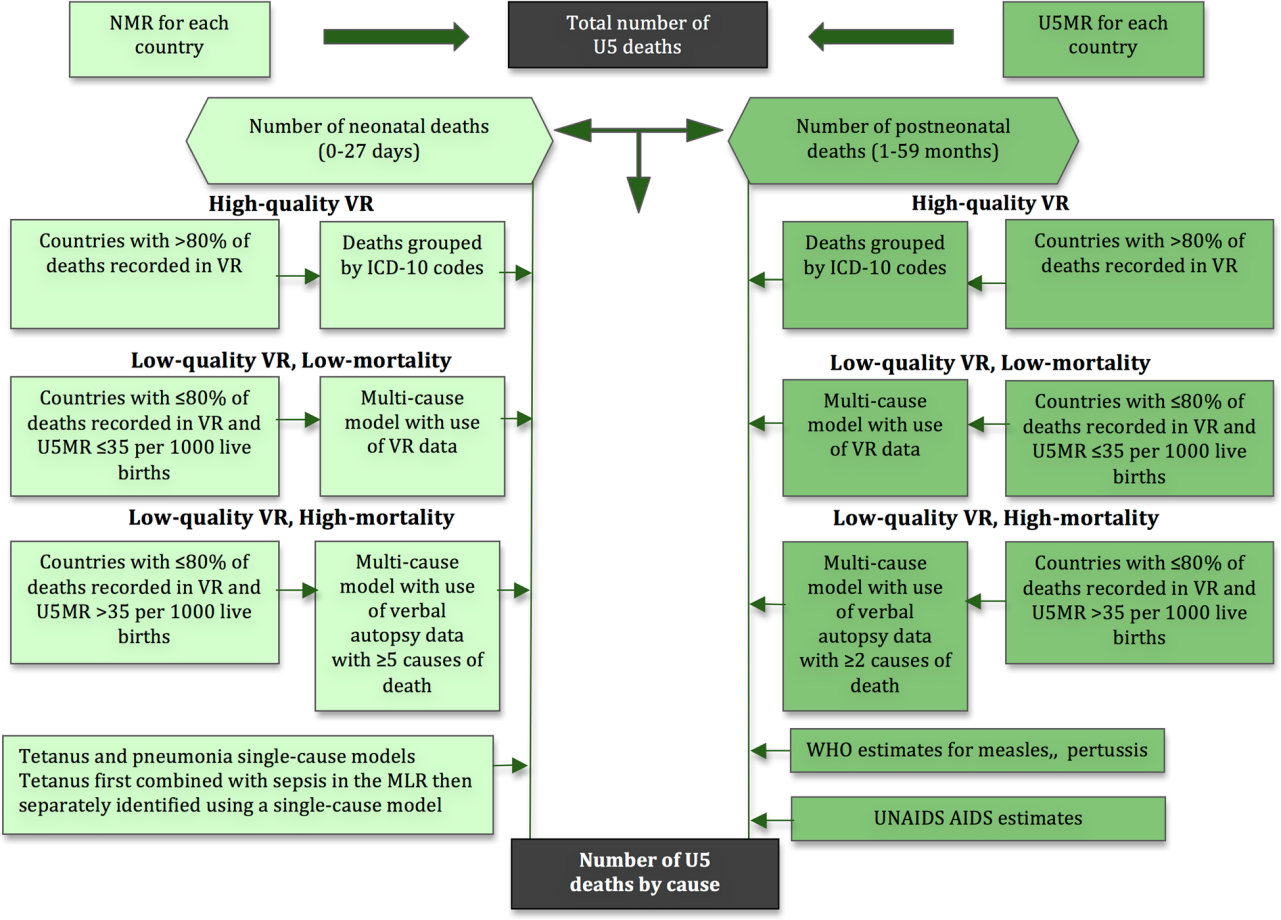
**CHERG analytical approach for estimating the burden of disease in children under five in 2010 (adapted from Black 2010).**

The CHERG approach used the following steps 1) data source identification, 2) data processing, 3) use of one of three models to estimate neonatal and postneonatal mortality separately, depending on mortality level, disease profile, and data availability, 4) development of a disease-specific model to estimate neonatal pneumonia and tetanus, as well as measles and pertussis among older children, and 5) adjusting deaths to sum to the total U5 mortality (Figure 5) [6].

To estimate the total number of deaths due to pneumonia and diarrhea, CHERG used VR data if the country reported >80% VR coverage, had low U5 mortality (defined as ≤35 deaths per 1,000 live births) and the data were of high quality (defined as having a plausible distribution of deaths by cause and limited use of improbable causes of death, or if the causes of death could be categorized for analysis). The vital registration multi-cause model (VRMCM) model for postneonates (children 1–59 months of age) included data from 56 countries for a total of 578 data points.

To estimate the total number of deaths in countries with high mortality and low VR coverage, CHERG identified VA studies using systematic literature reviews. Studies for estimating deaths in children 1–59 months had to be community-based, include at least two causes of death, be conducted after 1979 for 12 months or a multiple of 12 months (due to the seasonality of many infectious causes of death), document at least 25 deaths in children U5, have each death counted only once, and have fewer than 25% of deaths due to unknown causes [7]. This process identified 74 studies from 33 countries for a total of 113 data points. For the neonatal verbal autopsy multi-cause model (VAMCM), VA studies were either community- or hospital-based, conducted for at least 12 months, followed the child for 7 or 28 days after birth, included four or more of the six modeled causes of death, had fewer than 25% of deaths due to unknown causes, and had cause of death (COD) assignment completed by a skilled birth attendant, post mortem, or VA [17]. This process identified 89 studies in 34 high-mortality countries. Neonatal pneumonia was modeled using a separate set of 36 studies in which neonatal pneumonia was reported separately from other neonatal infections or causes of respiratory distress, although the clinical ability to make this distinction is recognized as challenging [7].

To estimate the number of pneumonia deaths due to pneumococcus and *Haemophilus influenzae* type B (Hib), CHERG relied on meta-analyses of vaccine efficacy trials conducted by O’Brien et al. [15] and Watt et al. [9,16]. O’Brien et al. used four vaccine trials to estimate mortality due to pneumococcus [18-21]. A recently reported fifth trial was added in the CHERG analysis (the COMPAS trial) [22]. For Hib, Watt et al. [16] included four studies, one each from the Gambia (individually randomized) [23], Indonesia (cluster randomized) [24], Bangladesh (case control) [25], and Chile (open cluster randomized) [26]. Rudan et al. [9] estimated the fraction of deaths due to pneumococcus and Hib based upon the assumption that the fraction of all cases due to each etiologic agent was equal to the fraction of deaths due to that etiologic agent. Through the systematic review they estimated that 33.0% and 21.3% of pneumonia deaths were due to pneumococcus and Hib, respectively [9].

To estimate the number of deaths due to diarrhea etiologies, CHERG used studies conducted between 1991 and 2011 that lasted at least 12 months, involved children U5 hospitalized for diarrhea, and tested all patients or included a representative sample of hospitalized patients. Studies were excluded if they were conducted during diarrheal outbreaks, did not differentiate between inpatients and outpatients, only focused on special populations, or did not use adequate laboratory techniques. In total, 163 articles and 31 WHO Rotavirus Surveillance Network data points were used in calculating etiology-specific mortality.

#### Data sources discussion

The first major difference between the two groups’ approaches was their inclusion criteria for VR data. CHERG only included data that were >80% complete, met a threshold of adequate quality, and were from low U5 mortality countries whereas the GBD 2010 final model only included VR data that were >60% complete and did not exclude VR data based on a country’s U5 mortality rate. Therefore, VR data in both the CHERG and GBD 2010 models informed estimates in primarily lower mortality, higher income countries where the burden due to pneumonia and diarrhea is generally lower. Tables 1, 2 and 3 and Additional file 1 present the differences in the estimates based on these two approaches. The differences at the country-level can be strikingly large. For example in Nigeria, CHERG estimated 144,596 deaths due to pneumonia and GBD 2010 estimated 64,238 due to LRI in 2010, a 76% difference (Additional file 1).

The two approaches highlight the need to balance the desire to increase the number of data points to inform the models with concerns that incomplete data may contain substantial biases. Bias is introduced by use of the VR data, which represent populations that experience different cause-specific mortality fractions than the population that is not recorded by VR. By including incomplete VR data, modelers run the risk of a biased representation of the cause-specific mortality fractions for the entire population. While the use of more data will increase the apparent precision and narrow the uncertainty interval around mortality estimates, the central estimate itself may be less accurate (in terms of the estimate reflecting the “true” cause-specific mortality fractions). In other words, if VR systems systematically under- or over-report pneumonia or diarrhea as a cause of death, this will not be captured in the uncertainty interval. While theoretically a source of potential bias, the sensitivity analyses conducted for the GBD 2010 study showed that there was very little change in the estimates, irrespective of what cut point was used for VR data. This is partially due to few high pneumonia and diarrhea burden countries providing any VR data in the models.

The second major difference is that GBD 2010 included observational studies to estimate the burden of pneumonia according to specific etiologies, whereas CHERG relied solely on the results of vaccine efficacy studies to produce estimates. CHERG adopted a counterfactual approach and estimated the fraction of radiologically confirmed pneumonia cases prevented by Hib and *pneumococcal* vaccines in randomized trials (the vaccine probe approach) to estimate total number of deaths due to Hib and *pneumococcal* pneumonia. A counterfactual approach assesses what would have happened in the absence of interventions to prevent disease. This approach is strengthened when the unvaccinated control group is large, as was the case in the vaccine trials that contributed to the estimates. GBD 2010 used a categorical approach, and their methods operated under the assumption that although trial data is superior to observational data, the regional variation detected in observational studies warranted their inclusion in the etiology models. To correct for this difference in the quality of the data, GBD 2010 included observational data, but weighted the vaccine efficacy trial data by a factor of 10 [6]. CHERG’s methods did not include data from observational studies because observational studies lack sensitivity for determining bacterial etiology and are non-specific for determining viral etiology, and therefore tend to underestimate the contribution of bacterial pathogens and overestimate the contribution of viral pathogens to pneumonia mortality. These two approaches resulted in vastly different global etiologic fractions of pneumonia deaths due to pneumococcus: CHERG estimated 32.7% and GBD 2010 estimated 19.8% (Table 2).

In addition, upon assessment of the data, CHERG inferred that there is little evidence of regional variation in etiologies, so the CHERG model assumed global consistency in mortality due to pneumococcus and Hib after adjustment for vaccine coverage. Finally, the CHERG method inferred that trial data from Asia, Africa, North America, and South America represent a wide range of geographic areas while showing consistent findings. It is highly unlikely that there is no regional variation in the etiologies of pneumonia and diarrhea, but given a paucity of quality data for the etiologies, estimating the regional variation can be challenging. Thus far, modeling groups have yet to develop methods to incorporate informative observational studies while addressing their inherent biases. Therefore, both approaches can lead to inaccurate estimates; inclusion of observational data introduces substantial errors in etiologies, whereas restriction to vaccine studies results in oversimplification.

### Drivers of the differences: data processing

After data are selected for models, they are often processed to improve the validity of the data before modeling, including transformations, reassignment of cause of death, and removal of outliers to improve the quality and reduce the potential influence of a single or inaccurate data point before modeling. Models that use the same data but perform different transformations can produce very different results.

Both GBD 2010 and CHERG improved the comparability across different datasets by mapping older versions of the International Classification of Disease (ICD) to the ICD-10 codes. GBD 2010 used a detailed algorithm to reassign non-specific or undefined ICD codes, referred to as “garbage codes,” (e.g., fever or cardiac arrest) to logical causes of death. For example, 10.07% of deaths attributed to the non-specific codes of fever, malaise, febrile, and convulsions of unknown origin were reassigned to LRI. Next, the dataset was split into 20 age/sex groups and the data were smoothed to account for instances where specific deaths in a country were very rare. Finally, the compiled dataset was assessed for outliers. Outliers were defined using a subjective set of criteria that were determined by subject-matter experts as having a large inconsistency with other sources in the same country or from the same region.

CHERG reassigned some non-specific COD codes for neonates; classified all malnutrition deaths as “other causes”; grouped neonatal deaths into one of six categories for estimates: preterm birth complications, peripartum-related complications, infectious causes including pneumonia, tetanus, sepsis, and meningitis, congenital abnormalities, other neonatal disorders, and diarrhea; and grouped deaths among children aged 1–59 months into one of eight categories: pneumonia, injury, malaria, meningitis, diarrhea, causes originating from the perinatal period, congenital malformation, and other causes.

GBD 2010 and CHERG took different approaches to coding neonatal COD. Assignment of COD is particularly difficult for neonates due to a lack of VA studies and the general difficulty that clinicians have differentiating deaths due to pneumonia, sepsis, and meningitis. GBD 2010 estimated 732,000 deaths due to these three causes combined, and similarly CHERG estimated 718,000 deaths. The two groups differ, however, in how they split the deaths between the three causes: GBD 2010 attributed 193,131 (26%) deaths to pneumonia through their CODEm model; CHERG used 36 studies to separate neonatal pneumonia deaths from the broader category of neonatal infections and estimated 325,000 (45%) deaths due to pneumonia. The difference between the two groups’ estimates for neonatal pneumonia is 131,869 deaths or 24% of the difference between the two groups’ overall U5 pneumonia mortality estimates.

#### Data processing discussion

Clearly, GBD 2010 and CHERG used different data processing procedures and therefore attributed the causes of neonatal death very differently. This is likely due to the difficulty in assigning COD in neonates based on VA studies and VR data. These differences in neonatal COD assignments have large differences in the proportion of neonatal infections attributed to pneumonia, which consequently account for around a quarter of the difference in the overall estimates for U5 pneumonia mortality. This illustrates the role of subjective decision making in situations where there is poor and limited data on COD. With such little understanding about the causes of death in neonates, there will be increased uncertainty around the estimates. Furthermore, removal of outliers is a problematic statistical technique that can introduce bias into a dataset and subsequent model estimates. It is not possible to evaluate which estimate is more accurate, however this example highlights the importance of data processing, how it can lead to large differences in the estimates, and the need for transparency about data processing decisions.

### Drivers of the differences: risk factor data

Given the paucity of data available for estimating the mortality burden due to pneumonia and diarrhea in many countries, both GBD 2010 and CHERG used data on risk factors and correlates of pneumonia and diarrhea (referred to as covariates by modelers) to further inform their models in addition to regional/super-regional effects. Inclusion of covariates can improve a model’s accuracy and precision in mortality estimates. A full list of the covariates used by each modeling group is available in Tables 4 and 5.

**Table 4:** **Covariates used to estimate pneumonia/LRI and its etiologies in children under five in 2010/2011** [6,7]

	**CHERG**						**GBD 2010**
	**Neonatal**				**Postneonatal**		**U5**
	**Severe infection**		**Pneumonia**				**Lower respiratory infection (LRI)**
	**VRMCM** ^**a**^	**VAMCM** ^**b**^	**VRMCM** ^**a**^	**VAMCM** ^**b**^	**VRMCM** ^**a**^	**VAMCM** ^**b**^	**CODEm** ^**c**^
**Covariates for pneumonia/LRI U5 mortality**	*Early neonatal:*	Neonatal mortality rate	*Early neonatal:*	Female literacy	All model covariates^d^	All model covariates^d^	Health care access
	Access to antenatal care	DPT vaccination coverage	Female literacy	Low birth weight			Hib3 vaccination coverage (country-level)
	Female literacy	Quad-DPT vaccination coverage	Neonatal mortality rate	Births with skilled attendant			Access to sanitation
	Indicator of the EMRO region	Period (early/late/overall)	Births with skilled attendant	Tetanus toxoid vaccine coverage at birth			Access to water
	*Late neonatal:*		Indicator of the EMRO region				Malnutrition in under 2
	Female literacy		*Late neonatal:*				Education
	U5 mortality rate		Access to antenatal care				Population density
	Births with skilled attendant		Indicator of the EMRO region				
	Low birth weight		General fertility rate				
	Indicator of the EMRO region		Births with skilled attendant				
**Covariates for pneumonia/LRI etiologic fractions**	Pneumococcal vaccination coverage						Inpatient or outpatient/community-based settings (study-level)
							Hib3 vaccination coverage (country-level)
							Health system access (country-level)

^a^Vital registration multi-cause model, ^b^verbal autopsy multi-cause model, ^c^cause of death ensemble model, ^d^for details, see Liu et al., Lancet, 2012 web appendices.

**Table 5:** **Covariates used to estimate diarrhea and its etiologies in children under five in 2010** [6,7]

	**CHERG**				**GBD 2010**
	**Neonatal**		**Postneonatal**		**U5**
	**VRMCM** ^**a**^	**VAMCM** ^**b**^	**VRMCM** ^**a**^	**VAMCM** ^**b**^	**CODEm** ^**c**^
**Covariates for diarrhea U5 mortality**	Diarrhea not estimated in VRMCM due to few diarrhea deaths	Neonatal mortality rate	U5 population	Mid study year	Health care access
		DPT vaccination coverage	Coverage of the third dose of diphtheria pertussis and tetanus vaccine (DPT3)	% urban population	Rota virus coverage
		Quad-DPT vaccination coverage	WHO Europe Region vs. other WHO regions	U5 mortality rate	Access to sanitation
		Period (early/late/overall)	Gross national income (per capita)	Oral rehydration therapy coverage	Access to water
					Malnutrition in under 2
					Education
					Population density
					GDP (lag-time)
**Covariates for diarrhea etiologic fractions**	n/a				National coverage or subnational but nationally representative (study-level)
					Inpatient or outpatient/community-based settings (study-level)
					Number of pathogens tested for (study-level)
					Lag-distributed income (country-level)

^a^Vital registration multi-cause model, ^b^verbal autopsy multi-cause model, ^c^cause of death ensemble model.

The inclusion of individual covariates can alter estimates dramatically. For example, the CHERG model did post-hoc adjustment for insecticide treated net (ITN) coverage and Hib vaccine coverage in their VAMCM, which modeled deaths in 79 high mortality countries and excludes deaths due to measles and AIDS-mediated diseases. The proportion of deaths due to pneumonia when adjusted for both covariates was 23% compared to 20% when adjusting for only Hib vaccination in the VAMCM. This demonstrates the power of adding a single covariate to the model and its subsequent change to the cause-specific mortality fractions.

While both GBD 2010 and CHERG examined many covariates, there is little overlap between those included in the two groups’ models. There are some potentially influential covariates that were not included by either group. For example, GBD 2010 did not include the percentage of population at risk for malaria for their pneumonia or diarrhea models, but did include malaria risk in their malaria model (Tables 4 and 5). CHERG included a measure of population at risk for malaria as part of their malaria equation in the VAMCM. This may be an important covariate to include in future studies, as malaria deaths are often misattributed to pneumonia or diarrhea, or pneumonia and diarrheal deaths are misattributed to malaria [27,28]. Inclusion of malaria covariates could increase the proportion of cases due to pneumonia and diarrhea as control measures reduce malaria deaths. In addition, numerous observational studies and meta-analyses have noted that breastfeeding is associated with reduced risk of both pneumonia and diarrhea mortality [29-33]. The inclusion of the percentage of women exclusively breastfeeding may improve model predictions and therefore warrants consideration.

The reliance on covariate data in the absence of high quality VA and VR COD data to predict mortality due to pneumonia and diarrhea reinforces the need for quality country-specific COD data and highlights the need for better covariate data sources that measure target variables accurately. Additionally, there is a need for longitudinal covariate data to assess trends in mortality due to pneumonia and diarrhea. Regardless of the number of covariates used, a modeling approach will never be as accurate as estimating the burden of disease from high quality and high coverage VA/VR data sources from strong national systems.

### Etiology model assumptions

Modeling the number and causal fractions of deaths due to pneumonia and diarrhea etiologies is extremely difficult due to insensitive/incomplete diagnostics and the lack of studies that provide estimates of the burden by etiology. GBD 2010 estimated 21.7% and 19.8% compared to CHERG’s estimated 15.7% and 32.7% of deaths due to Hib and pneumococcus, respectively. Furthermore, GBD 2010 estimated that RSV was responsible for 27.6% of pneumonia deaths. Due to limitations in the data available for estimating the etiologies of pneumonia and diarrheal deaths, both GBD 2010 and CHERG made several assumptions. Differences in assumptions and their consequences may explain some of the differences in the two groups’ estimates.

#### Pneumonia/LRI etiologies

To estimate the causal fraction and number of deaths due to specific etiologies of pneumonia/LRI, GBD 2010 and CHERG both assumed that all cases admitted to hospital were severe pneumonia cases, and that the etiologic proportion of chest radiography positive cases was a reasonable surrogate for fatal cases in the community. Both groups also assumed that in data from vaccine efficacy trials, the fraction of radiologically confirmed pneumonia cases that were assigned to Hib or pneumococcus was a reasonable surrogate for that fraction in fatal cases.

The groups differed in their assumptions on the proportion of pneumonia due to different etiologies. GBD 2010 allowed for regional variation in the causal fraction of pneumonia due to pneumococcus, Hib, RSV, influenza virus, and other etiologies. This regional variation was informed by 45 additional observational studies included in their updated systematic review of the literature. In instances where children were found to have multiple infections (e.g., pneumococcal pneumonia and RSV), they used a probabilistic model to assign the etiologic fractions. The probabilistic model used the proportion of pathogens in moderate to severe cases to assign pneumonia etiologies to each death and the proportion of pathogens in mild pneumonia to assign pneumonia etiologies to each case. The model was not informed by any hierarchy of causes of death. Children with no detected pathogen were not included in the etiologic fraction estimation. In contrast, CHERG’s methodology was informed by etiologic estimates, which assumed that the proportion of deaths due to both pneumococcal pneumonia and Hib did not vary by region (14–16). Instead, they estimated one proportion for pneumococcus and one for Hib and adjusted these proportions by the vaccine coverage for pneumococcus and Hib, respectively.

Both groups made assumptions about mortality due to viral pneumonia specifically caused by influenza virus and RSV. GBD 2010 built upon the work of Nair et al. for both RSV and influenza mortality estimates by supplementing those findings with data from observational studies of viral etiologies. CHERG noted that the estimates from two reviews of disease caused by RSV and influenza virus, based on work conducted by Nair et al. [34,35], could not provide point estimates or confidence intervals, and consequently global and country-level etiologic estimates could not be made. Therefore, CHERG did not make country-level mortality estimates for RSV and influenza based on this study [9].

The two groups made different assumptions regarding the patterns of mortality by age. For GBD 2010, DisMod-MR, a multi-regression model used for etiology estimates, enabled the cause-specific mortality fraction to vary with age [36]. GBD 2010 made further assumptions regarding the proportion of RSV; due to a lack of data for RSV, they scaled the sum of the proportion of LRI due to RSV in ages 2–4 years to 25% of the sum of the proportion in ages 0–1 year. This age difference was based on the pattern of hospital admissions for RSV pneumonia observed in US hospital data [6]. CHERG did not produce age-specific mortality estimates beyond the number of deaths for pneumococcal pneumonia and Hib in children U5.

#### Diarrhea etiologies

GBD 2010 and CHERG made several similar assumptions about diarrhea etiologies. Both assumed that hospitalized cases were more severe than those seen in the community and that the etiologic proportions seen in severe cases were representative of causes of diarrheal deaths.

Both groups adjusted their etiologic fractions to sum to one, which in effect allows only one etiology per diarrheal death, but the groups applied this constraint differently. GBD 2010 acted on this assumption in two ways: 1) where the cause of diarrheal death was unknown in a study, GBD 2010 did not include this death in the denominator when calculating the etiologic fractions and instead assumed that the death was due to a completely unknown etiology (i.e., an untested etiology); 2) when a study detected multiple diarrheal pathogens, GBD 2010 used the proportion of pathogens in moderate to severe cases to assign diarrheal etiologies to each death and the proportion of pathogens in mild cases to assign diarrhea etiologies to each case, and did not use a hierarchy of causes of death. CHERG reported both total age-adjusted etiologic proportions (which summed to 138% of deaths) and the proportions after scaling them to add to 100%. They also allowed unknown pathogens to exist as a category, but only used studies that examined eight or more pathogens. Table 3 illustrates the differences in the cause-specific mortality fractions for diarrheal deaths. The unknown pathogen group accounted for 24.5% of deaths after scaling. Direct comparison of the cause-specific mortality fractions is difficult because CHERG generated estimates for 13 pathogens while GBD 2010 generated estimates for only ten pathogens, and the two groups’ pathogen lists do not completely overlap.

GBD 2010 used the assumption that there were regional differences in the etiologic fractions and attempted to account for this with the country-specific covariates and by varying the priors used in the Bayesian models. This enabled GBD 2010 to produce regional estimates of deaths from each cause. Although CHERG did stratify the attributable fraction from each pathogen by WHO region, the group did not consider there to be a sufficient number of studies to draw conclusions from the estimates.

#### Etiologies discussion

The major difference in modeling approaches between the two groups was inclusion of observational study data for estimating pneumonia etiologies. GBD 2010 included observational data, which showed regional variation, but this regional variation may be due to less sensitive laboratory measures often used in observational studies.

Using observational studies relies on pneumonia diagnostics. Pneumonia etiologies are notoriously difficult to diagnose because the gold standard of diagnosis is identification of the pathogen from lung tissue. Given the invasiveness of this procedure, gold standard data are often lacking. In addition, treatment with antibiotics before specimen collection can make isolation of the bacterial agent difficult. Despite these limitations, observational studies are more frequently conducted in more geographic regions than expensive vaccine trials, and therefore provide information about areas where vaccine trials are not feasible. Given these factors, the GBD 2010 team utilized observational studies to provide key data in building a comprehensive understanding of mortality related to pneumonia etiologies.

Estimating mortality due to the etiologies of pneumonia and diarrhea relies on secondary endpoints and multiple assumptions about those endpoints. Specifically, use of observational studies requires the assumption that hospitalized cases represent severe cases, and the etiologies of severe cases match the etiologies of deaths. For RSV, this assumption may be false, and many hospitalized RSV cases are not severe pneumonia cases. This results in an overestimation in the number of severe RSV cases, and consequently an overestimation in the number of RSV deaths. The use of vaccine probe studies relies on the reduction of chest radiography confirmed pneumonia as the measure of the reduction of cases due to the specific pathogen in the vaccine. This counterfactual approach can lead to the overestimation of the burden due to specific pathogens if it is based on trial data where the specific pathogen is more common, or can result in an underestimation if the trials are in areas where the pathogen is less common.

Both groups also treated data points for U5 mortality due to unknown and multiple pathogens differently. As noted above, definitively identifying the causative diarrheal agent in stool is challenging because of the natural carriage of organisms and the fact that it is common to find multiple organisms in a single sample. For example, the recent Global Enterics Multi-Center Study (GEMS) found that 45% of diarrhea cases and 31% of non-diarrheal controls had two or more potentially pathogenic agents in their stool [37]. GBD 2010 excluded diarrheal deaths with an unknown etiology. This may systematically underestimate deaths from an etiology that is difficult to detect and overestimate the contribution of other pathogens. By including unknown etiologies as a group, CHERG avoids the problem of overestimation, but cannot address the problem of underestimation. For deaths due to multiple pathogens, GBD 2010 assigned the COD probabilistically, based on the known etiologic fractions, whereas CHERG’s scaling approach effectively made each of the multiple organisms found, equally likely to have caused that disease. The latter assumption may be problematic, as CHERG has acknowledged, and efforts should continue to appropriately assign COD [10]. For diarrhea specifically, priority should be given to studies that include a control group or quantify pathogen load to better ascertain the true etiology of diarrhea.

### Conclusions and recommendations

GBD 2010 and CHERG’s mortality estimates represent enormous undertakings by some of the world’s leading experts in mortality estimation, yet these separate efforts resulted in markedly different estimates for country-level and etiology-specific U5 mortality for pneumonia and diarrhea. The differences in the estimates highlight firstly that limited data available for estimating causes of death, and secondly that decisions on how models are parameterized can have important effects on estimates. Given these two realities, greater openness and transparency is needed in all aspects of the modeling process, including timely access to all publically available data sources. CHERG included a list of sources with their publications. Access to all sources used in the GBD 2010 study was not initially provided, but has since been made available on the IHME website via an online visualization tool: http://viz.healthmetricsandevaluation.org. In addition to timely access to sources, it is important to publish the covariates considered for each model (in addition to the final covariates used), any data processing employed, and when useful, interim model results. Transparency of methods and easy access to source data, best represented in the Rudan et al. estimates of pneumonia etiologies, are key to maintaining quality and accuracy in data used to inform our public health decision-making [9]. Guidelines, similar to those used for reporting epidemiological studies (e.g., STROBE and CONSORT), are needed for reporting modeling work.

Unfortunately, even the “best” current data are problematic due to inherent difficulties in determining the causative agent for a high proportion of cases, even under optimal conditions using the best laboratory methods. The lack of high-quality data necessitated several assumptions and reliance on covariates for building the models. Currently, the countries experiencing the greatest mortality due to pneumonia and diarrhea have the weakest VR systems, and very few, if any, VA studies conducted in the past 30 years. While the improvement of VR systems and the corresponding collection of COD data for all citizens are the goal for every country, the necessary infrastructure investments make this unachievable in the short-term. As more data are needed specifically from high mortality countries and underrepresented high-risk groups, VA and other field-based approaches that can be validated with shared datasets should be prioritized.

Having varying estimates from multiple modeling groups can be beneficial for both the scientific and policy-making communities. Through the comparison of modeling approaches and estimates, we improve our understanding of how specific assumptions, inclusion of different risk factors, and mortality and etiology data sources influence each model’s estimates. However, a framework for understanding drivers of differences and transparency in data sourcing and processing is a prerequisite.

In October 2013, the BMGF convened an expert meeting in order to better understand the discrepancies between the estimates discussed in this paper and to discuss emerging innovations, plans for coordination, and specific improvements to the models, which could be used to create future mortality estimates. Members of IHME, CHERG, and other experts are committed to further investigation into the drivers of the differences by systematically altering the data and covariates included in the models to test the extent of their influence. IHME specifically agreed to conduct sensitivity analyses excluding VR data from the model in similar patterns used by CHERG to demonstrate whether the use of this VR data explains the differences in the estimates or if the differences are due to the modeling procedures themselves. CHERG had previously shared estimates and most of their study input data, and plans to make publically available more detailed information on the model covariates and modeling process. The group also discussed the merits of moving away from calculating explicit pneumonia and diarrhea etiologic fractions, with high uncertainty biases, towards a counterfactual approach, which allows for assessment of the impact of specific interventions by approximating what would be observed in the absence of that intervention. IHME determined to use the counterfactual approach for estimating mortality from pathogens due to specific etiologies to overcome bias of other methods in GBD 2.0. This approach will estimate the reduction in deaths if we removed an individual etiology from the globe. Lastly, the meeting attendees committed themselves to future collaboration, affirming the value of face-to-face meetings to harmonize assumptions and share methodologies before publications. The newly-formed Reference Group for Health Statistics hosted by WHO and the Independent Advisory Committee for GBD studies will serve as critical forums to share approaches, assess utility, and ensure innovation and advancement of quality data. As IHME released GBD 2013 in late 2014 and CHERG released new 2012-based estimates in late 2014, deeper understanding will improve our interpretation of estimates, empowering governments, non-governmental organizations, and the broader international community to appropriately prioritize health interventions and make more efficient progress toward achieving the Millennium Development Goals.

## Additional files

Additional file 1: **GBD 2010 [** 6 **] and CHERG [** 7 **] total U5 mortality estimates due to all causes, pneumonia/LRI, and diarrhea by country in 2010 by WHO region.**

Additional file 2: **Data sources for pneumonia etiology mortality models for GBD 2010 and CHERG.**

Additional file 3: Table S3. Sources for the diarrhea etiology models used by GBD 2010 [6] and CHERG [10].

Additional file 4: **Supplementary Materials: Analytical models for estimating cause-specific mortality.**

## Abbreviations

ARI: Acute respiratory infection
BMGF: Bill & Melinda Gates Foundaiton
CHERG: Child Health and Epidemiology Research Group
COD: Cause of death
CoDCorrect: Cause of death correction model
CODEm: Cause of death ensemble model
CONSORT: Consolidated standards of reporting trials
DisMod: A computer software package for modeling causes of death
DPT3: Diphtheria pertussis tetanus three series vaccine
GEMS: Global enteric multicenter study
GBD 2010: Global burden of disease 2010
Hib: *Haemophilus influenza* type b vaccine
ICD: International classification of disease
IGME: Inter-agency group for child mortality estimation
IHME: Institute for health metrics and evaluation
ITN: Insecticide treated net
LRI: Lower respiratory infection
MDG: Millennium development goals
RSV: Respiratory syncytial virus
STROBE: Strengthening the reporting of observational studies in epidemiology
U5: Under-five
U5MR: Under five mortality rate
UN: United nations
VA: Verbal autopsy
VAMCM: Verbal autopsy multi-cause model
VR: Vital registration
VRMCM: Vital registration multi-cause model

## References

[1] Murray CJ, Lopez AD (1994). Global and regional cause-of-death patterns in 1990. Bull World Health Organ.

[2] Murray CJ, Lopez AD (1997). Mortality by cause for eight regions of the world: Global Burden of Disease Study. Lancet.

[3] Child Mortality Fact Sheet [http://www.who.int/pmnch/media/press_materials/fs/fs_mdg4_childmortality/en/]

[4] UN Millennium Project (2005). Investing in Development A Practical Plan to Achieve the Mellennium Development Goals.

[5] Wang H, Dwyer-Lindgren L, Lofgren KT, Rajaratnam JK, Marcus JR, Levin-Rector A (2012). Age-specific and sex-specific mortality in 187 countries, 1970–2010: a systematic analysis for the Global Burden of Disease Study 2010. Lancet.

[6] Lozano R, Naghavi M, Foreman K, Lim S, Shibuya K, Aboyans V (2012). Global and regional mortality from 235 causes of death for 20 age groups in 1990 and 2010: a systematic analysis for the Global Burden of Disease Study 2010. Lancet.

[7] Liu L, Johnson HL, Cousens S, Perin J, Scott S, Lawn JE (2012). Global, regional, and national causes of child mortality: an updated systematic analysis for 2010 with time trends since 2000. Lancet.

[8] Black RE, Cousens S, Johnson HL, Lawn JE, Rudan I, Bassani DG (2010). Global, regional, and national causes of child mortality in 2008: a systematic analysis. Lancet.

[9] Rudan I, O’Brien KL, Nair H, Liu L, Theodoratou E, Qazi S (2013). Epidemiology and etiology of childhood pneumonia in 2010: estimates of incidence, severe morbidity, mortality, underlying risk factors and causative pathogens for 192 countries. J Glob Health.

[10] Lanata CF, Fischer-Walker CL, Olascoaga AC, Torres CX, Aryee MJ, Black RE (2013). Global causes of diarrheal disease mortality in children <5 years of age: a systematic review. PLoS One.

[11] Hill K, You D, Inoue M, Oestergaard MZ (2012). Child mortality estimation: accelerated progress in reducing global child mortality, 1990–2010. PLoS Med.

[12] Alkema L, You D (2012). Child mortality estimation: a comparison of UN IGME and IHME estimates of levels and trends in under-five mortality rates and deaths. PLoS Med.

[13] Foreman KJ, Lozano R, Lopez AD, Murray CJ (2012). Modeling causes of death: an integrated approach using CODEm. Popul Health Metr.

[14] Black S, Shinefield H, Fireman B, Lewis E, Ray P, Hansen JR (2000). Efficacy, safety and immunogenicity of heptavalent pneumococcal conjugate vaccine in children. Pediatr Infect Dis J.

[15] O’Brien KL, Wolfson LJ, Watt JP, Henkle E, Deloria-Knoll M, McCall N (2009). Burden of disease caused by Streptococcus pneumoniae in children younger than 5 years: global estimates. Lancet.

[16] Watt JP, Wolfson LJ, O’Brien KL, Henkle E, Deloria-Knoll M, McCall N (2009). Burden of disease caused by Haemophilus influenzae type b in children younger than 5 years: global estimates. Lancet.

[17] Lawn JE, Wilczynska-Ketende K, Cousens SN (2006). Estimating the causes of 4 million neonatal deaths in the year 2000. Int J Epidemiol.

[18] Cutts FT, Zaman SM, Enwere G, Jaffar S, Levine OS, Okoko JB (2005). Efficacy of nine-valent pneumococcal conjugate vaccine against pneumonia and invasive pneumococcal disease in The Gambia: randomised, double-blind, placebo-controlled trial. Lancet.

[19] Klugman KP, Madhi SA, Huebner RE, Kohberger R, Mbelle N, Pierce N (2003). A trial of a 9-valent pneumococcal conjugate vaccine in children with and those without HIV infection. N Engl J Med.

[20] Shinefield H, Black S, Ray P, Fireman B, Schwalbe J, Lewis E (2002). Efficacy, immunogenicity and safety of heptavalent pneumococcal conjugate vaccine in low birth weight and preterm infants. Pediatr Infect Dis J.

[21] Lucero MG, Nohynek H, Williams G, Tallo V, Simões EA, Lupisan S (2009). Efficacy of an 11-valent pneumococcal conjugate vaccine against radiologically confirmed pneumonia among children less than 2 years of age in the Philippines: a randomized, double-blind, placebo-controlled trial. Pediatr Infect Dis J.

[22] Afonso ET, Minamisava R, Bierrenbach AL, Escalante JJC, Alencar AP, Domingues CM (2013). Effect of 10-valent pneumococcal vaccine on pneumonia among children, Brazil. Emerg Infect Dis.

[23] Mulholland K, Hilton S, Adegbola R, Usen S, Oparaugo A, Omosigho C (1997). Randomised trial of Haemophilus influenzae type-b tetanus protein conjugate vaccine [corrected] for prevention of pneumonia and meningitis in Gambian infants. Lancet.

[24] Gessner BD, Sutanto A, Linehan M, Djelantik IGG, Fletcher T, Gerudug IK (2005). Incidences of vaccine-preventable Haemophilus influenzae type b pneumonia and meningitis in Indonesian children: hamlet-randomised vaccine-probe trial. Lancet.

[25] Baqui AH, El Arifeen S, Saha SK, Persson L, Zaman K, Gessner BD (2007). Effectiveness of Haemophilus influenzae type B conjugate vaccine on prevention of pneumonia and meningitis in Bangladeshi children: a case–control study. Pediatr Infect Dis J.

[26] Levine O, Lagos R, Munoz A, Villaroel J, Alvarez A, Abrego P (1999). Defining the burden of pneumonia in children preventable by vaccination against Haemophilus Influenzae type b. Pediatr Infect Dis J.

[27] O’Dempsey TJ, McArdle TF, Laurence BE, Lamont AC, Todd JE, Greenwood BM (1993). Overlap in the clinical features of pneumonia and malaria in African children. Trans R Soc Trop Med Hyg.

[28] Källander K, Nsungwa-Sabiiti J, Peterson S (2004). Symptom overlap for malaria and pneumonia–policy implications for home management strategies. Acta Trop.

[29] Lamberti LM, Fischer Walker CL, Noiman A, Victora C, Black RE (2011). Breastfeeding and the risk for diarrhea morbidity and mortality. BMC Public Health.

[30] Black RE, Allen LH, Bhutta ZA, Caulfield LE, de Onis M, Ezzati M (2008). Maternal and child undernutrition: global and regional exposures and health consequences. Lancet.

[31] Quigley MA, Kelly YJ, Sacker A (2007). Breastfeeding and hospitalization for diarrheal and respiratory infection in the United Kingdom Millennium Cohort Study. Pediatrics.

[32] Yoon PW, Black RE, Moulton LH, Becker S (1996). Effect of not breastfeeding on the risk of diarrheal and respiratory mortality in children under 2 years of age in Metro Cebu, The Philippines. Am J Epidemiol.

[33] Arifeen S, Black RE, Antelman G, Baqui A, Caulfield L, Becker S (2001). Exclusive breastfeeding reduces acute respiratory infection and diarrhea deaths among infants in Dhaka slums. Pediatrics.

[34] Nair H, Brooks WA, Katz M, Roca A, Berkley JA, Madhi SA (2011). Global burden of respiratory infections due to seasonal influenza in young children: a systematic review and meta-analysis. Lancet.

[35] Nair H, Nokes DJ, Gessner BD, Dherani M, Madhi SA, Singleton RJ (2010). Global burden of acute lower respiratory infections due to respiratory syncytial virus in young children: a systematic review and meta-analysis. Lancet.

[36] Barendregt JJ, Van Oortmarssen GJ, Vos T, Murray CJ (2003). A generic model for the assessment of disease epidemiology: the computational basis of DisMod II. Popul Health Metr.

[37] Kotloff KL, Nataro JP, Blackwelder WC, Nasrin D, Farag TH, Panchalingam S (2013). Burden and aetiology of diarrhoeal disease in infants and young children in developing countries (the Global Enteric Multicenter Study, GEMS): a prospective, case–control study. Lancet.

