# Supplementary material for: Deconstructing the differences: a comparison of GBD 2010 and CHERG’s approach to estimating the mortality burden of diarrhea, pneumonia, and their etiologies
Source: BMC Infect Dis. 2015 Jan 16;15:16. doi: 10.1186/s12879-014-0728-4 (PMC4305232; doi:10.1186/s12879-014-0728-4)
Supplement: Supplementary file 1 — GBD 2010 [ 6 ] and CHERG [ 7 ] total U5 mortality estimates due to all causes, pneumonia/LRI, and diarrhea by country in 2010 by WHO region. [file 12879_2014_728_MOESM1_ESM.docx]

**Additional file 1:**

**GBD 2010 [6] and CHERG [7] total U5 mortality estimates due to all causes, pneumonia/LRI, and diarrhea by country in 2010 by WHO region**

|  | | | | | | | | | | Total U5 Morality Estimates | | | | | | | | | | | Pneumonia/LRI** U5 Mortality Estimates | | | | | | | | | | | Diarrhea U5 Mortality Estimates | | | | | | | | | |
| --- | --- | --- | --- | --- | --- | --- | --- | --- | --- | --- | --- | --- | --- | --- | --- | --- | --- | --- | --- | --- | --- | --- | --- | --- | --- | --- | --- | --- | --- | --- | --- | --- | --- | --- | --- | --- | --- | --- | --- | --- | --- |
| Country | **CHERG** | | | | **GBD 2010** | | | | **Difference (CHERG-GBD 2010)** | | | | **% Difference*** | | | **CHERG (Pneumonia)** | | | | **GBD 2010 (LRI**)** | | **Difference (CHERG-GBD 2010)** | | | | **% Difference*** | | | **CHERG** | | | | **GBD 2010** | | | | **Difference (CHERG-GBD 2010)** | | | | **% Difference*** |
| AFRICAN REGION | | | | | | | | | | | | | | | | | | | | | | | | | | | | | | | | | | | | | | | | | |
| Algeria | 26096 | | | | 19724 | | | | 6372 | | | | 28% | | | 3111 | | | | 1284 | | 1827 | | | | 83% | | | 1264 | | | | 523 | | | | 741 | | | | 83% |
| Angola | 121089 | | | | 65322 | | | | 55767 | | | | 60% | | | 20897 | | | | 5710 | | 15187 | | | | 114% | | | 18598 | | | | 8283 | | | | 10315 | | | | 77% |
| Benin | 39043 | | | | 30938 | | | | 8105 | | | | 23% | | | 6744 | | | | 4206 | | 2538 | | | | 46% | | | 3958 | | | | 1675 | | | | 2283 | | | | 81% |
| Botswana | 2299 | | | | 1017 | | | | 1282 | | | | 77% | | | 305 | | | | 152 | | 153 | | | | 67% | | | 141 | | | | 181 | | | | -40 | | | | -25% |
| Burkina Faso | 123343 | | | | 122443 | | | | 900 | | | | 1% | | | 21764 | | | | 19108 | | 2656 | | | | 13% | | | 14648 | | | | 21325 | | | | -6677 | | | | -37% |
| Burundi | 38424 | | | | 29120 | | | | 9304 | | | | 28% | | | 7295 | | | | 3180 | | 4115 | | | | 79% | | | 5605 | | | | 3895 | | | | 1710 | | | | 36% |
| Cameroon | 93191 | | | | 73733 | | | | 19458 | | | | 23% | | | 14364 | | | | 8684 | | 5680 | | | | 49% | | | 12150 | | | | 7353 | | | | 4797 | | | | 49% |
| Cape Verde | 373 | | | | 259 | | | | 114 | | | | 36% | | | 72 | | | | 14 | | 58 | | | | 135% | | | 30 | | | | 25 | | | | 5 | | | | 18% |
| Central African Republic | 23480 | | | | 21296 | | | | 2184 | | | | 10% | | | 3756 | | | | 2171 | | 1585 | | | | 53% | | | 2540 | | | | 2718 | | | | -178 | | | | -7% |
| Chad | 80044 | | | | 82042 | | | | -1998 | | | | -2% | | | 14956 | | | | 12970 | | 1986 | | | | 14% | | | 11234 | | | | 18034 | | | | -6800 | | | | -46% |
| Comoros | 2274 | | | | 1752 | | | | 522 | | | | 26% | | | 408 | | | | 215 | | 193 | | | | 62% | | | 198 | | | | 103 | | | | 95 | | | | 63% |
| Congo | 12762 | | | | 11461 | | | | 1301 | | | | 11% | | | 1842 | | | | 990 | | 852 | | | | 60% | | | 951 | | | | 876 | | | | 75 | | | | 8% |
| Cote d'Ivoire | 80770 | | | | 86550 | | | | -5780 | | | | -7% | | | 11801 | | | | 10584 | | 1217 | | | | 11% | | | 6991 | | | | 16021 | | | | -9030 | | | | -78% |
| DRC | 464583 | | | | 398005 | | | | 66578 | | | | 15% | | | 87007 | | | | 44424 | | 42583 | | | | 65% | | | 59533 | | | | 54867 | | | | 4666 | | | | 8% |
| Equatorial Guinea | 2895 | | | | 3287 | | | | -392 | | | | -13% | | | 396 | | | | 277 | | 119 | | | | 35% | | | 205 | | | | 210 | | | | -5 | | | | -2% |
| Eritrea | 11234 | | | | 13689 | | | | -2455 | | | | -20% | | | 2181 | | | | 2250 | | -69 | | | | -3% | | | 1201 | | | | 1638 | | | | -437 | | | | -31% |
| Ethiopia | 277186 | | | | 216675 | | | | 60511 | | | | 25% | | | 57845 | | | | 33483 | | 24362 | | | | 53% | | | 38535 | | | | 20631 | | | | 17904 | | | | 61% |
| Gabon | 2977 | | | | 2808 | | | | 169 | | | | 6% | | | 332 | | | | 190 | | 142 | | | | 54% | | | 202 | | | | 116 | | | | 86 | | | | 54% |
| Gambia | 6130 | | | | 6216 | | | | -86 | | | | -1% | | | 949 | | | | 943 | | 6 | | | | 1% | | | 555 | | | | 364 | | | | 191 | | | | 42% |
| Ghana | 56893 | | | | 50076 | | | | 6817 | | | | 13% | | | 7467 | | | | 4878 | | 2589 | | | | 42% | | | 4143 | | | | 1605 | | | | 2538 | | | | 88% |
| Guinea | 48448 | | | | 50719 | | | | -2271 | | | | -5% | | | 7885 | | | | 8289 | | -404 | | | | -5% | | | 4808 | | | | 2518 | | | | 2290 | | | | 63% |
| Guinea-Bissau | 8201 | | | | 8370 | | | | -169 | | | | -2% | | | 1458 | | | | 721 | | 737 | | | | 68% | | | 1016 | | | | 944 | | | | 72 | | | | 7% |
| Kenya | 122130 | | | | 92353 | | | | 29777 | | | | 28% | | | 20474 | | | | 14076 | | 6398 | | | | 37% | | | 11461 | | | | 8462 | | | | 2999 | | | | 30% |
| Lesotho | 5084 | | | | 7395 | | | | -2311 | | | | -37% | | | 629 | | | | 1511 | | -882 | | | | -82% | | | 377 | | | | 1524 | | | | -1147 | | | | -121% |
| Liberia | 17067 | | | | 22148 | | | | -5081 | | | | -26% | | | 2383 | | | | 2688 | | -305 | | | | -12% | | | 1521 | | | | 3972 | | | | -2451 | | | | -89% |
| Madagascar | 44650 | | | | 45105 | | | | -455 | | | | -1% | | | 8161 | | | | 5762 | | 2399 | | | | 34% | | | 4627 | | | | 5888 | | | | -1261 | | | | -24% |
| Malawi | 57232 | | | | 67045 | | | | -9813 | | | | -16% | | | 7865 | | | | 9333 | | -1468 | | | | -17% | | | 4025 | | | | 7480 | | | | -3455 | | | | -60% |
| Mali | 124069 | | | | 102909 | | | | 21160 | | | | 19% | | | 24478 | | | | 8252 | | 16226 | | | | 99% | | | 17977 | | | | 7012 | | | | 10965 | | | | 88% |
| Mauritania | 13260 | | | | 8737 | | | | 4523 | | | | 41% | | | 2204 | | | | 1288 | | 916 | | | | 52% | | | 1458 | | | | 1056 | | | | 402 | | | | 32% |
| Mauritius | 232 | | | | 218 | | | | 14 | | | | 6% | | | 20 | | | | 12 | | 8 | | | | 50% | | | 0 | | | | 3 | | | | -3 | | | | -200% |
| Mozambique | 115727 | | | | 102176 | | | | 13551 | | | | 12% | | | 17814 | | | | 10204 | | 7610 | | | | 54% | | | 10696 | | | | 7557 | | | | 3139 | | | | 34% |
| Namibia | 2450 | | | | 2752 | | | | -302 | | | | -12% | | | 294 | | | | 288 | | 6 | | | | 2% | | | 121 | | | | 611 | | | | -490 | | | | -134% |
| Niger | 100190 | | | | 114878 | | | | -14688 | | | | -14% | | | 21691 | | | | 17800 | | 3891 | | | | 20% | | | 14499 | | | | 23383 | | | | -8884 | | | | -47% |
| Nigeria | 868725 | | | | 847207 | | | | 21518 | | | | 3% | | | 143596 | | | | 64238 | | 79358 | | | | 76% | | | 97638 | | | | 55782 | | | | 41856 | | | | 55% |
| Rwanda | 37579 | | | | 26935 | | | | 10644 | | | | 33% | | | 7394 | | | | 3612 | | 3782 | | | | 69% | | | 4447 | | | | 1902 | | | | 2545 | | | | 80% |
| Sao Tome and Principe | 399 | | | | 244 | | | | 155 | | | | 48% | | | 71 | | | | 18 | | 53 | | | | 119% | | | 43 | | | | 25 | | | | 18 | | | | 53% |
| Senegal | 34703 | | | | 29619 | | | | 5084 | | | | 16% | | | 5518 | | | | 2370 | | 3148 | | | | 80% | | | 2975 | | | | 5324 | | | | -2349 | | | | -57% |
| Seychelles | 20 | | | | 20 | | | | 0 | | | | 0% | | | 1 | | | | 3 | | -2 | | | | -100% | | | 0 | | | | 0 | | | | 0 | | | | 0% |
| Sierra Leone | 39100 | | | | 24142 | | | | 14958 | | | | 47% | | | 6755 | | | | 2254 | | 4501 | | | | 100% | | | 4821 | | | | 1452 | | | | 3369 | | | | 107% |
| South Africa | 58817 | | | | 53697 | | | | 5120 | | | | 9% | | | 6717 | | | | 6102 | | 615 | | | | 10% | | | 3181 | | | | 11031 | | | | -7850 | | | | -110% |
| Swaziland | 2610 | | | | 2463 | | | | 147 | | | | 6% | | | 365 | | | | 367 | | -2 | | | | -1% | | | 184 | | | | 663 | | | | -479 | | | | -113% |
| Tanzania | 133471 | | | | 139904 | | | | -6433 | | | | -5% | | | 19835 | | | | 19277 | | 558 | | | | 3% | | | 11391 | | | | 6990 | | | | 4401 | | | | 48% |
| Togo | 19375 | | | | 22267 | | | | -2892 | | | | -14% | | | 3089 | | | | 2760 | | 329 | | | | 11% | | | 1967 | | | | 2613 | | | | -646 | | | | -28% |
| Uganda | 141469 | | | | 100128 | | | | 41341 | | | | 34% | | | 24078 | | | | 8216 | | 15862 | | | | 98% | | | 14085 | | | | 3954 | | | | 10131 | | | | 112% |
| Zambia | 62819 | | | | 57653 | | | | 5166 | | | | 9% | | | 8900 | | | | 7033 | | 1867 | | | | 23% | | | 5721 | | | | 5452 | | | | 269 | | | | 5% |
| Zimbabwe | 29345 | | | | 32207 | | | | -2862 | | | | -9% | | | 3314 | | | | 7238 | | -3924 | | | | -74% | | | 2263 | | | | 3881 | | | | -1618 | | | | -53% |
| EASTERN MEDITERRANEAN REGION | | | | | | | | | | | | | | | | | | | | | | | | | | | | | | | | | | | | | | | | | |
| Afghanistan | 191886 | | | | 111020 | | | | 80866 | | | | 53% | | | 48401 | | | | 22432 | | 25969 | | | | 73% | | | 30919 | | | | 13424 | | | | 17495 | | | | 79% |
| Bahrain | 215 | | | | 165 | | | | 50 | | | | 26% | | | 4 | | | | 4 | | 0 | | | | 0% | | | 0 | | | | 1 | | | | -1 | | | | -200% |
| Cyprus | 50 | | | | 32 | | | | 18 | | | | 44% | | | 2 | | | | 0.4 | | 1.6 | | | | 133% | | | 0 | | | | 0 | | | | 0 | | | | 0% |
| Djibouti | 2240 | | | | 1666 | | | | 574 | | | | 29% | | | 444 | | | | 255 | | 189 | | | | 54% | | | 243 | | | | 128 | | | | 115 | | | | 62% |
| Egypt | 40708 | | | | 48261 | | | | -7553 | | | | -17% | | | 4329 | | | | 9804 | | -5475 | | | | -77% | | | 2813 | | | | 5250 | | | | -2437 | | | | -60% |
| Iran | 34180 | | | | 33821 | | | | 359 | | | | 1% | | | 4413 | | | | 1979 | | 2434 | | | | 76% | | | 1378 | | | | 478 | | | | 900 | | | | 97% |
| Iraq | 42606 | | | | 48100 | | | | -5494 | | | | -12% | | | 7624 | | | | 8507 | | -883 | | | | -11% | | | 2503 | | | | 2559 | | | | -56 | | | | -2% |
| Jordan | 3670 | | | | 2234 | | | | 1436 | | | | 49% | | | 283 | | | | 168 | | 115 | | | | 51% | | | 146 | | | | 35 | | | | 111 | | | | 123% |
| Kuwait | 625 | | | | 647 | | | | -22 | | | | -3% | | | 39 | | | | 28 | | 11 | | | | 33% | | | 9 | | | | 4 | | | | 5 | | | | 77% |
| Lebanon | 1527 | | | | 768 | | | | 759 | | | | 66% | | | 123 | | | | 45 | | 78 | | | | 93% | | | 54 | | | | 14 | | | | 40 | | | | 118% |
| Libya | 2444 | | | | 2961 | | | | -517 | | | | -19% | | | 65 | | | | 254 | | -189 | | | | -118% | | | 19 | | | | 72 | | | | -53 | | | | -116% |
| Morocco | 22680 | | | | 22699 | | | | -19 | | | | 0% | | | 3460 | | | | 2449 | | 1011 | | | | 34% | | | 1420 | | | | 776 | | | | 644 | | | | 59% |
| Oman | 544 | | | | 563 | | | | -19 | | | | -3% | | | 28 | | | | 42 | | -14 | | | | -40% | | | 6 | | | | 5 | | | | 1 | | | | 18% |
| Pakistan | 424377 | | | | 374077 | | | | 50300 | | | | 13% | | | 79834 | | | | 65102 | | 14732 | | | | 20% | | | 46381 | | | | 52176 | | | | -5795 | | | | -12% |
| Qatar | 153 | | | | 179 | | | | -26 | | | | -16% | | | 4 | | | | 4 | | 0 | | | | 0% | | | 1 | | | | 1 | | | | 0 | | | | 0% |
| Saudi Arabia | 11541 | | | | 8657 | | | | 2884 | | | | 29% | | | 789 | | | | 244 | | 545 | | | | 106% | | | 226 | | | | 177 | | | | 49 | | | | 24% |
| Somalia | 61215 | | | | 43125 | | | | 18090 | | | | 35% | | | 15256 | | | | 6625 | | 8631 | | | | 79% | | | 9951 | | | | 5631 | | | | 4320 | | | | 55% |
| Sudan | 140731 | | | | 92671 | | | | 48060 | | | | 41% | | | 26966 | | | | 12866 | | 14100 | | | | 71% | | | 17420 | | | | 10507 | | | | 6913 | | | | 50% |
| Syria | 7788 | | | | 6804 | | | | 984 | | | | 13% | | | 627 | | | | 434 | | 193 | | | | 36% | | | 563 | | | | 58 | | | | 505 | | | | 163% |
| Tunisia | 2979 | | | | 3183 | | | | -204 | | | | -7% | | | 216 | | | | 152 | | 64 | | | | 35% | | | 92 | | | | 150 | | | | -58 | | | | -48% |
| United Arab Emirates | 639 | | | | 806 | | | | -167 | | | | -23% | | | 14 | | | | 53 | | -39 | | | | -116% | | | 2 | | | | 11 | | | | -9 | | | | -138% |
| Yemen | 68867 | | | | 53852 | | | | 15015 | | | | 24% | | | 14964 | | | | 5811 | | 9153 | | | | 88% | | | 7763 | | | | 12790 | | | | -5027 | | | | -49% |
| EUROPEAN REGION | | |  | | | |  | | | | |  | | | | |  | | | |  | | | |  | | | | |  | | | | |  | | | |  | | |
| Albania | 735 | | | | 695 | | | | 40 | | | | 6% | | | 83 | | | | 198 | | -115 | | | | -82% | | | 7 | | | | 4 | | | | 3 | | | | 55% |
| Andorra | 4 | | | | 2 | | | | 2 | | | | 67% | | | 0 | | | | 0 | | 0 | | | | 0 | | | 0 | | | | 0 | | | | 0 | | | | 0% |
| Armenia | 890 | | | | 799 | | | | 91 | | | | 11% | | | 98 | | | | 108 | | -10 | | | | -10% | | | 11 | | | | 32 | | | | -21 | | | | -98% |
| Austria | 315 | | | | 357 | | | | -42 | | | | -13% | | | 5 | | | | 2 | | 3 | | | | 86% | | | 0 | | | | 1 | | | | -1 | | | | -200% |
| Azerbaijan | 8635 | | | | 6818 | | | | 1817 | | | | 24% | | | 1497 | | | | 2162 | | -665 | | | | -36% | | | 656 | | | | 233 | | | | 423 | | | | 95% |
| Belarus | 646 | | | | 1034 | | | | -388 | | | | -46% | | | 56 | | | | 61 | | -5 | | | | -9% | | | 4 | | | | 13 | | | | -9 | | | | -106% |
| Belgium | 544 | | | | 551 | | | | -7 | | | | -1% | | | 7 | | | | 0 | | 7 | | | | 200% | | | 4 | | | | 3 | | | | 1 | | | | 29% |
| Bosnia and Herzegovina | 292 | | | | 249 | | | | 43 | | | | 16% | | | 27 | | | | 16 | | 11 | | | | 51% | | | 2 | | | | 3 | | | | -1 | | | | -40% |
| Bulgaria | 994 | | | | 840 | | | | 154 | | | | 17% | | | 243 | | | | 173 | | 70 | | | | 34% | | | 9 | | | | 11 | | | | -2 | | | | -20% |
| Croatia | 248 | | | | 238 | | | | 10 | | | | 4% | | | 8 | | | | 5 | | 3 | | | | 46% | | | 0 | | | | 1 | | | | -1 | | | | -200% |
| Czech Republic | 470 | | | | 406 | | | | 64 | | | | 15% | | | 24 | | | | 10 | | 14 | | | | 82% | | | 7 | | | | 1 | | | | 6 | | | | 150% |
| Denmark | 258 | | | | 249 | | | | 9 | | | | 4% | | | 6 | | | | 0 | | 6 | | | | 200% | | | 1 | | | | 1 | | | | 0 | | | | 0% |
| Estonia | 76 | | | | 95 | | | | -19 | | | | -22% | | | 2 | | | | 4 | | -2 | | | | -67% | | | 0 | | | | 0 | | | | 0 | | | | 0% |
| Finland | 184 | | | | 182 | | | | 2 | | | | 1% | | | 7 | | | | 0 | | 7 | | | | 200% | | | 0 | | | | 1 | | | | -1 | | | | -200% |
| France | 3282 | | | | 3312 | | | | -30 | | | | -1% | | | 61 | | | | 0 | | 61 | | | | 200% | | | 29 | | | | 27 | | | | 2 | | | | 7% |
| Georgia | 1214 | | | | 1348 | | | | -134 | | | | -10% | | | 131 | | | | 188 | | -57 | | | | -36% | | | 15 | | | | 17 | | | | -2 | | | | -13% |
| Germany | 2920 | | | | 2822 | | | | 98 | | | | 3% | | | 66 | | | | 0 | | 66 | | | | 200% | | | 11 | | | | 11 | | | | 0 | | | | 0% |
| Greece | 505 | | | | 439 | | | | 66 | | | | 14% | | | 37 | | | | 9 | | 28 | | | | 122% | | | 0 | | | | 1 | | | | -1 | | | | -200% |
| Hungary | 635 | | | | 562 | | | | 73 | | | | 12% | | | 28 | | | | 19 | | 9 | | | | 38% | | | 1 | | | | 1 | | | | 0 | | | | 0% |
| Iceland | 11 | | | | 14 | | | | -3 | | | | -24% | | | 0 | | | | 0 | | 0 | | | | 0% | | | 0 | | | | 0 | | | | 0 | | | | 0% |
| Ireland | 301 | | | | 322 | | | | -21 | | | | -7% | | | 4 | | | | 2 | | 2 | | | | 67% | | | 1 | | | | 1 | | | | 0 | | | | 0% |
| Israel | 689 | | | | 738 | | | | -49 | | | | -7% | | | 13 | | | | 11 | | 2 | | | | 17% | | | 3 | | | | 4 | | | | -1 | | | | -29% |
| Italy | 2157 | | | | 2381 | | | | -224 | | | | -10% | | | 30 | | | | 19 | | 11 | | | | 45% | | | 0 | | | | 4 | | | | -4 | | | | -200% |
| Kazakhstan | 12686 | | | | 11415 | | | | 1271 | | | | 11% | | | 1678 | | | | 2086 | | -408 | | | | -22% | | | 719 | | | | 315 | | | | 404 | | | | 78% |
| Kyrgyzstan | 5256 | | | | 6062 | | | | -806 | | | | -14% | | | 753 | | | | 1775 | | -1022 | | | | -81% | | | 335 | | | | 383 | | | | -48 | | | | -13% |
| Latvia | 227 | | | | 178 | | | | 49 | | | | 24% | | | 21 | | | | 7 | | 14 | | | | 100% | | | 0 | | | | 0 | | | | 0 | | | | 0% |
| Lithuania | 239 | | | | 199 | | | | 40 | | | | 18% | | | 22 | | | | 8 | | 14 | | | | 93% | | | 1 | | | | 0 | | | | 1 | | | | 200% |
| Luxembourg | 18 | | | | 19 | | | | -1 | | | | -5% | | | 0 | | | | 0.2 | | -0.2 | | | | -200% | | | 0 | | | | 0 | | | | 0 | | | | 0% |
| Macedonia | 267 | | | | 240 | | | | 27 | | | | 11% | | | 12 | | | | 17 | | -5 | | | | -34% | | | 8 | | | | 13 | | | | -5 | | | | -48% |
| Malta | 25 | | | | 28 | | | | -3 | | | | -11% | | | 0 | | | | 1 | | -1 | | | | -200% | | | 0 | | | | 0 | | | | 0 | | | | 0% |
| Moldova | 932 | | | | 628 | | | | 304 | | | | 39% | | | 198 | | | | 110 | | 88 | | | | 57% | | | 3 | | | | 10 | | | | -7 | | | | -108% |
| Montenegro | 63 | | | | 57 | | | | 6 | | | | 10% | | | 0 | | | | 3 | | -3 | | | | -200% | | | 0 | | | | 0 | | | | 0 | | | | 0% |
| Netherlands | 750 | | | | 828 | | | | -78 | | | | -10% | | | 20 | | | | 0 | | 20 | | | | 200% | | | 1 | | | | 3 | | | | -2 | | | | -100% |
| Norway | 214 | | | | 203 | | | | 11 | | | | 5% | | | 3 | | | | 0 | | 3 | | | | 200% | | | 0 | | | | 1 | | | | -1 | | | | -200% |
| Poland | 2547 | | | | 2409 | | | | 138 | | | | 6% | | | 131 | | | | 80 | | 51 | | | | 48% | | | 7 | | | | 8 | | | | -1 | | | | -13% |
| Portugal | 360 | | | | 354 | | | | 6 | | | | 2% | | | 4 | | | | 7 | | -3 | | | | -55% | | | 0 | | | | 3 | | | | -3 | | | | -200% |
| Romania | 3052 | | | | 2507 | | | | 545 | | | | 20% | | | 899 | | | | 874 | | 25 | | | | 3% | | | 4 | | | | 45 | | | | -41 | | | | -167% |
| Russia | 19574 | | | | 21246 | | | | -1672 | | | | -8% | | | 1624 | | | | 1979 | | -355 | | | | -20% | | | 147 | | | | 411 | | | | -264 | | | | -95% |
| Serbia | 760 | | | | 543 | | | | 217 | | | | 33% | | | 30 | | | | 30 | | 0 | | | | 0% | | | 0 | | | | 3 | | | | -3 | | | | -200% |
| Slovakia | 474 | | | | 413 | | | | 61 | | | | 14% | | | 39 | | | | 34 | | 5 | | | | 14% | | | 2 | | | | 3 | | | | -1 | | | | -40% |
| Slovenia | 61 | | | | 70 | | | | -9 | | | | -14% | | | 2 | | | | 1 | | 1 | | | | 67% | | | 2 | | | | 0 | | | | 2 | | | | 200% |
| Spain | 2447 | | | | 2030 | | | | 417 | | | | 19% | | | 61 | | | | 18 | | 43 | | | | 109% | | | 17 | | | | 8 | | | | 9 | | | | 72% |
| Sweden | 336 | | | | 361 | | | | -25 | | | | -7% | | | 11 | | | | 0 | | 11 | | | | 200% | | | 0 | | | | 2 | | | | -2 | | | | -200% |
| Switzerland | 360 | | | | 353 | | | | 7 | | | | 2% | | | 3 | | | | 3 | | 0 | | | | 0% | | | 0 | | | | 5 | | | | -5 | | | | -200% |
| Tajikistan | 11758 | | | | 9716 | | | | 2042 | | | | 19% | | | 2020 | | | | 2796 | | -776 | | | | -32% | | | 1065 | | | | 1200 | | | | -135 | | | | -12% |
| Turkey | 23658 | | | | 33330 | | | | -9672 | | | | -34% | | | 2590 | | | | 1986 | | 604 | | | | 26% | | | 265 | | | | 402 | | | | -137 | | | | -41% |
| Turkmenistan | 5683 | | | | 2650 | | | | 3033 | | | | 73% | | | 881 | | | | 710 | | 171 | | | | 21% | | | 439 | | | | 328 | | | | 111 | | | | 29% |
| Ukraine | 6947 | | | | 6891 | | | | 56 | | | | 1% | | | 841 | | | | 416 | | 425 | | | | 68% | | | 138 | | | | 141 | | | | -3 | | | | -2% |
| United Kingdom | 4327 | | | | 4078 | | | | 249 | | | | 6% | | | 176 | | | | 72 | | 104 | | | | 84% | | | 3 | | | | 14 | | | | -11 | | | | -129% |
| Uzbekistan | 31476 | | | | 24307 | | | | 7169 | | | | 26% | | | 4732 | | | | 10363 | | -5631 | | | | -75% | | | 2360 | | | | 393 | | | | 1967 | | | | 143% |
| REGION OF THE AMERICAS | |  | | | |  | | | | |  | | | |  | | | |  | | | |  | | | |  | | | |  | | | | |  | | | |  | |
| Antigua and Barbuda | 11 | | | | 22 | | | | -11 | | | | -67% | | | 0 | | | | 1 | | -1 | | | | -200% | | | 0 | | | | 0 | | | | 0 | | | | 0% |
| Argentina | 9761 | | | | 10323 | | | | -562 | | | | -6% | | | 929 | | | | 711 | | 218 | | | | 27% | | | 169 | | | | 159 | | | | 10 | | | | 6% |
| Bahamas | 93 | | | | 106 | | | | -13 | | | | -13% | | | 25 | | | | 3 | | 22 | | | | 157% | | | 0 | | | | 0 | | | | 0 | | | | 0% |
| Barbados | 65 | | | | 61 | | | | 4 | | | | 6% | | | 4 | | | | 3 | | 1 | | | | 29% | | | 0 | | | | 0 | | | | 0 | | | | 0% |
| Belize | 126 | | | | 159 | | | | -33 | | | | -23% | | | 9 | | | | 17 | | -8 | | | | -62% | | | 13 | | | | 7 | | | | 6 | | | | 60% |
| Bolivia | 13750 | | | | 13939 | | | | -189 | | | | -1% | | | 2064 | | | | 2666 | | -602 | | | | -25% | | | 1216 | | | | 2091 | | | | -875 | | | | -53% |
| Brazil | 54815 | | | | 62296 | | | | -7481 | | | | -13% | | | 3910 | | | | 5054 | | -1144 | | | | -26% | | | 1776 | | | | 2809 | | | | -1033 | | | | -45% |
| Canada | 2353 | | | | 2133 | | | | 220 | | | | 10% | | | 30 | | | | 42 | | -12 | | | | -33% | | | 1 | | | | 7 | | | | -6 | | | | -150% |
| Chile | 2179 | | | | 2243 | | | | -64 | | | | -3% | | | 148 | | | | 180 | | -32 | | | | -20% | | | 14 | | | | 28 | | | | -14 | | | | -67% |
| Colombia | 17640 | | | | 20286 | | | | -2646 | | | | -14% | | | 1679 | | | | 2468 | | -789 | | | | -38% | | | 637 | | | | 1071 | | | | -434 | | | | -51% |
| Costa Rica | 713 | | | | 750 | | | | -37 | | | | -5% | | | 25 | | | | 58 | | -33 | | | | -80% | | | 10 | | | | 15 | | | | -5 | | | | -40% |
| Cuba | 617 | | | | 724 | | | | -107 | | | | -16% | | | 66 | | | | 67 | | -1 | | | | -2% | | | 12 | | | | 9 | | | | 3 | | | | 29% |
| Dominica | 13 | | | | 30 | | | | -17 | | | | -79% | | | 0 | | | | 2 | | -2 | | | | -200% | | | 0 | | | | 0 | | | | 0 | | | | 0% |
| Dominican Republic | 5676 | | | | 7238 | | | | -1562 | | | | -24% | | | 629 | | | | 1775 | | -1146 | | | | -95% | | | 240 | | | | 351 | | | | -111 | | | | -38% |
| Ecuador | 6014 | | | | 6707 | | | | -693 | | | | -11% | | | 600 | | | | 1353 | | -753 | | | | -77% | | | 230 | | | | 366 | | | | -136 | | | | -46% |
| El Salvador | 2186 | | | | 1920 | | | | 266 | | | | 13% | | | 243 | | | | 215 | | 28 | | | | 12% | | | 117 | | | | 147 | | | | -30 | | | | -23% |
| Grenada | 23 | | | | 30 | | | | -7 | | | | -26% | | | 0 | | | | 2 | | -2 | | | | -200% | | | 0 | | | | 0 | | | | 0 | | | | 0% |
| Guatemala | 14340 | | | | 14230 | | | | 110 | | | | 1% | | | 2122 | | | | 3534 | | -1412 | | | | -50% | | | 995 | | | | 2231 | | | | -1236 | | | | -77% |
| Guyana | 343 | | | | 468 | | | | -125 | | | | -31% | | | 14 | | | | 38 | | -24 | | | | -92% | | | 10 | | | | 54 | | | | -44 | | | | -138% |
| Haiti | 45852 | | | | 33094 | | | | 12758 | | | | 32% | | | 4678 | | | | 2239 | | 2439 | | | | 71% | | | 3265 | | | | 2395 | | | | 870 | | | | 31% |
| Honduras | 4843 | | | | 4829 | | | | 14 | | | | 0% | | | 539 | | | | 397 | | 142 | | | | 30% | | | 265 | | | | 670 | | | | -405 | | | | -87% |
| Jamaica | 1313 | | | | 1072 | | | | 241 | | | | 20% | | | 171 | | | | 64 | | 107 | | | | 91% | | | 57 | | | | 74 | | | | -17 | | | | -26% |
| Mexico | 36584 | | | | 37210 | | | | -626 | | | | -2% | | | 4459 | | | | 4281 | | 178 | | | | 4% | | | 1438 | | | | 1731 | | | | -293 | | | | -18% |
| Nicaragua | 3855 | | | | 3695 | | | | 160 | | | | 4% | | | 548 | | | | 589 | | -41 | | | | -7% | | | 342 | | | | 365 | | | | -23 | | | | -7% |
| Panama | 1411 | | | | 1262 | | | | 149 | | | | 11% | | | 132 | | | | 135 | | -3 | | | | -2% | | | 161 | | | | 77 | | | | 84 | | | | 71% |
| Paraguay | 3742 | | | | 3589 | | | | 153 | | | | 4% | | | 406 | | | | 319 | | 87 | | | | 24% | | | 184 | | | | 207 | | | | -23 | | | | -12% |
| Peru | 11384 | | | | 15172 | | | | -3788 | | | | -29% | | | 1128 | | | | 2355 | | -1227 | | | | -70% | | | 429 | | | | 462 | | | | -33 | | | | -7% |
| Saint Lucia | 52 | | | | 63 | | | | -11 | | | | -19% | | | 0 | | | | 3 | | -3 | | | | -200% | | | 1 | | | | 1 | | | | 0 | | | | 0% |
| Saint Vincent and the Grenadines | 41 | | | | 55 | | | | -14 | | | | -29% | | | 1 | | | | 3 | | -2 | | | | -100% | | | 0 | | | | 1 | | | | -1 | | | | -200% |
| Suriname | 285 | | | | 319 | | | | -34 | | | | -11% | | | 25 | | | | 22 | | 3 | | | | 13% | | | 9 | | | | 9 | | | | 0 | | | | 0% |
| Trinidad and Tobago | 542 | | | | 432 | | | | 110 | | | | 23% | | | 37 | | | | 24 | | 13 | | | | 43% | | | 0 | | | | 7 | | | | -7 | | | | -200% |
| United States | 32270 | | | | 30838 | | | | 1432 | | | | 5% | | | 805 | | | | 539 | | 266 | | | | 40% | | | 68 | | | | 95 | | | | -27 | | | | -33% |
| Uruguay | 537 | | | | 483 | | | | 54 | | | | 11% | | | 57 | | | | 36 | | 21 | | | | 45% | | | 11 | | | | 12 | | | | -1 | | | | -9% |
| Venezuela | 10936 | | | | 9454 | | | | 1482 | | | | 15% | | | 1143 | | | | 860 | | 283 | | | | 28% | | | 802 | | | | 807 | | | | -5 | | | | -1% |
| SOUTH-EAST ASIA REGION | | |  | | | |  | | | | |  | | | | |  | | | |  | | | |  | | | | |  | | | | |  | | | |  | | |
| Bangladesh | 139740 | | | | 151703 | | | | -11963 | | | | -8% | | | 19209 | | | | 13879 | | 5330 | | | | 32% | | | 8229 | | | | 9758 | | | | -1529 | | | | -17% |
| Bhutan | 811 | | | | 784 | | | | 27 | | | | 3% | | | 161 | | | | 103 | | 58 | | | | 44% | | | 59 | | | | 45 | | | | 14 | | | | 27% |
| India | 1682157 | | | | 1575169 | | | | 106988 | | | | 7% | | | 396723 | | | | 198645 | | 198078 | | | | 67% | | | 212229 | | | | 150622 | | | | 61607 | | | | 34% |
| Indonesia | 151650 | | | | 147472 | | | | 4178 | | | | 3% | | | 21872 | | | | 13871 | | 8001 | | | | 45% | | | 7824 | | | | 25798 | | | | -17974 | | | | -107% |
| Maldives | 80 | | | | 87 | | | | -7 | | | | -8% | | | 9 | | | | 4 | | 5 | | | | 77% | | | 3 | | | | 1 | | | | 2 | | | | 100% |
| Myanmar | 56018 | | | | 41809 | | | | 14209 | | | | 29% | | | 9739 | | | | 5695 | | 4044 | | | | 52% | | | 4211 | | | | 8914 | | | | -4703 | | | | -72% |
| Nepal | 34526 | | | | 36852 | | | | -2326 | | | | -7% | | | 5670 | | | | 7635 | | -1965 | | | | -30% | | | 2190 | | | | 6266 | | | | -4076 | | | | -96% |
| North Korea | 11735 | | | | 8916 | | | | 2819 | | | | 27% | | | 1734 | | | | 1360 | | 374 | | | | 24% | | | 640 | | | | 180 | | | | 460 | | | | 112% |
| Sri Lanka | 6336 | | | | 3228 | | | | 3108 | | | | 65% | | | 426 | | | | 167 | | 259 | | | | 87% | | | 217 | | | | 66 | | | | 151 | | | | 107% |
| Thailand | 10899 | | | | 11051 | | | | -152 | | | | -1% | | | 984 | | | | 1143 | | -159 | | | | -15% | | | 367 | | | | 176 | | | | 191 | | | | 70% |
| Timor-Leste | 2449 | | | | 2306 | | | | 143 | | | | 6% | | | 493 | | | | 426 | | 67 | | | | 15% | | | 188 | | | | 283 | | | | -95 | | | | -40% |
| WESTERN PACIFIC REGION | | | |  | | | |  | | | | | |  | | | |  | | | | | |  | | | |  | | | | | |  | | | |  | | | |
| Australia | 1498 | | | | 1468 | | | | 30 | | | | 2% | | | 40 | | | | 22 | | 18 | | | | 58% | | | 7 | | | | 3 | | | | 4 | | | | 80% |
| Brunei Darussalam | 54 | | | | 54 | | | | 0 | | | | 0% | | | 3 | | | | 3 | | 0 | | | | 0% | | | 0 | | | | 1 | | | | -1 | | | | -200% |
| Cambodia | 16324 | | | | 15348 | | | | 976 | | | | 6% | | | 2669 | | | | 2731 | | -62 | | | | -2% | | | 1315 | | | | 1155 | | | | 160 | | | | 13% |
| China | 314581 | | | | 212987 | | | | 101594 | | | | 39% | | | 54681 | | | | 26864 | | 27817 | | | | 68% | | | 9786 | | | | 1946 | | | | 7840 | | | | 134% |
| Fiji | 331 | | | | 559 | | | | -228 | | | | -51% | | | 33 | | | | 81 | | -48 | | | | -84% | | | 14 | | | | 25 | | | | -11 | | | | -56% |
| Japan | 3469 | | | | 3389 | | | | 80 | | | | 2% | | | 206 | | | | 0 | | 206 | | | | 200% | | | 63 | | | | 29 | | | | 34 | | | | 74% |
| Kiribati | 98 | | | | 100 | | | | -2 | | | | -2% | | | 20 | | | | 10 | | 10 | | | | 67% | | | 8 | | | | 5 | | | | 3 | | | | 46% |
| Laos | 7677 | | | | 9772 | | | | -2095 | | | | -24% | | | 1461 | | | | 1750 | | -289 | | | | -18% | | | 774 | | | | 1111 | | | | -337 | | | | -36% |
| Malaysia | 3454 | | | | 4354 | | | | -900 | | | | -23% | | | 191 | | | | 327 | | -136 | | | | -53% | | | 55 | | | | 28 | | | | 27 | | | | 65% |
| Marshall Islands | 29 | | | | 65 | | | | -36 | | | | -77% | | | 6 | | | | 6 | | 0 | | | | 0% | | | 2 | | | | 3 | | | | -1 | | | | -40% |
| Micronesia | 116 | | | | 40 | | | | 76 | | | | 97% | | | 24 | | | | 3 | | 21 | | | | 156% | | | 7 | | | | 1 | | | | 6 | | | | 150% |
| Mongolia | 2251 | | | | 2471 | | | | -220 | | | | -9% | | | 346 | | | | 806 | | -460 | | | | -80% | | | 163 | | | | 19 | | | | 144 | | | | 158% |
| New Zealand | 396 | | | | 388 | | | | 8 | | | | 2% | | | 31 | | | | 12 | | 19 | | | | 88% | | | 1 | | | | 1 | | | | 0 | | | | 0% |
| Papua New Guinea | 12469 | | | | 10573 | | | | 1896 | | | | 16% | | | 2135 | | | | 2191 | | -56 | | | | -3% | | | 1031 | | | | 1116 | | | | -85 | | | | -8% |
| Philippines | 66097 | | | | 70021 | | | | -3924 | | | | -6% | | | 10673 | | | | 14571 | | -3898 | | | | -31% | | | 3843 | | | | 3730 | | | | 113 | | | | 3% |
| Samoa | 87 | | | | 80 | | | | 7 | | | | 8% | | | 7 | | | | 5 | | 2 | | | | 33% | | | 3 | | | | 1 | | | | 2 | | | | 100% |
| Singapore | 126 | | | | 94 | | | | 32 | | | | 29% | | | 9 | | | | 6 | | 3 | | | | 40% | | | 1 | | | | 1 | | | | 0 | | | | 0% |
| Solomon Islands | 450 | | | | 443 | | | | 7 | | | | 2% | | | 74 | | | | 45 | | 29 | | | | 49% | | | 21 | | | | 25 | | | | -4 | | | | -17% |
| South Korea | 2659 | | | | 1861 | | | | 798 | | | | 35% | | | 56 | | | | 51 | | 5 | | | | 9% | | | 11 | | | | 11 | | | | 0 | | | | 0% |
| Tonga | 44 | | | | 58 | | | | -14 | | | | -27% | | | 4 | | | | 3 | | 1 | | | | 29% | | | 1 | | | | 2 | | | | -1 | | | | -67% |
| Vanuatu | 96 | | | | 217 | | | | -121 | | | | -77% | | | 9 | | | | 24 | | -15 | | | | -91% | | | 3 | | | | 14 | | | | -11 | | | | -129% |
| Viet Nam | 34940 | | | | 24134 | | | | 10806 | | | | 37% | | | 4122 | | | | 3139 | | 983 | | | | 27% | | | 3585 | | | | 470 | | | | 3115 | | | | 154% |

* Percent difference calculated: (CHERG - GBD 2010) / ((CHERG + GBD 2010) / 2)

** LRI: Lower respiratory infection
