# Supplementary material for: Deconstructing the differences: a comparison of GBD 2010 and CHERG’s approach to estimating the mortality burden of diarrhea, pneumonia, and their etiologies
Source: BMC Infect Dis. 2015 Jan 16;15:16. doi: 10.1186/s12879-014-0728-4 (PMC4305232; doi:10.1186/s12879-014-0728-4)
Supplement: Supplementary file 2 — Data sources for pneumonia etiology mortality models for GBD 2010 and CHERG. [file 12879_2014_728_MOESM2_ESM.docx]

**Additional file 2:**

**Data sources for pneumonia etiology mortality models for GBD 2010 and CHERG**

| **Source Citation** | **CHERG [9]** | | **GBD 2010 [6]** | | | | |
| --- | --- | --- | --- | --- | --- | --- | --- |
|  | ***Pneumococcus*** | **Hib** | **RSV** | ***Pneumococcus*** | **Hib** | **Influenza** | **Other LRI** |
| Adegbola RA, Falade AG, Sam BE, Aidoo M, Baldeh I, Hazlett D, Whittle H, Greenwood BM, Mulholland EK. The etiology of pneumonia in malnourished and well-nourished Gambian children. The Pediatric infectious disease journal 1994; 13(11): 975–82. |  |  | x | x | x | x | x |
| Almirall J, Boixeda R, Bol’bar I, Bassa J, Sauca G, Vidal J, Serra-Prat M, Balanz— X, GEMPAC Study Group. Differences in the etiology of community-acquired pneumonia according to site of care: a population-based study. Respiratory medicine 2007; 101(10): 2168–75. |  |  | x | x | x | x | x |
| Baqui AH, El Arifeen S, Saha SK, Persson L, Zaman K, Gessner BD, Moulton LH, Black RE, Santosham M. Effectiveness of Haemophilus influenzae type B conjugate vaccine on prevention of pneumonia and meningitis in Bangladeshi children: a case-control study. The Pediatric infectious disease journal 2007; 26(7): 565–71. |  | x |  |  | x |  |  |
| Black SB, Shinefield HR, Ling S, Hansen J, Fireman B, Spring D, Noyes J, Lewis E, Ray P, Lee J, Hackell J. Effectiveness of heptavalent pneumococcal conjugate vaccine in children younger than five years of age for prevention of pneumonia. The Pediatric infectious disease journal 2002; 21(9): 810–5. | x |  |  | x |  |  |  |
| Blanquer J, Sanz F. Neumonía adquirida en la comunidad. Archivos de bronconeumología 2010; 46: 26–30. |  |  |  | x | x |  |  |
| Briones ML, Blanquer J, Ferrando D, Blasco ML, Gimeno C, Mar’n J. Assessment of analysis of urinary pneumococcal antigen by immunochromatography for etiologic diagnosis of community-acquired pneumonia in adults. Clinical and vaccine immunology?: CVI 2006; 13(10): 1092–7. |  |  | x | x | x | x | x |
| Cao B, Song S.-F, Bai L, Yin Y.D, Zhang Y.-Y, Liu Y.-M, Guo P, Wang C, Ren L.-L, Wang J.-W, Zhao F, Zhang J.-Z, Gonzalez R. Viral and Mycoplasma pneumoniae community-acquired pneumonia and novel clinical outcome evaluation in ambulatory adult patients in China. Eur. J. Clin. Microbiol. Infect. Dis. 2010; 29(11): 1443–8. |  |  | x | x |  | x |  |
| Capeding MRZ, Sombrero LT, Paladin FJ, Suzuki H. Etiology of acute lower respiratory infection in Filipino children under five years. The Southeast Asian journal of tropical medicine and public health 1994; 25(4): 684. |  |  | x | x | x | x | x |
| Carratalà J, Mykietiuk A, Fernández-Sabé N, Suárez C, Dorca J, Verdaguer R, Manresa F, Gudiol F. Health care-associated pneumonia requiring hospital admission: epidemiology, antibiotic therapy, and clinical outcomes. Archives of internal medicine 2007; 167(13): 1393–9. |  |  |  | x | x |  |  |
| Cevey-Macherel M, Galetto-Lacour A, Gervaix A, Siegrist C-A, Bille J, Bescher-Ninet B, Kaiser L, Krahenbuhl J-D, Gehri M. Etiology of community-acquired pneumonia in hospitalized children based on WHO clinical guidelines. European Journal of Pediatrics 2009; 168(12): 1429–36. |  |  | x | x |  | x |  |
| Charles P.G.P, Grayson M.L, Pierce R.J.P, Mayall B.C, Fuller A.J, Stirling R, Hooy M, Korman T.M, Holmes P.W, Wright A.A, Catton M.G, Whitby M, Johnson B, Armstrong J.G, Nimmo G.R, Christiansen K.J, Waterer G.W, Grayson L, Johnson P, Munckhof W. The etiology of community-acquired pneumonia in Australia: Why penicillin plus doxycycline or a macrolide is the most appropriate therapy. Clin. Infect. Dis. 2008; 46(10): 1513–21. |  |  | x | x | x | x | x |
| Chen R, Hao CL, Zhao GM, Zhang T, Lin YZ, Ding YF, Tao YZ, Zhu LJ. Zhongguo dang dai er ke za zhi = Chinese journal of contemporary pediatrics. Chinese journal of contemporary pediatrics 2008; 10(2): 143–5. |  |  | x | x | x |  |  |
| Cutts F., Zaman SM., Enwere G, Jaffar S, Levine O., Okoko J., Oluwalana C, Vaughan A, Obaro S., Leach A, McAdam K., Biney E, Saaka M, Onwuchekwa U, Yallop F, Pierce N., Greenwood B., Adegbola R. Efficacy of nine-valent pneumococcal conjugate vaccine against pneumonia and invasive pneumococcal disease in The Gambia: randomised, double-blind, placebo-controlled trial. Lancet 2005; 365(9465): 28. | x |  |  | x |  |  |  |
| De Schutter I, De Wachter E, Malfroot A, Soetens O, Pierard D, Crokaert F, Verhaegen J. Microbiology of bronchoalveolar lavage fluid in children with acute nonresponding or recurrent community-acquired pneumonia: Identification of nontypeable haemophilus influenzae as a major pathogen. Clin. Infect. Dis. 2011; 52(12): 1437–44. |  |  | x | x | x | x | x |
| Díaz A, Barria P, Niederman M, Restrepo MI, Dreyse J, Fuentes G, Couble B, Saldias F. Etiology of community-acquired pneumonia in hospitalized patients in chile: the increasing prevalence of respiratory viruses among classic pathogens. Chest 2007; 131(3): 779–87. |  |  | x | x | x | x | x |
| Don M, Valerio G, Korppi M, Canciani M. Hyper- and hypoglycemia in children with community-acquired pneumonia. Journal of Pediatric Endocrinology & Metabolism 2008; 21(7): 657–64. |  |  | x | x |  |  |  |
| Don M, Valerio G, Korppi M, Canciani M. Hyponatremia in pediatric community-acquired pneumonia. Pediatric Nephrology 2008; 23(12): 2247–53. |  |  |  | x |  |  |  |
| Forgie IM, Campbell H, Lloyd-Evans N, Leinonen M, O’Neill KP, Saikku P, Whittle HC, Greenwood BM. Etiology of acute lower respiratory tract infections in children in a rural community in The Gambia. The Pediatric infectious disease journal 1992; 11(6): 466–73. |  |  | x | x | x | x | x |
| Forgie IM, O’Neill KP, Lloyd-Evans N, Leinonen M, Campbell H, Whittle HC, Greenwood BM. Etiology of acute lower respiratory tract infections in Gambian children: I. Acute lower respiratory tract infections in infants presenting at the hospital. The Pediatric infectious disease journal 1991; 10(1): 33–41. |  |  | x | x | x | x | x |
| Forgie IM, O’Neill KP, Lloyd-Evans N, Leinonen M, Campbell H, Whittle HC, Greenwood BM. Etiology of acute lower respiratory tract infections in Gambian children: II. Acute lower respiratory tract infection in children ages one to nine years presenting at the hospital. The Pediatric infectious disease journal 1991; 10(1): 42–7. |  |  | x | x | x | x | x |
| Gessner BD, Sutanto A, Linehan M, Djelantik IG, Fletcher T, Gerudug IK, Ingerani, Mercer D, Moniaga V, Moulton LH, Moulton LH, Mulholland K, Nelson C, Soemohardjo S, Steinhoff M, Widjaya A, Stoeckel P, Maynard J, Arjoso S. Incidences of vaccine-preventable Haemophilus influenzae type b pneumonia and meningitis in Indonesian children: hamlet-randomised vaccine-probe trial. Lancet 2005; 365(9453): 43–52. |  | x |  |  | x |  |  |
| Hamano-Hasegawa K, Morozumi M, Nakayama E, Chiba N, Murayama S, Takayanagi R, Iwata S, Sunakawa K, Ubukata K. Comprehensive detection of causative pathogens using real-time PCR to diagnose pediatric community-acquired pneumonia. Journal of Infection and Chemotherapy 2008; 14(6): 424–32. |  |  | x | x | x | x | x |
| Hansen J, Black S, Shinefield H, et al. Effectiveness of heptavalent pneumococcal conjugate vaccine in children younger than 5 years of age for prevention of pneumonia: updated analysis using World Health Organization standardized interpretation of chest radiographs. Pediatr Infect Dis J 2006; 25: 779–81. | x |  |  |  |  |  |  |
| Hartung T., Chimbayo D, van Oosterhout JJ., Chikaonda T, van Doornum GJ., Claas EC., Melchers WJ., Molyneux M., Zijlstra E. Etiology of suspected pneumonia in adults admitted to a high-dependency unit in Blantyre, Malawi. AMERICAN JOURNAL OF TROPICAL MEDICINE AND HYGIENE 2011; 85(1): 105–12. |  |  |  | x |  |  |  |
| Hasan K, Jolly P, Marquis G, Roy E, Podder G, Alam K, Huq F, Sack R. Viral etiology of pneumonia in a cohort of newborns till 24 months of age in Rural Mirzapur, Bangladesh. Scandinavian Journal of Infectious Diseases 2006; 38(8): 690–5. |  |  | x | x |  | x |  |
| Hijazi Z, Pacsa A, El-Gharbawy F, Chugh TD, Essa S, El Shazli A, Abd El-Salam R. Acute lower respiratory tract infections in children in Kuwait. ANNALS OF TROPICAL PAEDIATRICS 1997; 17(2): 127–34. |  |  | x |  |  | x |  |
| Hortal M, Mogdasy C, Russi JC, Deleon C, Suarez A. Microbial agents associated with pneumonia in children from Uruguay. Reviews of infectious diseases 1990; 12 suppl 8: 915–22. |  |  | x | x | x | x | x |
| Johansson N, Kalin M, Hedlund J, Annika T.-L, Giske C.G. Etiology of Community-Acquired pneumonia: Increased microbiological yield with new diagnostic methods. Clin. Infect. Dis. 2010; 50(2): 202–9. |  |  |  | x | x |  |  |
| John TJ, Cherian T, Steinhoff MC, Simoes EA, John M. Etiology of acute respiratory infections in children in tropical southern India. Reviews of infectious diseases 1991; 13 Suppl 6: S463–9. |  |  | x | x | x | x | x |
| Klugman KP, Madhi SA, Huebner RE, Kohberger R, Mbelle N, Pierce N, Vaccine Trialists Group. A trial of a 9-valent pneumococcal conjugate vaccine in children with and those without HIV infection. The New England journal of medicine 2003; 349(14): 1341–8. | x |  |  | x |  |  |  |
| Koksal I, Bayraktar O, Yilmaz G, Caylan R, Aydin K, Sucu N, Ozlu T, Bulbul Y, Oztuna F, Ozcan D.I, OguS C, Tekeli E, Kaya A, Ayaz C, Dagli C.E, Yildiz O, Oymak F.S, Kalkan A, Muz M.H, Turgut H, Fisekci F, Heper Y, Uzaslan E. Etiological agents of community-acquired pneumonia in adult patients in Turkey; a multicentric, cross-sectional study. Tuberk 2010; 58(2): 119–27. |  |  | x | x | x | x | x |
| Levine OS, Lagos R, Muñoz A, Villaroel J, Alvarez AM, Abrego P, Levine MM. Defining the burden of pneumonia in children preventable by vaccination against Haemophilus influenzae type b. The Pediatric infectious disease journal 1999; 18(12): 1060–4. |  | x |  |  | x |  |  |
| Liu YN, Chen MJ, Zhao TM, Wang H, Wang R, Liu QF, Cai BQ, Cao B, Sun TY, Hu YJ, Xiu QY, Zhou X, Ding X, Yang L, Zhuo JS, Tang YC, Zhang KX, Liang DR, Lü XJ, Li SQ, Liu Y, Yu YS, Wei ZQ, Ying KJ, Zhao F, Chen P, Hou XN. A multicentre study on the pathogenic agents in 665 adult patients with community-acquired pneumonia in cities of China. Chinese journal of tuberculosis and respiratory diseases 2006; 29(1): 3–8. |  |  |  | x | x |  |  |
| Lucero MG, Nohynek H, Williams G, Tallo V, Simões EA, Lupisan S, Sanvictores D, Forsyth S, Puumalainen T, Ugpo J, Lechago M, de Campo M, Abucejo-Ladesma E, Sombrero L, Nissinen A, Soininen A, Ruutu P, Riley I, Mäkelä HP. Efficacy of an 11-valent pneumococcal conjugate vaccine against radiologically confirmed pneumonia among children less than 2 years of age in the Philippines: A randomized, double-blind, placebo-controlled trial. Pediatr. Infect. Dis. J. 2009; 28(6): 455–62. | x |  |  | x |  |  |  |
| Maruyama T, Gabazza E., Morser J, Takagi T, D’Alessandro-Gabazza C, Hirohata S, Nakayama S, Ramirez A., Fujiwara A, Naito M, Nishikubo K, Yuda H, Yoshida M, Takei Y, Taguchi O. Community-acquired pneumonia and nursing home-acquired pneumonia in the very elderly patients. Respir. Med. 2010; 104(4): 584–92. |  |  | x | x | x | x | x |
| Maruyama T, Niederman MS, Kobayashi T, Kobayashi H, Takagi T, D'Alessandro-Gabazza CN, Fujimoto H, Gil Bernabe P, Hirohata S, Nakayama S, Nishikubo K, Yuda H, Yamaguchi A, Gabazza EC, Noguchi T, Takei Y, Taguchi O. A prospective comparison of nursing home-acquired pneumonia with hospital-acquired pneumonia in non-intubated elderly. Respir. Med. 2008; 102(9): 1287–95. |  |  | x | x | x | x | x |
| Meijvis S.C.A, Biesma D.H, Voorn G.P, Van De Garde E.M.W, Cornips M.C.A, Souverein P.C, Leufkens H.G.M, Endeman H. Microbial evaluation of proton-pump inhibitors and the risk of pneumonia. Eur. Respir. J. 2011; 38(5): 1165–72. |  |  |  | x |  |  |  |
| Moreno L, Krishnan JA, Duran P, Ferrero F. Development and validation of a clinical prediction rule to distinguish bacterial from viral pneumonia in children. Pediatric pulmonology 2006; 41(4): 331–7. |  |  | x | x | x | x | x |
| Mulholland K, Hilton S, Adegbola R, Usen S, Oparaugo A, Omosigho C, Weber M, Palmer A, Schneider G, Jobe K, Lahai G, Jaffar S, Secka O, Lin K, Ethevenaux C, Greenwood B. Randomised trial of Haemophilus influenzae type-b tetanus protein conjugate vaccine [corrected] for prevention of pneumonia and meningitis in Gambian infants. Lancet 1997; 349(9060): 1191–7. |  | x |  |  | x |  |  |
| Mulholland K, Levine O, Nohynek H, Greenwood BW. Evaluation of vaccines for the prevention of pneumonia in children in developing countries. Epidemiol Rev 1999; 21: 43–55. |  | x |  |  |  |  |  |
| Nascimento-Carvalho C.M, Ribeiro C.T, Viriato D, Souza A.L, Cardoso M.R.A, Barral A, Araujo-Neto C.A, Oliveira J.R, Sobral L.S, Saukkoriipi A, Paldanius M, Leinonen M, Vainionpaa R, Ruuskanen O. The role of respiratory viral infections among children hospitalized for community-acquired pneumonia in a developing country. Pediatr. Infect. Dis. J. 2008; 27(10): 939–41. |  |  | x | x | x | x | x |
| Ngeow YF, Weil AF, Khairullah NS, Yusof MY, Luam L, Gaydos C, Quinn TC. Young Malaysian children with lower respiratory tract infections show low incidence of chlamydial infection. Journal of paediatrics and child health 1997; 33(5): 422–5. |  |  | x | x | x | x | x |
| Olsen S.J, Thamthitiwat S, Baggett H.C, Maloney S, Peruski L.F, Chantra S, Chittaganpitch M, Sawanpanyalert P, Fry A.M, Simmerman J.M, Peret T.C.T, Erdman D, Benson R, Thacker L, Tondella M.L, Winchell J, Fields B, Talkington D, Nicholson W.L, . Incidence of respiratory pathogens in persons hospitalized with pneumonia in two provinces in Thailand. Epidemiol. Infect. 2010; 138(12): 1811–22. |  |  | x | x |  | x |  |
| Rahman M, Huq F, Sack DA, Butler T, Azad AK, Alam A, Nahar N, Islam M. Acute lower respiratory tract infections in hospitalized patients with diarrhea in Dhaka, Bangladesh. Reviews of infectious diseases 1990; 12 Suppl 8: S899–906. |  |  | x | x | x | x | x |
| Reyes S, Montull B, Martinez R, Cordoba J, Molina J., Marti V, Martinez A, Ramirez P, Menendez R. Risk factors of A/H1N1 etiology in pneumonia and its impact on mortality. Respiratory Medicine 2011; 105(9): 1404–11. |  |  |  | x | x | x |  |
| Saito A, Kohno S, Matsushima T, Watanabe A, Oizumi K, Yamaguchi K, Oda H. Prospective multicenter study of the causative organisms of community-acquired pneumonia in adults in Japan. Journal of Infection and Chemotherapy 2006; 12(2): 63–9. |  |  |  | x | x |  |  |
| Shann F, Walters S, Pifer LL, Graham DM, Jack I, Uren E, Birch D, Stallman ND. Pneumonia associated with infection with pneumocystis, respiratory syncytial virus, chlamydia, mycoplasma, and cytomegalovirus in children in Papua New Guinea. British medical journal (Clinical research ed.) 1986; 292(6516): 314–7. |  |  | x |  |  |  |  |
| Shibli F, Flatau E, Nitzan O, Chazan B, Edelstein H, Raz R, Colodner R, Blondheim O. Etiology of community-acquired pneumonia in hospitalized patients in Northern Israel. Isr. Med. Assoc. J. 2010; 12(8): 477–82. |  |  | x | x |  | x |  |
| Sunakorn P, Chunchit L, Niltawat S, Wangweerawong M, Jacobs RF. Epidemiology of acute respiratory infections in young children from Thailand. The Pediatric infectious disease journal 1990; 9(12): 873–7. |  |  | x |  |  | x |  |
| Sung RYT, Cheng AFB, Chan RCK, Tam JS, Oppenheimer SJ. Epidemiology and Etiology of Pneumonia in Children in Hong Kong. Clinical Infectious Diseases 1993; 17(5): 894–6. |  |  | x | x | x | x | x |
| Tajima T, Nakayama E, Kondo Y, Hirai F, Ito H, Iitsuka T, Momomura M, Kutsuma H, Kodaka Y, Funaki N, Yanagawa Y, Ubukata K. Etiology and clinical study of community-acquired pneumonia in 157 hospitalized children. Journal of Infection and Chemotherapy 2006; 12(6): 372–9. |  |  | x | x | x | x | x |
| Takayanagi N, Hara K, Tokunaga D, Takaku Y, Minagawa S, Tsuchiya Y, Hijikata N, Yamaji T, Saito H, Ubukata M, Kurashima K, Yanagisawa T, Sugita Y. Etiology and outcome of community-acquired pneumonia in relation to age and severity in hospitalized adult patients. the journal of the Japanese Respiratory Society 2006; 44(12): 906–15. |  |  |  | x | x | x |  |
| Tupasi TE, Lucero MG, Magdangal DM, Mangubat NV, Sunico ME, Torres CU, de Leon LE, Paladin JF, Baes L, Javato MC. Etiology of acute lower respiratory tract infection in children from Alabang, Metro Manila. Reviews of infectious diseases 1990; 12 Suppl 8: S929–39. |  |  | x | x | x | x | x |
| Ygreda J.P, Perez F.L, Galarza R.R, Da Fieno J.T, Moreno V.S, Sanchez C.C, Gadea S.M.D.S, Guerra G.H. Perfil etiol—gico de la neumon’a adquirida en la comunidad en ni–os de 2 a 59 meses en dos zonas ecol—gicamente distintas del Perœ. Arch. Argent. Pediatr. 2010; 108(6): 516–23. |  |  | x | x | x | x | x |
| Zhang Q, MacDonald N.E, Guo Z. Vaccine preventable community-acquired pneumonia in hospitalized children in Northwest China. Pediatr. Infect. Dis. J. 2011; 30(1): 7–10. |  |  | x | x | x | x | x |
