# Supplementary material for: Deconstructing the differences: a comparison of GBD 2010 and CHERG’s approach to estimating the mortality burden of diarrhea, pneumonia, and their etiologies
Source: BMC Infect Dis. 2015 Jan 16;15:16. doi: 10.1186/s12879-014-0728-4 (PMC4305232; doi:10.1186/s12879-014-0728-4)
Supplement: Supplementary file 3 — Sources for the diarrhea etiology models used by GBD 2010 [6] and CHERG [10]. [file 12879_2014_728_MOESM3_ESM.docx]

**Additional file 3: Table S3**

**Sources for the diarrhea etiology models used by GBD 2010 [6] and CHERG [10]**

| Diarrhea Etiology Source List | GBD 2010 | | | | | | | | | | | | | | | | | | CHERG (LANATA ET AL.) | | | | | | | | | | | | | | | | | | | | | | | |
| --- | --- | --- | --- | --- | --- | --- | --- | --- | --- | --- | --- | --- | --- | --- | --- | --- | --- | --- | --- | --- | --- | --- | --- | --- | --- | --- | --- | --- | --- | --- | --- | --- | --- | --- | --- | --- | --- | --- | --- | --- | --- | --- |
|  | **Amoebiosis** | **Campylobacter** | **Cholera** | | **Crypto** | | **Diarrhea** | | **Enteropathogenic** | | **Enterotoxigenic** | | **Rotavirus** | | **Salmonella (other)** | | **Shigellosis** | | **Adenovirus** | **Astrovirus** | | | **Calicivirus** | **Campylobacter** | | **Cholera** | | **Crypto** | | **Entamoeba histolytica** | | **Enteropathogenic** | **Enterotoxigenic** | | | **Giardia lamblia** | **Rotavirus** | | **Salmonella (all)** | | **Shigellosis** | |
| Abenova UA, Kusmukhambetova RA. The characteristics of the electrophoretic types of rotaviruses isolated from sick children in Kazakhstan. Vopr. Virusol. 1992; 37(2): 120–2. |  |  |  | |  | | X | |  | |  | | X | |  | | X | |  |  | | |  |  | |  | |  | |  | |  |  | | |  |  | |  | |  | |
| Abugalia M, Cuevas L, Kirby A, Dove W, Nakagomi O, Nakagomi T, Kara M, Gweder R, Smeo M, Cunliffe N. Clinical features and molecular epidemiology of rotavirus and norovirus infections in Libyan children. J Med Virol. 2011 Oct;83(10):1849-56. |  |  |  | |  | |  | |  | |  | |  | |  | |  | |  |  | | | X |  | |  | |  | |  | |  |  | | |  | X | |  | |  | |
| Ahmed MU, Lam MM, Chowdhury NS, Haque MM, Shahid N, Kobayashi N, Taniguchi K, Urasawa T, Urasawa S. Analysis of human rotavirus g serotype in bangladesh by enzyme-linked immunosorben assay and polymerase chain reaction. J Diarrhoeal Dis Res. 1999 Mar;17(1):22-7. Immunosorbent assay and polymerase chain reaction. |  |  | |  | |  | |  | |  | |  | |  | |  | |  |  | |  |  | | |  | |  | |  | |  |  | |  |  | | | X | |  |  |  |
| Ajjampur SS, Liakath FB, Kannan A, Rajendran P, Sarkar R, Moses PD, Simon A, Agarwal I, Mathew A, O'Connor R, Ward H, Kang G. Multisite study of cryptosporidiosis in children with diarrhea in India. J Clin Microbiol. 2010 Jun;48(6):2075-81 |  |  |  | |  | |  | |  | |  | |  | |  | |  | |  |  | | |  |  | |  | | X | |  | |  |  | | |  |  | |  | |  | |
| Akinyemi KO, Oyefolu AO, Opere B, Otunba-Payne VA, Oworu AO. Escherichia coli in patients with acute gastroenteritis in Lagos, Nigeria. East Afr Med J 1998; 75(9): 512–5. |  |  |  | |  | |  | | X | | X | |  | | X | |  | |  |  | | |  |  | |  | |  | |  | |  |  | | |  |  | |  | |  | |
| Al Awaidy SA, Bawikar S, Al Busaidy S, Baqiani S, Al Abedani I, Varghese R, Abdoan HS, Al Abdoon H, Bhatnagar S, Al Hasini KS, Mohan P, Shah S, Elamir E, Klena J, Ahmed SF, Teleb N, Parashar U, Patel MM. Considerations for introduction of a rotavirus vaccine in Oman: rotavirus disease and economic burden. J. Infect. Dis. 2009; 200 Suppl 1: S248–53. |  |  |  | |  | | X | |  | |  | | X | |  | | X | |  |  | | |  |  | |  | |  | |  | |  |  | | |  |  | |  | |  | |
| Al-Gallas N, Bahri O, Bouratbeen A, Ben Haasen A, Ben Aissa R. Etiology of acute diarrhea in children and adults in Tunis, Tunisia, with emphasis on diarrheagenic Escherichia coli: prevalence, phenotyping, and molecular epidemiology. Am. J. Trop. Med. Hyg. 2007; 77(3): 571–82. |  | X | X | |  | | X | | X | | X | | X | | X | | X | |  |  | | |  |  | |  | |  | |  | | X |  | | |  |  | |  | |  | |
| Albert MJ, Rotimi VO, Dhar R, Silpikurian S, Pacsa AS, Molla AM, Szucs G. Diarrhoeagenic Escherichia coli are not a significant cause of diarrhoea in hospitalised children in Kuwait. BMC Microbiol. 2009 Mar 30;9:62. |  |  |  | |  | |  | |  | |  | |  | |  | |  | |  |  | | |  |  | |  | |  | |  | |  |  | | |  |  | |  | |  | |
| Aloun DS, Nyambat B, Phetsouvanh R, Douangboupha V, Keonakhone P, Xoumphonhphakdy B, Vongsouvath M, Kirkwood C, Bogdanovic-Sakran N, Kilgore PE. Rotavirus diarrhoea among children aged less than 5 years at Mahosot Hospital, Vientiane, Lao PDR. Vaccine 2009; 27 Suppl 5: F85–8. |  |  |  | |  | | X | |  | |  | | X | |  | | X | |  |  | | |  |  | |  | |  | |  | |  |  | | |  | X | |  | |  | |
| Amadi B, Kelly P, Mwiya M, Mulwazi E, Sianongo S, Changwe F, Thomson M, Hachungula J, Watuka A, Walker-Smith J, Chintu C. Intestinal and systemic infection, HIV, and mortality in Zambian children with persistent diarrhea and malnutrition. J. Pediatr. Gastroenterol. 2001; 32(5): 550–4. |  |  | X | | X | |  | |  | |  | |  | | X | |  | |  |  | | |  |  | |  | |  | |  | |  |  | | |  |  | |  | |  | |
| Ani A, Takahashi M, Saida H, Taniguchi H, Takahashi T, Sato M. Bacterial diarrhoeas in Jos, Nigeria. Jpn J Trop Med Hyg 1992;20(4):283-90. |  |  |  | |  | |  | |  | |  | |  | |  | |  | |  |  | | |  |  | |  | |  | |  | |  |  | | |  |  | |  | |  | |
| Antsupova AS, Al’tova EE, Zalesskikh AF, Epifanova NV, Dombrovskaia LK. Data on the epidemiology of rotavirus infection. Zh. Mikrobiol. Epidemiol. Immunobiol. 1988; 5: 34–7. |  |  |  | |  | | X | |  | |  | | X | |  | | X | |  |  | | |  |  | |  | |  | |  | |  |  | | |  |  | |  | |  | |
| Antsupova AS, Trofimova MN, Epifanova NV, Troitskaia MV. Electron microscopic diagnosis of viral diseases. Vopr. Virusol. 1984; 29(3): 316–9. |  |  |  | |  | | X | |  | |  | | X | |  | | X | |  |  | | |  |  | |  | |  | |  | |  |  | | |  |  | |  | |  | |
| Araújo IT, Fialho AM, de Assis RMS, Rocha M, Galvão M, Cruz CM, Ferreira MSR, Leite JPG. Rotavirus strain diversity in Rio de Janeiro, Brazil: characterization of VP4 and VP7 genotypes in hospitalized children. J. Trop. Pediatr. 2002; 48(4): 214–8. |  |  |  | |  | | X | |  | |  | | X | |  | | X | |  |  | | |  |  | |  | |  | |  | |  |  | | |  |  | |  | |  | |
| Arista S, Giovannelli L, Pistoia D, Cascio A, Parea M, Gerna G. Electropherotypes, subgroups and serotypes of human rotavirus strains causing gastroenteritis in infants and young children in Palermo, Italy, from 1985 to 1989. Res Virol. 1990 Jul-Aug;141(4):435-48. |  |  |  | |  | |  | |  | |  | |  | |  | |  | |  |  | | |  |  | |  | |  | |  | |  |  | | |  | X | |  | |  | |
| Baffone W, Ciaschini G, Pianetti A, Brandi G, Casaroli A, Bruscolini F. Detection of Escherichia coli O157:H7 and other intestinal pathogens in patients with diarrhoeal disease. Eur J Epidemiol. 2001;17(1):97-9. |  |  |  | |  | |  | |  | |  | |  | |  | |  | |  |  | | |  | X | |  | |  | |  | |  |  | | |  |  | | X | | X | |
| Bahl R, Ray P, Subodh S, Shambharkar P, Saxena M, Parashar U, Gentsch J, Glass R, Bhan MK; Delhi Rotavirus Study Group. Incidence of severe rotavirus diarrhea in New Delhi, India, and G and P types of the infecting rotavirus strains.J Infect Dis. 2005 Sep 1;192 Suppl 1:S114-9. |  |  |  | |  | |  | |  | |  | |  | |  | |  | |  |  | | |  |  | |  | |  | |  | |  |  | | |  | X | |  | |  | |
| Banerjee I, Gladstone BP, Le Fevre AM, Ramani S, Iturriza-Gomara M, Gray JJ, Brown DW, Estes MK, Muliyil JP, Jaffar S, Kang G. Neonatal infection with G10P[11] rotavirus did not confer protection against subsequent rotavirus infection in a community cohort in Vellore, South India. J. Infect. Dis. 2007; 195(5): 625–32. |  |  |  | |  | | X | |  | |  | | X | |  | | X | |  |  | | |  |  | |  | |  | |  | |  |  | | |  |  | |  | |  | |
| Baqui AH, Black RE, Sack RB, Chowdhury HR, Yunus M, Siddique AK. Malnutrition, cell-mediated immune deficiency, and diarrhea: a community-based longitudinal study in rural Bangladeshi children. Am. J. Epidemiol. 1993; 137(3): 355–65. |  |  | X | |  | |  | |  | |  | |  | |  | |  | |  |  | | |  |  | |  | |  | |  | |  |  | | |  |  | |  | |  | |
| Baqui AH, Sack RB, Black RE, Haider K, Hossain A, Alim AR, Yunus M, Chowdhury HR, Siddique AK. Enteropathogens associated with acute and persistent diarrhea in Bangladeshi children less than 5 years of age. J. Infect. Dis. 1992; 166(4): 792–6. |  | X |  | |  | | X | | X | |  | | X | |  | | X | |  |  | | |  | X | | X | | X | | X | | X | X | | | X | X | |  | | X | |
| Baqui AH, Yunus MD, Zaman K, Mitra AK, Hossain KM. Surveillance of patients attending a rural diarrhoea treatment centre in Bangladesh. Trop Geogr Med 1991; 43(1-2): 17–22. | X | X |  | |  | |  | |  | | X | |  | |  | |  | |  |  | | |  |  | |  | |  | |  | |  |  | | |  |  | |  | |  | |
| Barnes GL, Uren E, Stevens KB, Bishop RF. Etiology of acute gastroenteritis in hospitalized children in Melbourne, Australia, from April 1980 to March 1993. J Clin Microbiol. 1998 Jan;36(1):133-8. |  |  |  | |  | |  | |  | |  | |  | |  | |  | |  |  | | |  | X | |  | |  | |  | |  |  | | |  | X | | X | |  | |
| Barreira DM, Ferreira MS, Fumian TM, Checon R, de Sadovsky AD, Leite JP, Miagostovich MP, Spano LC.Viral load and genotypes of noroviruses in symptomatic and asymptomatic children in Southeastern Brazil. J Clin Virol. 2010 Jan;47(1):60-4. |  |  |  | |  | |  | |  | |  | |  | |  | |  | |  |  | | |  |  | |  | |  | |  | |  |  | | |  |  | |  | |  | |
| Bartlett AV, Torun B, Morales C, Cano F, Cruz JR. Oral gentamicin is not effective treatment for persistent diarrhea. Acta Paediatr Suppl 1992; 381: 149–54. | X | X |  | | X | | X | | X | | X | | X | | X | | X | |  |  | | |  |  | |  | |  | |  | |  |  | | |  |  | |  | |  | |
| Battikhi MNG. Epidemiological study on Jordanian patients suffering from diarrhoea. New Microbiol 2002; 25(4): 405–12. |  | X |  | |  | | X | | X | |  | | X | | X | | X | |  |  | | |  |  | |  | |  | |  | |  |  | | |  |  | |  | |  | |
| Benhafid M, Youbi M, Klena JD, Gentsch JR, Teleb N, Widdowson M-A, Elaouad R. Epidemiology of rotavirus gastroenteritis among children <5 years of age in Morocco during 1 year of sentinel hospital surveillance, June 2006-May 2007. J. Infect. Dis. 2009; 200 Suppl 1: S70–75. |  |  |  | |  | | X | |  | |  | | X | |  | | X | |  |  | | |  |  | |  | |  | |  | |  |  | | |  |  | |  | |  | |
| Bern C, Hernandez B, Lopez MB, Arrowood MJ, De Merida AM, Klein RE. The contrasting epidemiology of Cyclospora and Cryptosporidium among outpatients in Guatemala. Am. J. Trop. Med. Hyg. 2000; 63(5-6): 231–5. |  |  |  | | X | |  | |  | |  | |  | |  | |  | |  |  | | |  |  | |  | |  | |  | |  |  | | |  |  | |  | |  | |
| Bhadra RK, Dutta P, Bhattacharya SK, Dutta SK, Pal SC, Nair GB. Campylobacter species as a cause of diarrhoea in children in Calcutta. J Infect. 1992 Jan;24(1):55-62. |  |  |  | |  | |  | |  | |  | |  | |  | |  | |  |  | | |  | X | | X | |  | |  | |  | X | | |  | X | | X | | X | |
| Bhan MK, Bhandari N, Sazawal S, Clemens J, Raj P, Levine MM, Kaper JB. Descriptive epidemiology of persistent diarrhoea among young children in rural northern India. Bull 1989; 67(3): 281–8. | X | X |  | |  | | X | | X | | X | | X | | X | | X | |  |  | | |  |  | |  | |  | |  | |  |  | | |  |  | |  | |  | |
| Bhan MK, Khoshoo V, Sommerfelt H, Raj P, Sazawal S, Srivastava R. Enteroaggregative Escherichia coli and Salmonella associated with nondysenteric persistent diarrhea. Pediatr. Infect. Dis. J. 1989; 8(8): 499–502. |  | X |  | |  | | X | |  | |  | | X | |  | | X | |  |  | | |  |  | |  | |  | |  | |  |  | | |  |  | |  | |  | |
| Bhandari N, Bahl R, Dua T, Kumar R, Srivastava R. Role of protozoa as risk factors for persistent diarrhea. Indian J Pediatr 1999; 66(1): 21–6. | X |  |  | | X | |  | |  | |  | |  | |  | |  | |  |  | | |  |  | |  | |  | |  | |  |  | | |  |  | |  | |  | |
| Bittencourt JA, Arbo E, Malysz AS, Oravec R, Dias C. Seasonal and age distribution of rotavirus infection in Porto Alegre--Brazil. Braz J Infect Dis 2000; 4(6): 279–83. |  |  |  | |  | | X | |  | |  | | X | |  | | X | |  |  | | |  |  | |  | |  | |  | |  |  | | |  |  | |  | |  | |
| Black RE, Merson MH, Rahman AS, Yunus M, Alim AR, Huq I, Yolken RH, Curlin GT. A two-year study of bacterial, viral, and parasitic agents associated with diarrhea in rural Bangladesh. J. Infect. Dis. 1980; 142(5): 660–4. |  |  | X | |  | | X | |  | | X | | X | |  | | X | |  |  | | |  |  | |  | |  | |  | |  |  | | |  |  | |  | |  | |
| Blake PA, Ramos S, MacDonald KL, Rassi V, Gomes TA, Ivey C, Bean NH, Trabulsi LR. Pathogen-specific risk factors and protective factors for acute diarrheal disease in urban Brazilian infants. J Infect Dis. 1993 Mar;167(3):627-32. |  |  |  | |  | |  | |  | |  | |  | |  | |  | |  |  | | |  |  | |  | |  | |  | | X | X | | |  | X | | X | | X | |
| Bok K, Castagnaro N, Borsa A, Nates S, Espul C, Fay O, Fabri A, Grinstein S, Miceli I, Matson DO, Gómez JA. Surveillance for rotavirus in Argentina. J Med Virol. 2001 Sep;65(1):190-8. |  |  |  | |  | |  | |  | |  | |  | |  | |  | |  |  | | |  |  | |  | |  | |  | |  |  | | |  | X | |  | |  | |
| Bonkoungou IJ, Sanou I, Bon F, Benon B, Coulibaly SO, Haukka K, Traoré AS, Barro N. Epidemiology of rotavirus infection among young children with acute diarrhoea in Burkina Faso.BMC Pediatr. 2010 Dec 20;10:94. |  |  |  | |  | |  | |  | |  | |  | |  | |  | |  |  | | |  |  | |  | |  | |  | |  |  | | |  | X | |  | |  | |
| Brandonisio O, Marangi A, Panaro MA, Marzio R, Natalicchio MI, Zizzadoro P, De Santis U. Prevalence of Cryptosporidium in children with enteritis in southern Italy. Eur. J. Epidemiol. 1996; 12(2): 187–90. |  |  |  | | X | |  | |  | |  | |  | |  | |  | |  |  | | |  |  | |  | |  | |  | |  |  | | |  |  | |  | |  | |
| Cabrita J, Pires I, Vlaes L, Coignau H, Levy J, Goossens H, Goncalves AP, De Mol P, Butzler JP. Campylobacter enteritis in Portugal: epidemiological features and biological markers. Eur. J. Epidemiol. 1992; 8(1): 22–6. |  | X |  | |  | |  | |  | |  | |  | | X | |  | |  |  | | |  |  | |  | |  | |  | |  |  | | |  |  | |  | |  | |
| Cama RI, Parashar UD, Taylor DN, Hickey T, Figueroa D, Ortega YR, Romero S, Perez J, Sterling CR, Gentsch JR, Gilman RH, Glass RI. Enteropathogens and other factors associated with severe disease in children with acute watery diarrhea in Lima, Peru. J. Infect. Dis. 1999; 179(5): 1139–44. |  | X | X | | X | | X | |  | | X | | X | |  | | X | |  | X | | |  | X | |  | | X | |  | |  | X | | | X | X | |  | | X | |
| Candia N, Parra GI, Chirico M, Velázquez G, Farina N, Laspina F, Shin J, De Sierra MJ, Russomando G, Arbiza. J.Acta Virol. 2003;47(3):137-40. |  |  |  | |  | |  | |  | |  | |  | |  | |  | |  |  | | |  |  | |  | |  | |  | |  |  | | |  | X | |  | |  | |
| Cardoso D das D de P, Soares CMA, Dias e Souza MB de L, De Azevedo M da SP, Martins RMB, Queiróz DA de O, De Brito WMED, Munford V, Rácz ML. Epidemiological features of rotavirus infection in Goiânia, Goiás, Brazil, from 1986 to 2000. Mem. Inst. Oswaldo Cruz. 2003; 98(1): 25–9. |  |  |  | |  | | X | |  | |  | | X | |  | | X | |  |  | | |  |  | |  | |  | |  | |  |  | | |  | X | |  | |  | |
| Carlos CC, Inobaya MT, Bresee JS, Lagrada ML, Olorosa AM, Kirkwood CD, Widdowson M-A. The burden of hospitalizations and clinic visits for rotavirus disease in children aged <5 years in the Philippines. J. Infect. Dis. 2009; 200 Suppl 1: S174–18. |  |  |  | |  | | X | |  | |  | | X | |  | | X | |  |  | | |  |  | |  | |  | |  | |  |  | | |  | X | |  | |  | |
| Carmona RCC, Timenetsky M do CST, Da Silva FF, Granato CFH. Characterization of rotavirus strains from hospitalized and outpatient children with acute diarrhoea in São Paulo, Brazil. Brazil. J. Med. Virol. 2004; 74(1): 166–72. |  |  |  | |  | | X | |  | |  | | X | |  | | X | |  |  | | |  |  | |  | |  | |  | |  |  | | |  |  | |  | |  | |
| Ceyhan M, Alhan E, Salman N, Kurugol Z, Yildirim I, Celik U, Keser M, Koturoglu G, Tezer H, Bulbul EK, Karabocuoglu M, Halicioglu O, Anis S, Pawinski R. Multicenter prospective study on the burden of rotavirus gastroenteritis in Turkey, 2005-2006: a hospital-based study. J. Infect. Dis. 2009; 200 Suppl 1: S234–8. |  |  |  | |  | | X | |  | |  | | X | |  | | X | |  |  | | |  |  | |  | |  | |  | |  |  | | |  | X | |  | |  | |
| Chacin-Bonilla L, Bonilla MC, Soto-Torres L, Rios-Candida Y, Sardina M, Enmanuels C, Parra AM, Sanchez-Chavez Y. Cryptosporidium parvum in children with diarrhea in Zulia State, Venezuela. Am J Trop Med Hyg. 1997 Apr;56(4):365-9. |  |  |  | |  | |  | |  | |  | |  | |  | |  | |  |  | | |  |  | |  | | X | |  | |  |  | | |  |  | |  | |  | |
| Chai PF, Lee WS. Out-of-pocket costs associated with rotavirus gastroenteritis requiring hospitalization in Malaysia. Vaccine 2009; 27 Suppl 5: F112–115. |  |  |  | |  | | X | |  | |  | | X | |  | | X | |  |  | | |  |  | |  | |  | |  | |  |  | | |  |  | |  | |  | |
| Chakravarti A, Broor S, Natarajan R, Setty VS, Mittal SK. Epidemiological and clinical characteristics of acute diarrhoea in children due to human rotavirus. J Trop Pediatr. 1992 Aug;38(4):192-3. |  |  |  | |  | |  | |  | |  | |  | |  | |  | |  |  | | |  |  | |  | |  | |  | |  |  | | |  | X | |  | |  | |
| Chakravarti A, Chauhan MS, Sharma A, Verma V.Distribution of human rotavirus G and P genotypes in a hospital setting from Northern India.Southeast Asian J Trop Med Public Health. 2010 Sep;41(5):1145-52. |  |  |  | |  | |  | |  | |  | |  | |  | |  | |  |  | | |  |  | |  | |  | |  | |  |  | | |  |  | |  | |  | |
| Chen KT, Chen PY, Tang RB, Huang YF, Lee PI, Yang JY, Chen HY, Bresee J, Hummelman E, Glass R. Sentinel hospital surveillance for rotavirus diarrhea in Taiwan, 2001-2003.J Infect Dis. 2005 Sep 1;192 Suppl 1:S44-8. |  |  |  | |  | |  | |  | |  | |  | |  | |  | |  |  | | |  |  | |  | |  | |  | |  |  | | |  | X | |  | |  | |
| Cheng WX, Ye XH, Yang XM, Li YN, Jin M, Jin Y, Duan ZJ.Epidemiological study of human calicivirus infection in children with gastroenteritis in Lanzhou from 2001 to 2007.Arch Virol. 2010 Apr;155(4):553-5. |  |  |  | |  | |  | |  | |  | |  | |  | |  | |  |  | | | X |  | |  | |  | |  | |  |  | | |  |  | |  | |  | |
| Cheun H-I, Cho S-H, Lee J-H, Lim Y-Y, Jeon J-H, Yu J-R, Kim T-S, Lee W-J, Cho S-H, Lee D-Y, Park M-S, Jeong H-S, Chen D-S, Ji Y-M, Kwon M-H. Infection status of hospitalized diarrheal patients with gastrointestinal protozoa, bacteria, and viruses in the Republic of Korea. Korean J. Parasitol. 2010; 48(2): 113–20. |  | X |  | | X | | X | |  | |  | | X | | X | | X | | X | X | | | X | X | |  | | X | | X | |  | X | | | X | X | | X | | X | |
| Chhabra P, Dhongade RK, Kalrao VR, Bavdekar AR, Chitambar SD.Epidemiological, clinical, and molecular features of norovirus infections in western India. J Med Virol. 2009 May;81(5):922-32. |  |  |  | |  | |  | |  | |  | |  | |  | |  | |  |  | | |  |  | |  | |  | |  | |  |  | | |  |  | |  | |  | |
| Chintu C, Luo C, Baboo S, Khumalo-Ngwenya B, Mathewson J, DuPont HL, Zumla A. Intestinal parasites in HIV-seropositive Zambian children with diarrhoea. J. Trop. Pediatr. 1995; 41(3): 149–52. |  |  |  | | X | |  | |  | |  | |  | |  | |  | |  |  | | |  |  | |  | |  | |  | |  |  | | |  |  | |  | |  | |
| Chowdhury F, Rahman MA, Begum YA, Khan AI, Faruque AS, Saha NC, Baby NI, Malek MA, Kumar AR, Svennerholm AM, Pietroni M, Cravioto A, Qadri F.Impact of rapid urbanization on the rates of infection by Vibrio cholerae O1 and enterotoxigenic Escherichia coli in Dhaka, Bangladesh. J Pediatr (Rio J). 2011 Sep-Oct;87(5):445-9. |  |  |  | |  | |  | |  | |  | |  | |  | |  | |  |  | | |  |  | |  | |  | |  | |  |  | | |  |  | |  | |  | |
| Cilla G, Gomariz M, Montes M, Mendiburu MI, Pérez-Yarza EG, Pérez-Trallero E. Incidence of hospitalization due to community-acquired rotavirus infection: a 12-year study (1996-2008). Epidemiol. Infect. 2010; 138(9): 1235–41. |  |  |  | |  | | X | |  | |  | | X | |  | | X | |  |  | | |  |  | |  | |  | |  | |  |  | | |  |  | |  | |  | |
| Cilli A, Luchs A, Morillo SG, Costa FF, Carmona Rde C, Timenetsky Mdo C. Characterization of rotavirus and norovirus strains: a 6-year study (2004-2009). J Pediatr (Rio J). 2011 Sep-Oct;87(5):445-9. |  |  |  | |  | |  | |  | |  | |  | |  | |  | |  |  | | | X |  | |  | |  | |  | |  |  | | |  | X | |  | |  | |
| Cohen MB, Nataro JP, Bernstein DI, Hawkins J, Roberts N, Staat MA. Prevalence of diarrheagenic Escherichia coli in acute childhood enteritis: a prospective controlled study. J Pediatr. 2005 Jan;146(1):54-61. |  |  |  | |  | |  | |  | |  | |  | |  | |  | |  |  | | |  |  | |  | |  | |  | | X | X | | |  |  | |  | |  | |
| Cortes J, Arvelo W, Lopez B, Reyes L, Kerin T, Gautam R, Patel M, Parashar U, Lindblade KA.Rotavirus disease burden among children <5 years of age--Santa Rosa, Guatemala, 2007-2009.Trop Med Int Health. 2012 Feb;17(2):254-9. |  |  |  | |  | |  | |  | |  | |  | |  | |  | |  |  | | |  |  | |  | |  | |  | |  |  | | |  | X | |  | |  | |
| Cruz JR, Caceres P, Cano F, Flores J, Bartlett A, Torun B. Adenovirus types 40 and 41 and rotaviruses associated with diarrhea in children from Guatemala. J Clin Microbiol. 1990 Aug;28(8):1780-4. |  |  |  | |  | |  | |  | |  | |  | |  | |  | |  |  | | |  | X | |  | | X | | X | | X | X | | | X | X | | X | | X | |
| Cunliffe NA, Ngwira BM, Dove W, Thindwa BDM, Turner AM, Broadhead RL, Molyneux ME, Hart CA. Epidemiology of rotavirus infection in children in Blantyre, Malawi, 1997-2007. J. Infect. Dis. 2010; 202 Suppl: S168–174. |  |  |  | |  | | X | |  | |  | | X | |  | | X | |  |  | | |  |  | |  | |  | |  | |  |  | | |  | X | |  | |  | |
| Da Silva Domingues AL, Da Silva Vaz MG, Moreno M, Câmara FP. Molecular epidemiology of group A rotavirus causing acute diarrhea in infants and young children hospitalized in Rio de Janeiro, Brazil, 1995-1996. Braz J Infect Dis 2000; 4(3): 119–25. |  |  |  | |  | | X | |  | |  | | X | |  | | X | |  |  | | |  |  | |  | |  | |  | |  |  | | |  | X | |  | |  | |
| Dagan R, Bar-David Y, Sarov B, Katz M, Kassis I, Greenberg D, Glass RI, Margolis CZ, Sarov I. Rotavirus diarrhea in Jewish and Bedouin children in the Negev region of Israel: epidemiology, clinical aspects and possible role of malnutrition in severity of illness.Pediatr Infect Dis J. 1990 May;9(5):314-21. |  |  |  | |  | |  | |  | |  | |  | |  | |  | |  |  | | |  |  | |  | |  | |  | |  |  | | |  | X | |  | |  | |
| Das P, Sengupta K, Dutta P, Bhattacharya MK, Pal SC, Bhattacharya SK. Significance of Cryptosporidium as an aetiologic agent of acute diarrhoea in Calcutta: a hospital based study. J Trop Med Hyg 1993; 96(2): 124–7. |  |  |  | | X | |  | |  | |  | |  | |  | |  | |  |  | | |  |  | |  | | X | |  | |  |  | | |  |  | |  | |  | |
| Das S, Sen A, Uma G, Varghese V, Chaudhuri S, Bhattacharya SK, Krishnan T, Dutta P, Dutta D, Bhattacharya MK, Mitra U, Kobayashi N, Naik TN. Genomic diversity of group A rotavirus strains infecting humans in eastern India.J Clin Microbiol. 2002 Jan;40(1):146-9. |  |  |  | |  | |  | |  | |  | |  | |  | |  | |  |  | | |  |  | |  | |  | |  | |  |  | | |  | X | |  | |  | |
| Dayan N, Revivo D, Even L, Elkayam O, Glikman D. Campylobacter is the leading cause of bacterial gastroenteritis and dysentery in hospitalized children in the Western Galilee Region in Israel. Epidemiol Infect. 2010 Oct;138(10):1405-6; |  |  |  | |  | |  | |  | |  | |  | |  | |  | |  |  | | |  | X | |  | |  | |  | |  |  | | |  |  | | X | | X | |
| de Andrade JA, de Oliveira JO, Fagundes Neto U. [Lethality in hospitalized infants with acute diarrhea: risk factors associated with death]. Rev Assoc Med Bras. 1999 Apr-Jun;45(2):121-7. |  |  |  | |  | |  | |  | |  | |  | |  | |  | |  |  | | |  | X | |  | | X | |  | | X | X | | | X | X | | X | | X | |
| De Grazia S, Platia MA, Rotolo V, Colomba C, Martella V, Giammanco GM. Surveillance of human astrovirus circulation in Italy 2002-2005: emergence of lineage 2c strains.Clin Microbiol Infect. 2011 Jan;17(1):97-101. |  |  |  | |  | |  | |  | |  | |  | |  | |  | |  | X | | |  |  | |  | |  | |  | |  |  | | |  |  | |  | |  | |
| De Oliveira LH, Danovaro-Holliday MC, Andrus JK, De Fillipis AMB, Gentsch J, Matus CR, Widdowson M-A. Sentinel hospital surveillance for rotavirus in latin american and Caribbean countries. J. Infect. Dis. 2009; 200 suppl 1: s131–9. |  |  |  | |  | | X | |  | |  | | X | |  | | X | |  |  | | |  |  | |  | |  | |  | |  |  | | |  |  | |  | |  | |
| Desai HS, Banker DD. Rotavirus infection among children in Bombay. Indian J Med Sci. 1993 Feb;47(2):27-33. |  |  |  | |  | |  | |  | |  | |  | |  | |  | |  |  | | |  |  | |  | |  | |  | |  |  | | |  | X | |  | |  | |
| Dey SK, Shimizu H, Phan TG, Hayakawa Y, Islam A, Salim AF, Khan AR, Mizuguchi M, Okitsu S, Ushijima H. Molecular epidemiology of adenovirus infection among infants and children with acute gastroenteritis in Dhaka City, Bangladesh. Infect Genet Evol. 2009 Jul;9(4):518-22. |  |  |  | |  | |  | |  | |  | |  | |  | |  | | X |  | | |  |  | |  | |  | |  | |  |  | | |  |  | |  | |  | |
| Diamanti E, Superti F, Tinari A, Marziano ML, Giovannangeli S, Tafaj F, Xhelili L, Gani D, Donelli G. An epidemiological study on viral infantile diarrhoea in Tirana. New Microbiol. 1996 Jan;19(1):9-14. |  |  |  | |  | |  | |  | |  | |  | |  | |  | |  |  | | |  |  | |  | |  | |  | |  |  | | |  | X | |  | |  | |
| Do TT, Bui TTH, Mølbak K, Phung DC, Dalsgaard A. Epidemiology and aetiology of diarrhoeal diseases in adults engaged in wastewater-fed agriculture and aquaculture in Hanoi, Vietnam. Trop. Med. Int. Health. 2007; 12 suppl 2: 23–33. |  | X |  | |  | | X | |  | | X | | X | | X | | X | |  |  | | |  |  | |  | |  | |  | |  |  | | |  |  | |  | |  | |
| Doan LT, Okitsu S, Nishio O, Pham DT, Nguyen DH, Ushijima H. Epidemiological features of rotavirus infection among hospitalized children with gastroenteristis in Ho Chi Minh City, Vietnam. J Med Virol. 2003 Apr;69(4):588-94. |  |  |  | |  | |  | |  | |  | |  | |  | |  | |  |  | | |  |  | |  | |  | |  | |  |  | | |  | X | |  | |  | |
| Donelli G, Superti F, Tinari A, Marziano ML, Caione D, Concato C, Menichella D. Viral childhood diarrhoea in Rome: a diagnostic and epidemiological study. New Microbiol. 1993 Jul;16(3):215-25. |  |  |  | |  | |  | |  | |  | |  | |  | |  | | X |  | | |  |  | |  | |  | |  | |  |  | | |  |  | |  | |  | |
| Duan ZJ, Liu N, Yang SH, Zhang J, Sun LW, Tang JY, Jin Y, Du ZQ, Xu J, Wu QB, Tong ZL, Gong ST, Qian Y, Ma JM, Liao XC, Widdowson MA, Jiang B, Fang ZY.Hospital-Based Surveillance of Rotavirus Diarrhea in the People's Republic of China, August 2003-July 2007.J Infect Dis. 2009 Nov 1;200 Suppl 1:S167-73. |  |  |  | |  | |  | |  | |  | |  | |  | |  | |  |  | | |  |  | |  | |  | |  | |  |  | | |  | X | |  | |  | |
| Dutta P, Mitra U, Rasaily R, Bhattacharya SK, Bhattacharya MK, Manna B, Gupta A, Kundu B. Assessing the cause of in-patients pediatric diarrheal deaths: an analysis of hospital records.Indian Pediatr. 1995 Mar;32(3):313-21. |  |  |  | |  | |  | |  | |  | |  | |  | |  | |  |  | | |  | X | | X | |  | |  | |  | X | | |  | X | | X | | X | |
| Dutta SR, Khalfan SA, Baiq BH, Philipose L, Fulayfil R. Epidemiology of rotavirus diarrhoea in children under five years in BahrainInt J Epidemiol. 1990 Sep;19(3):722-7. |  |  |  | |  | |  | |  | |  | |  | |  | |  | |  |  | | |  | X | |  | |  | |  | |  |  | | |  | X | | X | | X | |
| Echeverria P, Seriwatana J, Taylor DN, Yanggratoke S, Tirapat C. A comparative study of enterotoxigenic Escherichia coli, Shigella, Aeromonas, and Vibrio as etiologies of diarrhea in northeastern Thailand. Am. J. Trop. Med. Hyg. 1985; 34(3): 547–54. |  |  | X | |  | |  | |  | | X | |  | |  | |  | |  |  | | |  |  | |  | |  | |  | |  |  | | |  |  | |  | |  | |
| Eesteghamati A, Gouya M, Keshtkar A, Najafi L, Zali MR, Sanaei M, Yaghini F, El Mohamady H, Patel M, Klena JD, Teleb N. Sentinel hospital-based surveillance of rotavirus diarrhea in iran. J. Infect. Dis. 2009; 200 Suppl 1: S244–247. |  |  |  | |  | | X | |  | |  | | X | |  | | X | |  |  | | |  |  | |  | |  | |  | |  |  | | |  | X | |  | |  | |
| Espinoza F, Paniagua M, Hallander H, Svensson L, Strannegård O. Rotavirus infections in young Nicaraguan children. Pediatr. Infect. Dis. J. 1997; 16(6): 564–7. |  |  |  | |  | | X | |  | |  | | X | |  | | X | |  |  | | |  |  | |  | |  | |  | |  |  | | |  |  | |  | |  | |
| Espul C, Cuello H, Navarta LM, Mamani N, O’Ryan M, O’Ryan M. Characterization of antigenic types of circulating rotaviruses in Mendoza, Argentina based on typing of the external VP7 capsid protein. Acta Gastroenterol. Latinoam. 1993; 23(4): 211–6. |  |  |  | |  | | X | |  | |  | | X | |  | | X | |  |  | | |  |  | |  | |  | |  | |  |  | | |  |  | |  | |  | |
| Fagundes-Neto U, de Andrade JA. Acute diarrhea and malnutrition: lethality risk in hospitalized infants. J Am Coll Nutr. 1999 Aug;18(4):303-8. |  |  |  | |  | |  | |  | |  | |  | |  | |  | |  |  | | |  | X | |  | | X | |  | |  |  | | |  | X | | X | | X | |
| Fang GD, Lima AA, Martins CV, Nataro JP, Guerrant RL. Etiology and epidemiology of persistent diarrhea in northeastern Brazil: a hospital-based, prospective, case-control study. J. Pediatr. Gastroenterol. Nutr. 1995; 21(2): 137–44. |  |  |  | | X | | X | |  | |  | | X | |  | | X | |  |  | | |  |  | |  | | X | |  | | X | X | | |  | X | | X | | X | |
| Fernandes JV, Fonseca SM, Azevedo JC, Maranhão H de S, Fonseca MH, Dantas MT, Meissner R de V. Rotavirus detection in feces of children with acute diarrhea. J Pediatr (Rio J) 2000; 76(4): 300–4. |  |  |  | |  | | X | |  | |  | | X | |  | | X | |  |  | | |  |  | |  | |  | |  | |  |  | | |  |  | |  | |  | |
| Ferreccio C, Prado V, Ojeda A, Cayyazo M, Abrego P, Guers L, Levine MM. Epidemiologic patterns of acute diarrhea and endemic Shigella infections in children in a poor periurban setting in Santiago, Chile. Am J Epidemiol. 1991 Sep 15;134(6):614-27. |  |  |  | |  | |  | |  | |  | |  | |  | |  | |  |  | | |  |  | |  | |  | |  | |  |  | | |  |  | |  | | X | |
| Ferreira MS, Victoria M, Carvalho-Costa FA, Vieira CB, Xavier MP, Fioretti JM, Andrade J, Volotão EM, Rocha M, Leite JP, Miagostovich MP. Surveillance of norovirus infections in the state of Rio De Janeiro, Brazil 2005-2008.J Med Virol. 2010 Aug;82(8):1442-8. |  |  |  | |  | |  | |  | |  | |  | |  | |  | |  |  | | | X |  | |  | |  | |  | |  |  | | |  |  | |  | |  | |
| Figueroa-Quintanilla D, Salazar-Lindo E, Sack RB, León-Barúa R, Sarabia-Arce S, Campos-Sánchez M, Eyzaguirre-Maccan E. A controlled trial of bismuth subsalicylate in infants with acute watery diarrheal disease. N. Engl. J. Med. 1993; 328(23): 1653–8. |  | X |  | |  | | X | |  | |  | | X | | X | | X | |  |  | | |  |  | |  | |  | |  | |  |  | | |  |  | |  | |  | |
| Fischer TK, Rungoe C, Jensen CS, Breindahl M, Jørgensen TR, Nielsen JP, Jensen L, Malon M, Brændholt V, Fisker N, Hjelt K. The burden of rotavirus disease in Denmark 2009-2010. Pediatr Infect Dis J. 2011 Jul;30(7):e126-9. |  |  |  | |  | |  | |  | |  | |  | |  | |  | | X |  | | |  |  | |  | |  | |  | |  |  | | |  | X | |  | |  | |
| Fischer TK. Incidence of hospitalizations due to rotavirus gastroenteritis in Denmark. Acta Paediatr. 2001 Sep;90(9):1073-5. |  |  |  | |  | |  | |  | |  | |  | |  | |  | |  |  | | |  |  | |  | |  | |  | |  |  | | |  | X | |  | |  | |
| Flem E, Vainio K, Døllner H, Midgaard C, Bosse FJ, Rognlien AG, Rojahn A, Nordbo SA, Størvold G, Njølstad G, Wathne KO, Konsmo K, Aavitsland P. Rotavirus gastroenteritis in Norway: analysis of prospective surveillance and hospital registry data.Scand J Infect Dis. 2009;41(10):753-9. |  |  |  | |  | |  | |  | |  | |  | |  | |  | |  |  | | |  |  | |  | |  | |  | |  |  | | |  | X | |  | |  | |
| Flem ET, Kasymbekova KT, Vainio K, Gentsch J, Abdikarimov ST, Glass RI, Bresee JS. Rotavirus infection in hospitalized children and estimates of disease burden in Kyrgyzstan, 2005-2007. Vaccine 2009; 27 Suppl 5: F35–9. |  |  |  | |  | | X | |  | |  | | X | |  | | X | |  |  | | |  |  | |  | |  | |  | |  |  | | |  | X | |  | |  | |
| Flem ET, Musabaev E, Juraev R, Kerin T, Gentsch J, Glass RI, Bresee JS. Rotavirus gastroenteritis in uzbekistan: implications for vaccine policy in central Asia. J. Infect. Dis. 2009; 200 Suppl 1: S154–159. |  |  |  | |  | | X | |  | |  | |  | |  | |  | |  |  | | |  |  | |  | |  | |  | |  |  | | |  |  | |  | |  | |
| Fodha I, Chouikha A, Peenze I, De Beer M, Dewar J, Geyer A, Messaadi F, Trabelsi A, Boujaafar N, Taylor MB, Steele D. Identification of viral agents causing diarrhea among children in the Eastern Center of Tunisia. J Med Virol. 2006 Sep;78(9):1198-203. |  |  |  | |  | |  | |  | |  | |  | |  | |  | |  |  | | |  |  | |  | |  | |  | |  |  | | |  |  | |  | |  | |
| Forster J, Guarino A, Parez N, Moraga F, Román E, Mory O, Tozzi AE, De Aguileta AL, Wahn U, Graham C, Berner R, Ninan T, Barberousse C, Meyer N, Soriano-Gabarró M. Hospital-based surveillance to estimate the burden of rotavirus gastroenteritis among European children younger than 5 years of age. Pediatrics 2009; 123(3): e393–400. |  |  |  | |  | | X | |  | |  | | X | |  | | X | |  |  | | |  |  | |  | |  | |  | |  |  | | |  | X | |  | |  | |
| Gabutti G, Marsella M, Lazzara C, Fiumana E, Cavallaro A, Borgna-Pignatti C. Epidemiology and burden of rotavirus-associated hospitalizations in Ferrara, Italy. J Prev Med Hyg 2007; 48(1): 5–9. |  |  |  | |  | | X | |  | |  | | X | |  | | X | |  |  | | |  |  | |  | |  | |  | |  |  | | |  |  | |  | |  | |
| Gassama A, Sow PS, Fall F, Camara P, Guèye-N’diaye A, Seng R, Samb B, M’Boup S, Aïdara-Kane A. Ordinary and opportunistic enteropathogens associated with diarrhea in Senegalese adults in relation to human immunodeficiency virus serostatus. Int. J. Infect. Dis. 2001; 5(4): 192–8. | X | X |  | | X | | X | |  | |  | | X | | X | | X | |  |  | | |  |  | |  | |  | |  | |  |  | | |  |  | |  | |  | |
| Ghosh AR, Koley H, De D, Paul M, Nair GB, Sen D. Enterotoxigenic Escherichia coli associated diarrhoea among infants aged less than six months in Calcutta, India. Eur J Epidemiol. 1996 Feb;12(1):81-4. |  |  |  | |  | |  | |  | |  | |  | |  | |  | |  |  | | |  |  | |  | |  | |  | |  | X | | |  |  | |  | |  | |
| Ghosh AR, Nair GB, Dutta P, Pal SC, Sen D. Acute diarrhoeal diseases in infants aged below six months in hospital in Calcutta, India: an aetiological study. Trans R Soc Trop Med Hyg. 1991 Nov-Dec;85(6):796-8. |  |  |  | |  | |  | |  | |  | |  | |  | |  | |  |  | | |  | X | |  | | X | | X | | X |  | | |  | X | | X | | X | |
| Ghosh AR, Sehgal SC. Shigella infections among children in Andaman--an archipelago of tropical islands in Bay of Bengal. Epidemiol Infect. 1998 Aug;121(1):43-8. |  |  |  | |  | |  | |  | |  | |  | |  | |  | |  |  | | |  |  | |  | |  | |  | |  |  | | |  |  | |  | | X | |
| Ginevskaya VA, Amitina NN, Eremeeva TP, Shirman GA, Priimägi LS, Drozdov SG. Electropherotypes and serotypes of human rotavirus in Estonia in 1989-1992. Arch. Virol. 1994; 137(1-2): 199–207. |  |  |  | |  | | X | |  | |  | | X | |  | | X | |  |  | | |  |  | |  | |  | |  | |  |  | | |  |  | |  | |  | |
| Ginevskaya VA, Eremeeva TP, Zangaladze ED, Shirman GA, Kazantseva VA, Sakvarelidze LA, Drozdov SG. Analysis of rotaviral gastroenteritis in Tbilisi. Acta Virol 1991; 35(3): 232–7. |  |  |  | |  | | X | |  | |  | | X | |  | | X | |  |  | | |  |  | |  | |  | |  | |  |  | | |  |  | |  | |  | |
| Giordano MO, Depetris AR, Nates SV. Epidemiological survey of human rotavirus electropherotypes in young children in Cordoba city, Argentina, from 1979 to 1989. Revista argentina de microbiología 1995; 27: 1–10. |  |  |  | |  | | X | |  | |  | | X | |  | | X | | X | X | | |  |  | |  | |  | |  | |  |  | | |  | X | |  | |  | |
| Gómez JA, Bercovich J, Biscotti E, Siniawski S, Sternberg L, Montesi A. Diarrea por rotavirus: estudio prospectivo de 49 familias del partido de Avellaneda, provincia de Buenos Aires. Archivos argentinos de pediatría 1987; 85: 139–149. |  |  |  | |  | | X | |  | |  | | X | |  | | X | |  |  | | |  |  | |  | |  | |  | |  |  | | |  |  | |  | |  | |
| Gómez JA, Biscotti EL, Bercovich JA, Grinstein S. Epidemiology of human rotaviruses in Argentina as determined by RNA genome electrophoresis. Intervirology 1986; 26(3): 174–80. |  |  |  | |  | | X | |  | |  | | X | |  | | X | |  |  | | |  |  | |  | |  | |  | |  |  | | |  |  | |  | |  | |
| González FS, Sordo ME, Rowensztein G, Sabbag L, Roussos A, De Petre E, Garello M, Medei A, Bok K, Grinstein S, Gómez JA. [Rotavirus diarrhea Impact in a pediatric hospital of Buenos Aires]Medicina (B Aires). 1999;59(4):321-6. |  |  |  | |  | |  | |  | |  | |  | |  | |  | |  |  | | |  |  | |  | |  | |  | |  |  | | |  | X | |  | |  | |
| González GG, Liprandi F, Ludert JE. Molecular epidemiology of enteric viruses in children with sporadic gastroenteritis in Valencia, Venezuela. J Med Virol. 2011 Nov;83(11):1972-82. |  |  |  | |  | |  | |  | |  | |  | |  | |  | | X | X | | | X |  | |  | |  | |  | |  |  | | |  | X | |  | |  | |
| Gonzalez-Galan V, Sánchez-Fauqier A, Obando I, Montero V, Fernandez M, Torres MJ, Neth O, Aznar-Martin J. High prevalence of community-acquired norovirus gastroenteritis among hospitalized children: a prospective study. Clin Microbiol Infect. 2011 Dec;17(12):1895-9. |  |  |  | |  | |  | |  | |  | |  | |  | |  | | X |  | | | X | X | |  | |  | |  | |  |  | | |  | X | | X | |  | |
| Gorbachev EN, Verbov VN, Galko NV, Makarova NG, Vashukova SS. Immunoenzyme analysis in the diagnosis of human rotavirus gastroenteritis (methodological aspects). Vopr. Virusol. 1986; 31(6): 743–6. |  |  |  | |  | | X | |  | |  | | X | |  | | X | |  |  | | |  |  | |  | |  | |  | |  |  | | |  |  | |  | |  | |
| Gouvea VS, Dias GS, Aguiar EA, Pedro AR, Fichman ER, Chinem ES, Gomes SP, Domingues AL.Acute gastroenteritis in a pediatric hospital in rio de janeiro in pre- and post-rotavirus vaccination settings.Open Virol J. 2009 Apr 20;3:26-30. |  |  |  | |  | |  | |  | |  | |  | |  | |  | |  |  | | |  |  | |  | |  | |  | |  |  | | |  | X | |  | |  | |
| Grassi T, De Donno A, Guido M, Gabutti G. The epidemiology and disease burden of rotavirus infection in the Salento peninsula, Italy. Turk. J. Pediatr. 2008; 50(2): 132–6. |  |  |  | |  | | X | |  | |  | | X | |  | | X | |  |  | | |  |  | |  | |  | |  | |  |  | | |  |  | |  | |  | |
| Greenberg BL, Sack RB, Salazar-Lindo E, Budge E, Gutierrez M, Campos M, Visberg A, Leon-Barua R, Yi A, Maurutia D. Measles-associated diarrhea in hospitalized children in Lima, Peru: pathogenic agents and impact on growth. J. Infect. Dis. 1991; 163(3): 495–502. |  | X |  | | X | | X | |  | |  | | X | |  | | X | |  |  | | |  | X | |  | | X | |  | |  | X | | | X | X | |  | | X | |
| Griffiths FH, Steele AD, Alexander JJ. The molecular epidemiology of rotavirus-associated gastro-enteritis in the Transkei, southern Africa. Ann Trop Paediatr. 1992;12(3):259-64. |  |  |  | |  | |  | |  | |  | |  | |  | |  | |  |  | | |  |  | |  | |  | |  | |  |  | | |  | X | |  | |  | |
| Gudkov V, Virinskaya A, Zaytseva L. Status and ways of improvements of methods for detections of rotavirus infection in Republic of Belarus. Ministry of Health of the Republic of Minsk 1996; : 75–83. |  |  |  | |  | | X | |  | |  | |  | |  | |  | |  |  | | |  |  | |  | |  | |  | |  |  | | |  |  | |  | |  | |
| Gudkov VG, Virinskaya AS, Zaytseva LV. Status and ways of improvements of methods for detections of rotavirus infection in Republic of Belarus. Program and abstracts of the IX Congress of the Prophylactic Medicine Workers of Republic of Belarus on Modern Issues of Epidemiology and Surveillance of Infectious Diseases 1996; : 75–83. |  |  |  | |  | |  | |  | |  | | X | |  | | X | |  |  | | |  |  | |  | |  | |  | |  |  | | |  |  | |  | |  | |
| Guerra-Godínez JC, Larrosa-Haro A, Coello-Ramírez P, Tostado HRA, Rivera-Chávez E, Castillo de León YA, Bojórquez-Ramos M del C, Aguilar-Benavides S. Changing trends in prevalence, morbidity, and lethality in persistent diarrhea of infancy during the last decade in Mexico. Arch. Med. Res. 2003; 34(3): 209–13. | X | X |  | |  | |  | |  | |  | |  | | X | |  | |  |  | | |  |  | |  | |  | |  | |  |  | | |  |  | |  | |  | |
| Guerrero ML, Noel JS, Mitchell DK, Calva JJ, Morrow AL, Martínez J, Rosales G, Velázquez FR, Monroe SS, Glass RI, Pickering LK, Ruiz-Palacios GM. A prospective study of astrovirus diarrhea of infancy in Mexico City. Pediatr. Infect. Dis. J. 1998; 17(8): 723–7. |  |  |  | |  | | X | |  | |  | | X | |  | | X | |  |  | | |  |  | |  | |  | |  | |  |  | | |  |  | |  | |  | |
| Gusmão RH, Mascarenhas JD, Gabbay YB, Lins-Lainson Z, Ramos FL, Monteiro TA, Valente SA, Fagundes-Neto U, Linhares AC. Rotavirus subgroups, G serotypes, and electrophoretypes in cases of nosocomial infantile diarrhoea in Belém, Brazil. J. Trop. Pediatr. 1999; 45(2): 81–6. |  |  |  | | X | | X | |  | |  | | X | | X | | X | | X | X | | |  |  | |  | | X | | X | |  |  | | | X | X | | X | | X | |
| Hamedi Y, Safa O, Haidari M. Cryptosporidium infection in diarrheic children in southeastern Iran. Pediatr. Infect. Dis. J. 2005; 24(1): 86–8. |  |  |  | | X | |  | |  | |  | |  | |  | |  | |  |  | | |  |  | |  | |  | |  | |  |  | | |  |  | |  | |  | |
| Haq JA, Rahman KM. Campylobacter jejuni as a cause of acute diarrhoea in children: a study at an urban hospital in Bangladesh. J Trop Med Hyg. 1991 Feb;94(1):50-4. |  |  |  | |  | |  | |  | |  | |  | |  | |  | |  |  | | |  | X | |  | |  | | X | |  |  | | | X |  | |  | |  | |
| Haque R, Mondal D, Kirkpatrick BD, Akther S, Farr BM, Sack RB, Petri WA Jr. Epidemiologic and clinical characteristics of acute diarrhea with emphasis on Entamoeba histolytica infections in preschool children in an urban slum of Dhaka, Bangladesh. Am. J. Trop. Med. Hyg. 2003; 69(4): 398–405. |  |  | X | |  | | X | | X | | X | |  | |  | |  | |  |  | | |  |  | |  | |  | |  | |  |  | | |  |  | |  | |  | |
| Henry FJ, Udoy AS, Wanke CA, Aziz KM. Epidemiology of persistent diarrhea and etiologic agents in Mirzapur, Bangladesh. Acta Paediatr Suppl 1992; 381: 27–31. | X | X |  | |  | | X | |  | | X | | X | | X | | X | |  |  | | |  |  | |  | |  | |  | |  |  | | |  |  | |  | |  | |
| Hsu VP, Abdul Rahman HB, Wong SL, Ibrahim LH, Yusoff AF, Chan LG, Parashar U, Glass RI, Bresee J. Estimates of the burden of rotavirus disease in Malaysia. J Infect Dis. 2005 Sep 1;192 Suppl 1:S80-6. |  |  |  | |  | |  | |  | |  | |  | |  | |  | |  |  | | |  |  | |  | |  | |  | |  |  | | |  | X | |  | |  | |
| Hsu VP, Staat MA, Roberts N, Thieman C, Bernstein DI, Bresee J, Glass RI, Parashar UD. Use of active surveillance to validate international classification of diseases code estimates of rotavirus hospitalizations in children. Pediatrics. 2005 Jan;115(1):78-82. |  |  |  | |  | |  | |  | |  | |  | |  | |  | |  |  | | |  |  | |  | |  | |  | |  |  | | |  | X | |  | |  | |
| Isakbaeva ET, Musabaev E, Antil L, Rheingans R, Juraev R, Glass RI, Bresee JS. Rotavirus disease in Uzbekistan: cost-effectiveness of a new vaccine. Vaccine 2007; 25(2): 373–80. |  |  |  | |  | | X | |  | |  | | X | |  | | X | |  |  | | |  |  | |  | |  | |  | |  |  | | |  |  | |  | |  | |
| Islam SS, Shahid NS. Morbidity and mortality in a diarrhoeal diseases hospital in Bangladesh. Trans. R. Soc. Trop. Med. Hyg. 1986; 80(5): 748–52. |  |  |  | |  | |  | |  | |  | |  | | X | |  | |  |  | | |  |  | |  | |  | |  | |  |  | | |  |  | |  | |  | |
| Izzuddin Poo M, Lee WS. Admission to hospital with childhood acute gastroenteritis in Kuala Lumpur, Malaysia. Med. J. Malaysia. 2007; 62(3): 189–93. |  |  |  | |  | | X | |  | |  | | X | |  | | X | |  |  | | |  |  | |  | |  | |  | |  |  | | |  |  | |  | |  | |
| Jafari F, Garcia-Gil LJ, Salmanzadeh-Ahrabi S, Shokrzadeh L, Aslani MM, Pourhoseingholi MA, Derakhshan F, Zali MR. Diagnosis and prevalence of enteropathogenic bacteria in children less than 5 years of age with acute diarrhea in Tehran children's hospitals. J Infect. 2009 Jan;58(1):21-7. |  |  |  | |  | |  | |  | |  | |  | |  | |  | |  |  | | |  | X | |  | |  | |  | | X | X | | |  |  | | X | | X | |
| Jain V, Das BK, Bhan MK, Glass RI, Gentsch JR; Indian Strain Surveillance Collaborating Laboratories. Great diversity of group A rotavirus strains and high prevalence of mixed rotavirus infections in India. J Clin Microbiol. 2001 Oct;39(10):3524-9. |  |  |  | |  | |  | |  | |  | |  | |  | |  | |  |  | | |  |  | |  | |  | |  | |  |  | | |  | X | |  | |  | |
| Jenney A, Tikoduadua L, Buadromo E, Barnes G, Kirkwood CD, Boniface K, Bines J, Mulholland K, Russell F. The burden of hospitalised rotavirus infections in Fiji. Vaccine 2009; 27 Suppl 5: F108–11. |  |  |  | |  | | X | |  | |  | | X | |  | | X | |  |  | | |  |  | |  | |  | |  | |  |  | | |  | X | |  | |  | |
| Jin S, Kilgore PE, Holman RC, Clarke MJ, Gangarosa EJ, Glass RI. Trends in hospitalizations for diarrhea in United States children from 1979 through 1992: estimates of the morbidity associated with rotavirus. Pediatr Infect Dis J. 1996 May;15(5):397-404. |  |  |  | |  | |  | |  | |  | |  | |  | |  | |  |  | | |  |  | |  | |  | |  | |  |  | | |  |  | |  | |  | |
| Jin Y, Cheng WX, Yang XM, Jin M, Zhang Q, Xu ZQ, Yu JM, Zhu L, Yang SH, Liu N, Cui SX, Fang ZY, Duan ZJ. Viral agents associated with acute gastroenteritis in children hospitalized with diarrhea in Lanzhou, China. J Clin Virol. 2009 Mar;44(3):238-41. |  |  |  | |  | |  | |  | |  | |  | |  | |  | | X | X | | | X |  | |  | |  | |  | |  |  | | |  | X | |  | |  | |
| Jin Y, Ye X-H, Fang Z-Y, Li Y-N, Yang X-M, Dong Q-L, Huang X. Molecular epidemic features and variation of rotavirus among children with diarrhea in Lanzhou, China, 2001-2006. World J Pediatr 2008; 4(3): 197–201. |  |  |  | |  | | X | |  | |  | | X | |  | | X | |  |  | | |  |  | |  | |  | |  | |  |  | | |  |  | |  | |  | |
| Jindal N, Arora R, Bhushan B, Arora S. A study of infective aetiology of chronic diarrhoea in children in Amritsar. J Indian Med Assoc 1995; 93(5): 169–70. |  | X |  | |  | |  | |  | |  | |  | |  | |  | |  |  | | |  |  | |  | |  | |  | |  |  | | |  |  | |  | |  | |
| Jiraphongsa C, Bresee JS, Pongsuwanna Y, Kluabwang P, Poonawagul U, Arporntip P, Kanoksil M, Premsri N, Intusoma U. Epidemiology and burden of rotavirus diarrhea in Thailand: results of sentinel surveillance. J. Infect. Dis. 2005; 192 Suppl 1: S87–93. |  |  |  | |  | | X | |  | |  | | X | |  | | X | |  |  | | |  |  | |  | |  | |  | |  |  | | |  | X | |  | |  | |
| Jirapinyo P, Ruangsiri K, Tesjaroen S, Limsathayourat N, Sripiangjan J, Yoolek A, Junnoo V High prevalence of Cryptosporidium in young children with prolonged diarrhea.Southeast Asian J Trop Med Public Health. 1993 Dec;24(4):730-3. |  |  |  | |  | |  | |  | |  | |  | |  | |  | |  |  | | |  |  | |  | | X | |  | |  |  | | |  |  | |  | |  | |
| Junquera CG, de Baranda CS, Mialdea OG, Serrano EB, Sánchez-Fauquier A. Prevalence and clinical characteristics of norovirus gastroenteritis among hospitalized children in Spain. Pediatr Infect Dis J. 2009 Jul;28(7):604-7. |  |  |  | |  | |  | |  | |  | |  | |  | |  | | X | X | | | X | X | |  | |  | |  | |  |  | | |  | X | | X | | X | |
| Kamiya H, Nakano T, Kamiya H, Yui A, Taniguchi K, Parashar U; Rotavirus Epidemiology Study Group.Rotavirus-associated acute gastroenteritis hospitalizations among Japanese children aged <5 years: active rotavirus surveillance in Mie Prefecture, Japan.Jpn J Infect Dis. 2011;64(6):482-7. |  |  |  | |  | |  | |  | |  | |  | |  | |  | |  |  | | |  |  | |  | |  | |  | |  |  | | |  | X | |  | |  | |
| Kang G, Arora R, Chitambar SD, Deshpande J, Gupte MD, Kulkarni M, Naik TN, Mukherji D, Venkatasubramaniam S, Gentsch JR, Glass RI, Parashar UD. Multicenter, hospital-based surveillance of rotavirus disease and strains among indian children aged <5 years. J. Infect. Dis. 2009; 200 Suppl 1: S147–153. |  |  |  | |  | | X | |  | |  | | X | |  | | X | |  |  | | |  |  | |  | |  | |  | |  |  | | |  | X | |  | |  | |
| Katouli M, Jaafari A, Farhoudi-Moghaddam AA, Ketabi GR. Aetiological studies of diarrhoeal diseases in infants and young children in Iran. J Trop Med Hyg. 1990 Feb;93(1):22-7. |  |  |  | |  | |  | |  | |  | |  | |  | |  | |  |  | | |  |  | |  | |  | |  | |  | X | | |  |  | | X | | X | |
| Kele B, Abrok MP, Deak J.Sporadic norovirus infections among hospitalized and non-hospitalized 0-3-year-old infants.Scand J Infect Dis. 2009;41(1):67-9. |  |  |  | |  | |  | |  | |  | |  | |  | |  | |  |  | | |  |  | |  | |  | |  | |  |  | | |  |  | |  | |  | |
| Kelkar SD, Ayachit VL. Circulation of group A rotavirus subgroups and serotypes in Pune, India, 1990-1997. J Health Popul Nutr. 2000 Dec;18(3):163-70. |  |  |  | |  | |  | |  | |  | |  | |  | |  | |  |  | | |  |  | |  | |  | |  | |  |  | | |  | X | |  | |  | |
| Kelkar SD, Purohit SG, Simha KV. Prevalence of rotavirus diarrhoea among hospitalized children in Pune, India. Indian J Med Res. 1999 Apr;109:131-5. |  |  |  | |  | |  | |  | |  | |  | |  | |  | |  |  | | |  |  | |  | |  | |  | |  |  | | |  | X | |  | |  | |
| Khamrin P, Maneekarn N, Malasao R, Nguyen TA, Ishida S, Okitsu S, Ushijima H. Genotypic linkages of VP4, VP6, VP7, NSP4, NSP5 genes of rotaviruses circulating among children with acute gastroenteritis in Thailand. Infect. Genet. Evol. 2010; 10(4): 467–72. |  |  |  | |  | | X | |  | |  | | X | |  | | X | |  |  | | |  |  | |  | |  | |  | |  |  | | |  |  | |  | |  | |
| Khamrin P, Peerakome S, Tonusin S, Malasao R, Okitsu S, Mizuguchi M, Ushijima H, Maneekarn N. Changing pattern of rotavirus G genotype distribution in Chiang Mai, Thailand from 2002 to 2004: decline of G9 and reemergence of G1 and G2. J. Med. Virol. 2007; 79(11): 1775–82. |  |  |  | |  | | X | |  | |  | | X | |  | | X | |  |  | | |  |  | |  | |  | |  | |  |  | | |  |  | |  | |  | |
| Khan WA, Rogers KA, Karim MM, Ahmed S, Hibberd PL, Calderwood SB, et al. Cryptosporidiosis among Bangladeshi children with diarrhea: a prospective, matched, case-control study of clinical features, epidemiology and systemic antibody responses. Am J Trop Med Hyg. 2004 Oct;71(4):412-9. |  |  |  | |  | |  | |  | |  | |  | |  | |  | |  |  | | |  |  | |  | | X | |  | |  |  | | |  |  | |  | |  | |
| Khananurak K, Vutithanachot V, Simakachorn N, Theamboonlers A, Chongsrisawat V, Poovorawan Y. Prevalence and phylogenetic analysis of rotavirus genotypes in Thailand between 2007 and 2009. Infect Genet Evol. 2010 May;10(4):537-45. |  |  |  | |  | |  | |  | |  | |  | |  | |  | |  |  | | |  |  | |  | |  | |  | |  |  | | |  | X | |  | |  | |
| Kheyami AM, Nakagomi T, Nakagomi O, Dove W, Hart CA, Cunliffe NA. Molecular epidemiology of rotavirus diarrhea among children in Saudi Arabia: first detection of G9 and G12 strains. J. Clin. Microbiol. 2008; 46(4): 1185–91. |  |  |  | |  | | X | |  | |  | | X | |  | | X | |  |  | | |  |  | |  | |  | |  | |  |  | | |  |  | |  | |  | |
| Kim JS, Kang JO, Cho SC, Jang YT, Min SA, Park TH, et al. Epidemiological profile of rotavirus infection in the Republic of Korea: results from prospective surveillance in the Jeongeub District, 1 July 2002 through 30 June 2004. J Infect Dis. 2005 Sep 1;192 Suppl 1:S49-56. |  |  |  | |  | |  | |  | |  | |  | |  | |  | |  |  | | |  |  | |  | |  | |  | |  |  | | |  | X | |  | |  | |
| Kim KH, Yang JM, Joo SI, Cho YG, Glass RI, Cho YJ. Importance of rotavirus and adenovirus types 40 and 41 in acute gastroenteritis in Korean children. J Clin Microbiol 1990 Oct;28(10):2279-84. |  |  |  | |  | |  | |  | |  | |  | |  | |  | |  |  | | |  |  | |  | |  | |  | |  |  | | |  | X | |  | |  | |
| Komaid JA, De Caillou SL, Suárez AM, De Castagnaro NR. Electrophoretic types of rotavirus RNA during a 4-yr study of gastroenteritis in Tucumán. Rev. Argent. Microbiol. 1990; 22(3): 123–9. |  |  |  | |  | | X | |  | |  | | X | |  | | X | |  |  | | |  |  | |  | |  | |  | |  |  | | |  |  | |  | |  | |
| Kurugöl Z, Geylani S, Karaca Y, Umay F, Erensoy S, Vardar F, Bak M, Yaprak I, Ozkinay F, Ozkinay C. Rotavirus gastroenteritis among children under five years of age in Izmir, Turkey. Turk J Pediatr. 2003 Oct-Dec;45(4):290-4. |  |  |  | |  | |  | |  | |  | |  | |  | |  | |  |  | | |  |  | |  | |  | |  | |  |  | | |  | X | |  | |  | |
| Latipov R, Utegenova E, Kuatbayeva A, Kasymbekova K, Abdykarimov S, Juraev R, Ismailov U, Flem E. Epidemiology and burden of rotavirus disease in Central Asia. Int J Infect Dis. 2011 Jul;15(7):e464-9. |  |  |  | |  | |  | |  | |  | |  | |  | |  | |  |  | | |  |  | |  | |  | |  | |  |  | | |  | X | |  | |  | |
| Lee SY, Hong SK, Lee SG, Suh CI, Park SW, Lee JH, Kim JH, Kim DS, Kim HM, Jang YT, Ma SH, Kim SY, Sohn YS, Kang JH, Paik SY. Human rotavirus genotypes in hospitalized children, South Korea, April 2005 to March 2007. Vaccine 2009; 27 Suppl 5: F97–101. |  |  |  | |  | | X | |  | |  | | X | |  | | X | |  |  | | |  |  | |  | |  | |  | |  |  | | |  | X | |  | |  | |
| Lerman Y, Slepon R, Cohen D. Epidemiology of acute diarrheal diseases in children in a high standard of living rural settlement in Israel. Pediatr. Infect. Dis. J. 1994; 13(2): 116–22. |  | X |  | |  | |  | | X | |  | |  | | X | |  | |  |  | | |  |  | |  | |  | |  | |  |  | | |  |  | |  | |  | |
| Lesmana M, Subekti DS, Tjaniadi P, Simanjuntak CH, Punjabi NH, Campbell JR, Oyofo BA. Spectrum of vibrio species associated with acute diarrhea in North Jakarta, Indonesia Diagn Microbiol Infect Dis. 2002 Jun;43(2):91-7. |  |  |  | |  | |  | |  | |  | |  | |  | |  | |  |  | | |  |  | | X | |  | |  | |  |  | | |  |  | |  | |  | |
| Levidiotou S, Gartzonika C, Papaventsis D, Christaki C, Priavali E, Zotos N, Kapsali E, Vrioni G. Viral agents of acute gastroenteritis in hospitalized children in Greece. Clin. Microbiol. Infect. 2009; 15(6): 596–8. |  |  |  | |  | | X | |  | |  | | X | |  | | X | | X | X | | | X | X | |  | | X | | X | |  |  | | | X | X | | X | | X | |
| Li DD, Liu N, Yu JM, Zhang Q, Cui SX, Zhang DL, Yang SH, Cao DJ, Xu ZQ, Duan ZJ. Molecular epidemiology of G9 rotavirus strains in children with diarrhoea hospitalized in Mainland China from January 2006 to December 2007. Vaccine. 2009 Nov 20;27 Suppl 5:F40-5. |  |  |  | |  | |  | |  | |  | |  | |  | |  | |  |  | | |  |  | |  | |  | |  | |  |  | | |  | X | |  | |  | |
| Lim YS, Ngan CC, Tay L. Enteropathogenic Escherichia coli as a cause of diarrhoea among children in Singapore. J Trop Med Hyg 1992 Oct;95(5):339-42. |  |  |  | |  | |  | |  | |  | |  | |  | |  | |  |  | | |  | X | |  | |  | |  | |  |  | | |  |  | | X | | X | |
| Lim YS, Tay L. A one-year study of enteric Campylobacter infections in Singapore. J Trop Med Hyg 1992; 95(2): 119–23. |  | X | X | |  | |  | |  | |  | |  | | X | |  | |  |  | | |  | X | |  | |  | |  | |  |  | | |  |  | |  | |  | |
| Lima AA, Guerrant RL. Persistent diarrhea in children: epidemiology, risk factors, pathophysiology, nutritional impact, and management. Epidemiol Rev 1992; 14: 222–42. |  |  |  | |  | | X | |  | |  | | X | |  | | X | |  |  | | |  |  | |  | |  | |  | |  |  | | |  |  | |  | |  | |
| Lima AA, Moore SR, Barboza MS Jr, Soares AM, Schleupner MA, Newman RD, Sears CL, Nataro JP, Fedorko DP, Wuhib T, Schorling JB, Guerrant RL. Persistent diarrhea signals a critical period of increased diarrhea burdens and nutritional shortfalls: a prospective cohort study among children in northeastern Brazil. J. Infect. Dis. 2000; 181(5): 1643–51. |  |  | X | | X | | X | | X | | X | | X | | X | | X | |  |  | | |  |  | |  | |  | |  | |  |  | | |  |  | |  | |  | |
| Loening WE, Coovadia YM, Van den Ende J. Aetiological factors of infantile diarrhoea: a community-based study. Ann Trop Paediatr 1989; 9(4): 248–55. |  |  | X | |  | | X | | X | | X | |  | |  | |  | |  |  | | |  |  | |  | |  | |  | |  |  | | |  |  | |  | |  | |
| Lombardi GH, Roseto AM, Stamboulian D, Oro JG. Letter: Virus of infantile gastroenteritis in argentina. Lancet 1975; 2(7948): 1311. |  |  |  | |  | | X | |  | |  | | X | |  | | X | |  |  | | |  |  | |  | |  | |  | |  |  | | |  |  | |  | |  | |
| Lorrot M, Bon F, El Hajje MJ, Aho S, Wolfer M, Giraudon H, Kaplon J, Marc E, Raymond J, Lebon P, Pothier P, Gendrel D. Epidemiology and clinical features of gastroenteritis in hospitalised children: prospective survey during a 2-year period in a Parisian hospital, France. Eur J Clin Microbiol Infect Dis. 2011 Mar;30(3):361-8. |  |  |  | |  | |  | |  | |  | |  | |  | |  | | X | X | | | X |  | |  | |  | |  | |  |  | | |  | X | |  | |  | |
| Luz CRNE da, Mascarenhas JDP, Gabbay YB, Motta ARB, Lima TVR, Soares L da S, Linhares AC. Rotavirus serotypes and electropherotypes identified among hospitalised children in São Luís, Maranhão, Brazil. Rev. Inst. Med. Trop. Sao Paulo. 2005; 47(5): 287–93. |  |  |  | |  | | X | |  | |  | | X | |  | | X | |  |  | | |  |  | |  | |  | |  | |  |  | | |  |  | |  | |  | |
| Mahalanabis D, Alam AN, Rahman N, Hasnat A. Prognostic indicators and risk factors for increased duration of acute diarrhoea and for persistent diarrhoea in children. Int J Epidemiol 1991; 20(4): 1064–72. |  | X |  | |  | | X | | X | |  | | X | |  | | X | |  |  | | |  |  | |  | |  | |  | |  |  | | |  |  | |  | |  | |
| Maldonado A, Bastardo J. Prevalencia de subgrupos,serotipos y electroferotipos de rotavirus humanos en Cumaná, Venezuela / Prevalence of subgroups and electropherotypes of humans rotavirus in Cumaná, Venezuela. Invest Clin. 1998 Sep;39(3):39-51. |  |  |  | |  | |  | |  | |  | |  | |  | |  | |  |  | | |  |  | |  | |  | |  | |  |  | | |  | X | |  | |  | |
| Mandomando IM, Macete EV, Ruiz J, Sanz S, Abacassamo F, Vallès X, Sacarlal J, Navia MM, Vila J, Alonso PL, Gascon J. Etiology of diarrhea in children younger than 5 years of age admitted in a rural hospital of southern Mozambique. Am J Trop Med Hyg. 2007 Mar;76(3):522-7. |  |  |  | |  | |  | |  | |  | |  | |  | |  | |  |  | | |  |  | |  | |  | |  | |  |  | | |  |  | |  | |  | |
| Manna B, Niyogi SK, Bhattacharya MK, Sur D, Bhattacharya SK. Observations from diarrhoea surveillance support the use of cholera vaccination in endemic areas. Int J Infect Dis. 2005 Mar;9(2):117-9. |  |  |  | |  | |  | |  | |  | |  | |  | |  | |  |  | | |  |  | | X | |  | |  | |  |  | | |  |  | |  | |  | |
| Mapaseka SL, Dewar JB, Van der Merwe L, Geyer A, Tumbo J, Zweygarth M, Bos P, Esona MD, Steele AD, Sommerfelt H. Prospective hospital-based surveillance to estimate rotavirus disease burden in the Gauteng and North West Province of South Africa during 2003-2005. J. Infect. Dis. 2010; 202 Suppl: S131–138. |  |  |  | |  | | X | |  | |  | | X | |  | | X | |  |  | | |  |  | |  | |  | |  | |  |  | | |  |  | |  | |  | |
| Mathan VI, Rajan DP. The prevalence of bacterial intestinal pathogens in a healthy rural population in southern India. J. Med. Microbiol. 1986; 22(2): 93–6. |  |  |  | |  | |  | |  | | X | |  | | X | |  | |  |  | | |  |  | |  | |  | |  | |  |  | | |  |  | |  | |  | |
| Matson DO, Abdel-Messih IA, Schlett CD, Bok K, Wienkopff T, Wierzba TF, Sanders JW, Frenck RW Jr. Rotavirus genotypes among hospitalized children in Egypt, 2000-2002. J. Infect. Dis. 2010; 202 Suppl: S263–265. |  |  |  | |  | | X | |  | |  | | X | |  | | X | |  |  | | |  |  | |  | |  | |  | |  |  | | |  |  | |  | |  | |
| Mattison K, Sebunya TK, Shukla A, Noliwe LN, Bidawid S. Molecular detection and characterization of noroviruses from children in Botswana. J Med Virol. 2010 Feb;82(2):321-4. |  |  |  | |  | |  | |  | |  | |  | |  | |  | |  |  | | |  |  | |  | |  | |  | |  |  | | |  |  | |  | |  | |
| McIver CJ, Hansman G, White P, Doultree JC, Catton M, Rawlinson WD. Diagnosis of enteric pathogens in children with gastroenteritis. Pathology. 2001 Aug;33(3):353-8. |  |  |  | |  | |  | |  | |  | |  | |  | |  | | X | X | | |  | X | |  | |  | |  | |  |  | | |  | X | | X | |  | |
| Mladenova Z, Korsun N, Geonova T, Iturriza-Gómara M. Molecular epidemiology of rotaviruses in Bulgaria: annual shift of the predominant genotype. Eur. J. Clin. Microbiol. Infect. Dis. 2010; 29(5): 555–62. |  |  |  | |  | | X | |  | |  | | X | |  | | X | |  |  | | |  |  | |  | |  | |  | |  |  | | |  | X | |  | |  | |
| Modaress S, Rahbarimanesh AA, Edalat R, Sohrabi A, Modarres S, Gomari H, Motamedirad M, Sayari AA. Human rotavirus genotypes detection among hospitalized children, a study in Tehran, Iran. Arch Iran Med 2011; 14(1): 39–45. |  |  |  | |  | | X | |  | |  | | X | |  | | X | |  |  | | |  |  | |  | |  | |  | |  |  | | |  |  | |  | |  | |
| Moe K, Hummelman EG, Oo WM, Lwin T, Htwe TT. Hospital-based surveillance for rotavirus diarrhea in children in Yangon, Myanmar. J Infect Dis. 2005 Sep 1;192 Suppl 1:S111-3. |  |  |  | |  | |  | |  | |  | |  | |  | |  | |  |  | | |  |  | |  | |  | |  | |  |  | | |  | X | |  | |  | |
| Moe K, Thu HM, Oo WM, Aye KM, Shwe TT, Mar W, Kirkwood CD. Genotyping of rotavirus isolates collected from children less than 5 years of age admitted for diarrhoea at the Yangon Children’s Hospital, Myanmar. Vaccine 2009; 27 Suppl 5: F89–92. |  |  |  | |  | | X | |  | |  | | X | |  | | X | |  |  | | |  |  | |  | |  | |  | |  |  | | |  |  | |  | |  | |
| Mpabalwani M, Oshitani H, Kasolo F, Mizuta K, Luo N, Matsubayashi N, Bhat G, Suzuki H, Numazaki Y. Rotavirus gastro-enteritis in hospitalized children with acute diarrhoea in Zambia. Ann Trop Paediatr. 1995;15(1):39-43. |  |  |  | |  | |  | |  | |  | |  | |  | |  | |  |  | | |  |  | |  | |  | |  | |  |  | | |  | X | |  | |  | |
| Mrukowicz JZ, Krobicka B, Duplaga M, Kowalska-Duplaga K, Domañski J, Szajewska H, Kantecki M, Iwañczak F, Pytrus T. Epidemiology and impact of rotavirus diarrhoea in Poland. Acta Paediatr Suppl. 1999 Jan;88(426):53-60. |  |  |  | |  | |  | |  | |  | |  | |  | |  | |  |  | | |  |  | |  | |  | |  | |  |  | | |  | X | |  | |  | |
| Muchinik GR, Grinstein S, Plaza A. Rotavirus infection in children hospitalized for diarrhoea in Argentina. Ann Trop Paediatr 1981; 1(3): 167–73. |  |  |  | |  | | X | |  | |  | | X | |  | | X | |  |  | | |  |  | |  | |  | |  | |  |  | | |  |  | |  | |  | |
| Muhsen K, Shulman L, Rubinstein U, Kasem E, Kremer A, Goren S, Zilberstein I, Chodick G, Ephros M, Cohen D. Incidence, characteristics, and economic burden of rotavirus gastroenteritis associated with hospitalization of israeli children <5 years of age, 2007-2008. J. Infect. Dis. 2009; 200 Suppl 1: S254–63. |  | X |  | |  | | X | |  | |  | | X | | X | | X | |  |  | | |  |  | |  | |  | |  | |  |  | | |  |  | |  | |  | |
| Mukherjee AK, Chowdhury P, Bhattacharya MK, Ghosh M, Rajendran K, Ganguly S. Hospital-based surveillance of enteric parasites in Kolkata. BMC Res Notes. 2009 Jun 19;2:110. |  |  |  | |  | |  | |  | |  | |  | |  | |  | |  |  | | |  |  | |  | |  | |  | |  |  | | | X |  | |  | |  | |
| Munford V, Gilio AE, de Souza EC, Cardoso DM, Cardoso DD, Borges AM, Costa PS, Melgaço IA, Rosa H, Carvalho PR, Goldani MZ, Moreira ED Jr, Santana C, El Khoury A, Ikedo F, Rácz ML. Rotavirus gastroenteritis in children in 4 regions in Brazil: a hospital-based surveillance study.J Infect Dis. 2009 Nov 1;200 Suppl 1:S106-13. |  |  |  | |  | |  | |  | |  | |  | |  | |  | |  |  | | |  |  | |  | |  | |  | |  |  | | |  | X | |  | |  | |
| Mwenda JM, Ntoto KM, Abebe A, Enweronu-Laryea C, Amina I, Mchomvu J, Kisakye A, Mpabalwani EM, Pazvakavambwa I, Armah GE, Seheri LM, Kiulia NM, Page N, Widdowson M-A, Steele AD. Burden and epidemiology of rotavirus diarrhea in selected African countries: preliminary results from the African Rotavirus Surveillance Network. J. Infect. Dis. 2010; 202 Suppl: S5–S11. |  |  |  | |  | | X | |  | |  | | X | |  | | X | |  |  | | |  |  | |  | |  | |  | |  |  | | |  |  | |  | |  | |
| Nacro B, Bonkoungou P, Nagalo K, Tall FR, Curtis V. [Clinical profile of cryptosporidiosis in a pediatric hospital environment in Burkina Faso]. Med Trop (Mars). 1998;58(1):47-50. |  |  |  | |  | |  | |  | |  | |  | |  | |  | |  |  | | |  |  | |  | | X | |  | |  |  | | |  |  | |  | |  | |
| Nafi O. Rotavirus gastroenteritis among children aged under 5 years in Al Karak, Jordan.East Mediterr Health J. 2010 Oct;16(10):1064-9. |  |  |  | |  | |  | |  | |  | |  | |  | |  | |  |  | | |  |  | |  | |  | |  | |  |  | | |  | X | |  | |  | |
| Nagamani K, Pavuluri PRR, Gyaneshwari M, Prasanthi K, Rao MIS, Saxena NK. Molecular characterisation of Cryptosporidium: an emerging parasite. Indian J Med Microbiol 2007; 25(2): 133–6. |  |  |  | | X | |  | |  | |  | |  | |  | |  | |  |  | | |  |  | |  | |  | |  | |  |  | | |  |  | |  | |  | |
| Nair GB, Ramamurthy T, Bhattacharya MK, Krishnan T, Ganguly S, Saha DR, Rajendran K, Manna B, Ghosh M, Okamoto K, Takeda Y. Emerging trends in the etiology of enteric pathogens as evidenced from an active surveillance of hospitalized diarrhoeal patients in Kolkata, India. Gut Pathog. 2010 Jun 5;2(1):4. |  |  |  | |  | |  | |  | |  | |  | |  | |  | | X | X | | | X | X | | X | |  | | X | | X | X | | | X | X | | X | | X | |
| Nakagomi T, Nakagomi O, Takahashi Y, Enoki M, Suzuki T, Kilgore PE. Incidence and burden of rotavirus gastroenteritis in Japan, as estimated from a prospective sentinel hospital study. J Infect Dis. 2005 Sep 1;192 Suppl 1:S106-10. |  |  |  | |  | |  | |  | |  | |  | |  | |  | |  |  | | |  |  | |  | |  | |  | |  |  | | |  | X | |  | |  | |
| Nath G, Choudhury A, Shukla BN, Singh TB, Reddy DC. Significance of Cryptosporidium in acute diarrhoea in North-Eastern India. J. Med. Microbiol. 1999; 48(6): 523–6. | X | X | X | | X | |  | | X | | X | |  | | X | |  | |  |  | | |  | X | |  | | X | | X | | X | X | | | X | X | | X | | X | |
| Nelson EA, Tam JS, Bresee JS, Poon KH, Ng CH, Ip KS, Mast TC, Chan PK, Parashar UD, Fok TF, Glass RI. Estimates of rotavirus disease burden in Hong Kong: hospital-based surveillance. J Infect Dis. 2005 Sep 1;192 Suppl 1:S71-9. |  |  |  | |  | |  | |  | |  | |  | |  | |  | |  |  | | |  |  | |  | |  | |  | |  |  | | |  | X | |  | |  | |
| Newman RD, Sears CL, Moore SR, Nataro JP, Wuhib T, Agnew DA, Guerrant RL, Lima AA. Longitudinal study of Cryptosporidium infection in children in northeastern Brazil. Brazil. J. Infect. Dis. 1999; 180(1): 167–75. |  |  |  | | X | |  | |  | |  | |  | |  | |  | |  |  | | |  |  | |  | | X | |  | |  |  | | |  |  | |  | |  | |
| Ngan PK, Khanh NG, Tuong CV, Quy PP, Anh DN, Thuy HT. Persistent diarrhea in Vietnamese children: a preliminary report. Acta Paediatr 1992; 81 Suppl 381: 124–6. |  |  |  | |  | | X | | X | | X | | X | |  | | X | |  |  | | |  |  | |  | |  | |  | |  |  | | |  |  | |  | |  | |
| Ngo TC, Nguyen BM, Dang DA, Nguyen HT, Nguyen TT, Tran VN, Vu TT, Ogino M, Alam MM, Nakagomi T, Nakagomi O, Yamashiro T. Molecular epidemiology of rotavirus diarrhoea among children in Haiphong, Vietnam: the emergence of G3 rotavirus. Vaccine. 2009 Nov 20;27 Suppl 5:F75-80. |  |  |  | |  | |  | |  | |  | |  | |  | |  | |  |  | | |  |  | |  | |  | |  | |  |  | | |  | X | |  | |  | |
| Nguyen VM, Nguyen VT, Huynh PL, Dang DT, Nguyen TH, Phan VT, Nguyen TL, Le TL, Ivanoff B, Gentsch JR, Glass RI, Vietnam Rotavirus Surveillance Network. The epidemiology and disease burden of rotavirus in Vietnam: sentinell surveillance at 6 hospitals. J Infect Dis. 2001 Jun 15;183(12):1707-12. Epub 2001 May 16. |  |  |  | |  | |  | |  | |  | |  | |  | |  | |  |  | | |  |  | |  | |  | |  | |  |  | | |  | X | |  | |  | |
| Novikova NA, Epifanova NV, Fardzinova VF, Chechueva LI, Fevraleva EL, Kashnikov AI. The detection of atypical human rotaviruses and their characteristics. Immunobiol 1994; 2: 103–6. |  |  |  | |  | | X | |  | |  | | X | |  | | X | |  |  | | |  |  | |  | |  | |  | |  |  | | |  |  | |  | |  | |
| Novikova NA, Epifanova NV, Makeeva LV, Kashnikov AI. Longitudinal observations on the circulation of rotaviruses in Nizhny Novgorod using molecular-genetic methods. Zh. Mikrobiol. Epidemiol. Immunobiol. 1998; 3: 21–3. |  |  |  | |  | | X | |  | |  | | X | |  | | X | |  |  | | |  |  | |  | |  | |  | |  |  | | |  |  | |  | |  | |
| Novikova NA, Fedorova OF, Epifanova NV, Chuprova AB. G [P] type profiles of group A human rotavirus and their distribution in Nizhni Novgorod and Dzerzhinsk in 1997-2005. Vopr. Virusol. 2007; 52(3): 19–23. |  |  |  | |  | | X | |  | |  | | X | |  | | X | |  |  | | |  |  | |  | |  | |  | |  |  | | |  |  | |  | |  | |
| Nyambat B, Gantuya S, Batuwanthudawe R, Wijesinghe PR, Abeysinghe N, Galagoda G, Kirkwood C, Bogdanovic-Sakran N, Kang JO, Kilgore PE. Epidemiology of rotavirus diarrhea in mongolia and sri lanka, march 2005-february 2007. J. Infect. Dis. 2009; 200 Suppl 1: S160–166. |  |  |  | |  | | X | |  | |  | |  | |  | |  | |  |  | | |  |  | |  | |  | |  | |  |  | | |  | X | |  | |  | |
| Nyambat B, Meng CY, Vansith K, Vuthy U, Rin E, Kirkwood C, Bogdanovic-Sakran N, Kilgore PE. Hospital-based surveillance for rotavirus diarrhoea in Phnom Penh, Cambodia, March 2005 through February 2007. Vaccine 2009; 27 Suppl 5: F81–84. |  |  |  | |  | | X | |  | |  | | X | |  | | X | |  |  | | |  |  | |  | |  | |  | |  |  | | |  | X | |  | |  | |
| O'Ryan M, Pérez-Schael I, Mamani N, Peña A, Salinas B, González G, González F, Matson DO, Gómez J. Rotavirus-associated medical visits and hospitalizations in South America: a prospective study at three large sentinel hospitals. Pediatr Infect Dis J. 2001 Jul;20(7):685-93. |  |  |  | |  | |  | |  | |  | |  | |  | |  | |  |  | | |  |  | |  | |  | |  | |  |  | | |  | X | |  | |  | |
| O'Ryan ML, Peña A, Vergara R, Díaz J, Mamani N, Cortés H, Lucero Y, Vidal R, Osorio G, Santolaya ME, Hermosilla G, Prado VJ. Prospective characterization of norovirus compared with rotavirus acute diarrhea episodes in chilean children. Pediatr Infect Dis J. 2010 Sep;29(9):855-9. |  |  |  | |  | |  | |  | |  | |  | |  | |  | |  |  | | |  |  | |  | |  | |  | |  |  | | |  | X | |  | |  | |
| O’Ryan M, Pérez-Schael I, Mamani N, Peña A, Salinas B, González G, González F, Matson DO, Gómez J. Rotavirus-associated medical visits and hospitalizations in South America: a prospective study at three large sentinel hospitals. Pediatr. Infect. Dis. J. 2001; 20(7): 685–93. |  |  |  | |  | | X | |  | |  | | X | |  | | X | |  |  | | |  |  | |  | |  | |  | |  |  | | |  |  | |  | |  | |
| Obeid OE. Characterization of human rotavirus subgroups and serotypes in children under five with acute gastroenteritis in a Saudi Hospital. J Family Community Med. 2011 Jan;18(1):22-5. |  |  |  | |  | |  | |  | |  | |  | |  | |  | |  |  | | |  |  | |  | |  | |  | |  |  | | |  |  | |  | |  | |
| Oberhelman RA, Gilman RH, Sheen P, Taylor DN, Black RE, Cabrera L, Lescano AG, Meza R, Madico G. A placebo-controlled trial of Lactobacillus GG to prevent diarrhea in undernourished Peruvian children. J. Pediatr. 1999; 134(1): 15–20. |  | X |  | |  | | X | |  | |  | | X | | X | | X | | X |  | | | X |  | |  | |  | |  | |  |  | | |  | X | |  | |  | |
| Oberle MW, Merson MH, Islam MS, Rahman AS, Huber DH, Curlin G. Diarrhoeal disease in Bangladesh: epidemiology, mortality averted and costs at a rural treatment centre. Int J Epidemiol 1980; 9(4): 341–8. |  |  |  | |  | |  | |  | |  | |  | | X | |  | |  |  | | |  |  | |  | |  | |  | |  |  | | |  | X | |  | |  | |
| Oldak E, Sulik A, Rozkiewicz D, Liwoch-Nienartowicz N. Norovirus infections in children under 5 years of age hospitalized due to the acute viral gastroenteritis in northeastern Poland. Eur J Clin Microbiol Infect Dis. 2012 Apr;31(4):417-22. |  |  |  | |  | |  | |  | |  | |  | |  | |  | |  |  | | | X |  | |  | |  | |  | |  |  | | |  | X | |  | |  | |
| Oyofo BA, Subekti D, Tjaniadi P, Machpud N, Komalarini S, Setiawan B, Simanjuntak C, Punjabi N, Corwin AL, Wasfy M, Campbell JR, Lesmana M. Enteropathogens associated with acute diarrhea in community and hospital patients in Jakarta, Indonesia. FEMS Immunol Med Microbiol. 2002 Oct 11;34(2):139-46. |  |  |  | |  | |  | |  | |  | |  | |  | |  | |  |  | | |  | X | | X | |  | |  | |  |  | | |  |  | | X | | X | |
| Ozdemir S, Delialio?lu N, Emekda? G. Investigation of rotavirus, adenovirus and astrovirus frequencies in children with acute gastroenteritis and evaluation of epidemiological features. Mikrobiyol Bul 2010; 44(4): 571–8. |  |  |  | |  | | X | |  | |  | | X | |  | | X | |  |  | | |  |  | |  | |  | |  | |  |  | | |  |  | |  | |  | |
| Parra GI, Bok K, Martinez V, Russomando G, Gomez J. Molecular characterization and genetic variation of the VP7 gene of human rotaviruses isolated in Paraguay. J Med Virol. 2005 Dec;77(4):579-86. |  |  |  | |  | |  | |  | |  | |  | |  | |  | |  |  | | |  |  | |  | |  | |  | |  |  | | |  | X | |  | |  | |
| Pazzaglia G, Sack RB, Salazar E, Yi A, Chea E, Leon-Barua R, Guerrero CE, Palomino J. High frequency of coinfecting enteropathogens in Aeromonas-associated diarrhea of hospitalized Peruvian infants. J. Clin. Microbiol. 1991; 29(6): 1151–6. |  | X | X | |  | | X | | X | | X | | X | | X | | X | |  |  | | |  | X | |  | | X | | X | |  | X | | | X | X | | X | | X | |
| Penny ME, Paredes P, Brown KH, Laughan B, Smith H. Lack of a role of the duodenal microflora in pathogenesis of persistent diarrhea and diarrhea-related malabsorption in Peruvian children. Pediatr Infect Dis J. 1990 Jul;9(7):479-87. |  |  |  | |  | |  | |  | |  | |  | |  | |  | |  |  | | |  | X | |  | | X | | X | | X | X | | | X | X | | X | | X | |
| Phukan AC, Patgiri DK, Mahanta J. Rotavirus associated acute diarrhoea in hospitalized children in Dibrugarh, north-east India. Indian J Pathol Microbiol. 2003 Apr;46(2):274-8. |  |  |  | |  | |  | |  | |  | |  | |  | |  | |  |  | | |  |  | |  | |  | |  | |  |  | | |  | X | |  | |  | |
| Podkolzin AT, Fenske EB, Abramycheva NI, Shipulin GA, Bitieva RL, Sagalova OI, Mazepa VN, Ivanova GI, Semena AV, Tagirova ZG, Ivanova VV, Molochny? VP, Ivolgina AV, Maleev VV, Pokrovski? VI. Season and age related structure of acute intestinal infections morbidity in the Russian Federation. Ter. Arkh. 2007; 79(11): 10–6. |  |  |  | |  | | X | |  | |  | |  | |  | |  | |  |  | | |  |  | |  | |  | |  | |  |  | | |  |  | |  | |  | |
| Podkolzin AT, Fenske EB, Abramycheva NY, Shipulin GA, Sagalova OI, Mazepa VN, Ivanova GN, Semena AV, Tagirova ZG, Alekseeva MN, Molochny VP, Parashar UD, Vinjé J, Maleev VV, Glass RI, Pokrovsky VI. Hospital-based surveillance of rotavirus and other viral agents of diarrhea in children and adults in Russia, 2005-2007. J. Infect. Dis. 2009; 200 Suppl 1: S228–233. |  |  |  | |  | | X | |  | |  | | X | |  | | X | |  |  | | |  |  | |  | |  | |  | |  |  | | |  | X | |  | |  | |
| Poocharoen L, Bruin CW. Campylobacter jejuni in hospitalized children with diarrhoea in Chiang Mai, Thailand. Southeast Asian J. Trop. Med. Public Health. 1986; 17(1): 53–8. |  | X |  | |  | |  | |  | |  | |  | |  | |  | |  |  | | |  |  | |  | |  | |  | |  |  | | |  |  | |  | |  | |
| Punyaratabandhu P, Vathanophas K, Varavithya W, Sangchai R, Athipanyakom S, Echeverria P, Wasi C. Childhood diarrhoea in a low-income urban community in Bangkok: incidence, clinical features, and child caretaker’s behaviours. J Diarrhoeal Dis Res 1991; 9(3): 244–9. |  |  |  | |  | | X | |  | |  | |  | |  | |  | |  |  | | |  |  | |  | |  | |  | |  |  | | |  |  | |  | |  | |
| Purohit SG, Kelkar SD, Simha V. Time series analysis of patients with rotavirus diarrhoea in Pune, India. J Diarrhoeal Dis Res. 1998 Jun;16(2):74-83. |  |  |  | |  | |  | |  | |  | |  | |  | |  | |  |  | | |  |  | |  | |  | |  | |  |  | | |  | X | |  | |  | |
| Putnam SD, Sedyaningsih ER, Listiyaningsih E, Pulungsih SP, Komalarini, Soenarto Y, Salim OC, Subekti D, Riddle MS, Burgess TH, Blair PJ. Group A rotavirus-associated diarrhea in children seeking treatment in Indonesia. J. Clin. Virol. 2007; 40(4): 289–94. |  |  |  | |  | | X | |  | |  | | X | |  | | X | |  |  | | |  |  | |  | |  | |  | |  |  | | |  |  | |  | |  | |
| Qadri F, Saha A, Ahmed T, Al Tarique A, Begum YA, Svennerholm A-M. Disease burden due to enterotoxigenic Escherichia coli in the first 2 years of life in an urban community in Bangladesh. Infect. Immun. 2007; 75(8): 3961–8. |  | X | X | |  | | X | |  | |  | | X | |  | | X | |  |  | | |  |  | |  | |  | |  | |  |  | | |  |  | |  | |  | |
| Qazi R, Sultana S, Sundar S, Warraich H, un-Nisa T, Rais A, Zaidi AK. Population-based surveillance for severe rotavirus gastroenteritis in children in Karachi, Pakistan. Vaccine. 2009 Nov 20;27 Suppl 5:F25-30. |  |  |  | |  | |  | |  | |  | |  | |  | |  | |  |  | | |  |  | |  | |  | |  | |  |  | | |  | X | |  | |  | |
| Qiao H, Nilsson M, Abreu ER, Hedlund KO, Johansen K, Zaori G, Svensson L. Viral diarrhea in children in Beijing, China. J Med Virol. 1999 Apr;57(4):390-6. |  |  |  | |  | |  | |  | |  | |  | |  | |  | | X | X | | | X |  | |  | |  | |  | |  |  | | |  | X | |  | |  | |
| Rahman M, Hassan Z, Nahar Z, Faruque AS, Van Ranst M, Rahman SR, Azim T. Molecular detection of noroviruses in hospitalized patients in Bangladesh. Eur J Clin Microbiol Infect Dis. 2010 Aug;29(8):937-45. |  |  |  | |  | |  | |  | |  | |  | |  | |  | |  |  | | |  |  | |  | |  | |  | |  |  | | |  |  | |  | |  | |
| Rahman M, Shahid NS, Rahman H, Sack DA, Rahman N, Hossain S. Cryptosporidiosis: a cause of diarrhea in Bangladesh. Am. J. Trop. Med. Hyg. 1990; 42(2): 127–30. |  |  |  | | X | |  | |  | |  | |  | |  | |  | |  |  | | |  |  | |  | |  | |  | |  |  | | |  |  | |  | |  | |
| Ramirez S, Giammanco GM, De Grazia S, Colomba C, Martella V, Arista S. Emerging GII.4 norovirus variants affect children with diarrhea in Palermo, Italy in 2006. J Med Virol. 2009 Jan;81(1):139-45. |  |  |  | |  | |  | |  | |  | |  | |  | |  | |  |  | | |  |  | |  | |  | |  | |  |  | | |  |  | |  | |  | |
| Ramiro Cruz J, Cano F, Bartlett AV, Méndez H. Infection, diarrhea, and dysentery caused by Shigella species and Campylobacter jejuni among Guatemalan rural children. Pediatr. Infect. Dis. J. 1994; 13(3): 216–23. |  | X |  | |  | |  | |  | |  | |  | |  | |  | |  |  | | |  | X | |  | |  | |  | |  |  | | |  |  | |  | | X | |
| Rao MR, Abu-Elyazeed R, Savarino SJ, Naficy AB, Wierzba TF, Abdel-Messih I, Shaheen H, Frenck RW Jr, Svennerholm A-M, Clemens JD. High disease burden of diarrhea due to enterotoxigenic Escherichia coli among rural Egyptian infants and young children. J. Clin. Microbiol. 2003; 41(10): 4862–4. |  | X |  | |  | |  | |  | |  | |  | |  | |  | |  |  | | |  | X | |  | |  | |  | |  |  | | |  |  | |  | |  | |
| Rimoldi SG, Stefani F, Pagani C, Chenal LL, Zanchetta N, Di Bartolo I, Lombardi A, Ruggeri FM, Di Lillo D, Zuccotti GV, Gismondo MR. Epidemiological and clinical characteristics of pediatric gastroenteritis associated with new viral agents.Arch Virol. 2011 Sep;156(9):1583-9. |  |  |  | |  | |  | |  | |  | |  | |  | |  | | X |  | | | X |  | |  | |  | |  | |  |  | | |  | X | |  | |  | |
| Rosa E Silva ML, Pires De Carvalho I, Gouvea V. 1998-1999 rotavirus seasons in Juiz de Fora, Minas Gerais, Brazil: detection of an unusual G3P[4] epidemic strain. J. Clin. Microbiol. 2002; 40(8): 2837–42. |  |  |  | |  | | X | |  | |  | | X | |  | | X | |  |  | | |  |  | |  | |  | |  | |  |  | | |  |  | |  | |  | |
| Sáfadi MA, Berezin EN, Munford V, Almeida FJ, de Moraes JC, Pinheiro CF, Racz ML. Hospital-based surveillance to evaluate the impact of rotavirus vaccination in São Paulo, Brazil.Pediatr Infect Dis J. 2010 Nov;29(11):1019-22. |  |  |  | |  | |  | |  | |  | |  | |  | |  | |  |  | | |  |  | |  | |  | |  | |  |  | | |  |  | |  | |  | |
| Sakamoto T, Negishi H, Wang QH, Akihara S, Kim B, Nishimura S, Kaneshi K, Nakaya S, Ueda Y, Sugita K, Motohiro T, Nishimura T, Ushijima H. Molecular epidemiology of astroviruses in Japan from 1995 to 1998 by reverse transcription-polymerase chain reaction with serotype-specific primers (1 to 8). J Med Virol. 2000 Jul;61(3):326-31. |  |  |  | |  | |  | |  | |  | |  | |  | |  | | X | X | | | X |  | |  | |  | |  | |  |  | | |  | X | |  | |  | |
| Sakvarelidze LA, Zangaladze ED. Etiological significance of rotaviruses in acute intestinal diseases in children. Vopr. Virusol. 1986; 31(6): 695–7. |  |  |  | |  | | X | |  | |  | | X | |  | | X | |  |  | | |  |  | |  | |  | |  | |  |  | | |  |  | |  | |  | |
| Salinas B, González G, González R, Escalona M, Materán M, Schael IP. Epidemiologic and clinical characteristics of rotavirus disease during five years of surveillance in Venezuela. Pediatr. Infect. Dis. J. 2004; 23(10 Suppl): S161–167. |  |  |  | |  | | X | |  | |  | | X | |  | | X | |  |  | | |  |  | |  | |  | |  | |  |  | | |  |  | |  | |  | |
| Samonis G, Maraki S, Christidou A, Georgiladakis A, Tselentis Y. Bacterial pathogens associated with diarrhoea on the island of Crete. Eur. J. Epidemiol. 1997; 13(7): 831–6. |  | X |  | |  | |  | | X | |  | |  | | X | |  | |  |  | | |  |  | |  | |  | |  | |  |  | | |  |  | |  | |  | |
| Santos N, Hoshino Y. Global distribution of rotavirus serotypes/genotypes and its implication for the development and implementation of an effective rotavirus vaccine. Rev Med Virol. 2005 Jan-Feb;15(1):29-56. |  |  |  | |  | |  | |  | |  | |  | |  | |  | |  |  | | |  |  | |  | |  | |  | |  |  | | |  | X | |  | |  | |
| Santos N, Volotão EM, Soares CC, Albuquerque MC, Da Silva FM, De Carvalho TR, Pereira CF, Chizhikov V, Hoshino Y. Rotavirus strains bearing genotype G9 or P[9] recovered from Brazilian children with diarrhea from 1997 to 1999. J. Clin. Microbiol. 2001; 39(3): 1157–60. |  |  |  | |  | |  | |  | |  | | X | |  | | X | |  |  | | |  |  | |  | |  | |  | |  |  | | |  |  | |  | |  | |
| Santos N, Volotão EM, Soares CC, Campos GS, Sardi SI, Hoshino Y. Predominance of rotavirus genotype G9 during the 1999, 2000, and 2002 seasons among hospitalized children in the city of Salvador, Bahia, Brazil: implications for future vaccine strategies. J. Clin. Microbiol. 2005; 43(8): 4064–9. |  |  |  | |  | | X | |  | |  | | X | |  | | X | |  |  | | |  |  | |  | |  | |  | |  |  | | |  |  | |  | |  | |
| Santosham M, Sack RB, Reid R, Black R, Croll J, Yolken R, Aurelian L, Wolff M, Chan E, Garrett S. Diarrhoeal diseases in the White Mountain Apaches: epidemiologic studies. J Diarrhoeal Dis Res 1995; 13(1): 18–28. |  | X |  | |  | | X | |  | | X | | X | | X | | X | |  |  | | |  |  | |  | |  | |  | |  |  | | |  |  | |  | |  | |
| Schorling JB, Wanke CA, Schorling SK, McAuliffe JF, De Souza MA, Guerrant RL. A prospective study of persistent diarrhea among children in an urban Brazilian slum. Patterns of occurrence and etiologic agents. Am. J. Epidemiol. 1990; 132(1): 144–56. | X |  |  | |  | | X | | X | | X | | X | |  | | X | |  |  | | |  | X | |  | | X | | X | |  | X | | | X | X | | X | | X | |
| Sdiri-Loulizi K, Ambert-Balay K, Gharbi-Khelifi H, Hassine M, Chouchane S, Sakly N, Neji-Guediche M, Pothier P, Aouni M. Molecular epidemiology and clinical characterization of group A rotavirus infections in Tunisian children with acute gastroenteritis. Can J Microbiol 2011;57:810-19. |  |  |  | |  | |  | |  | |  | |  | |  | |  | |  |  | | |  |  | |  | |  | |  | |  |  | | |  |  | |  | |  | |
| Shahid NS, Greenough WB 3rd, Samadi AR, Huq MI, Rahman N. Hand washing with soap reduces diarrhoea and spread of bacterial pathogens in a Bangladesh village. J Diarrhoeal Dis Res 1996; 14(2): 85–9. |  | X |  | |  | | X | |  | | X | | X | |  | | X | |  |  | | |  |  | |  | |  | |  | |  |  | | |  |  | |  | |  | |
| Sherchand JB, Nakagomi O, Dove W, Nakagomi T, Yokoo M, Pandey BD, Cuevas LE, Hart CA, Cunliffe NA. Molecular epidemiology of rotavirus diarrhea among children aged <5 years in nepal: predominance of emergent G12 strains during 2 years. J. Infect. Dis. 2009; 200 Suppl 1: S182–187. |  |  |  | |  | | X | |  | |  | | X | |  | | X | |  |  | | |  |  | |  | |  | |  | |  |  | | |  |  | |  | |  | |
| Siddique AK, Ahmed S, Iqbal A, Sobhan A, Poddar G, Azim T, Sack DA, Rahman M, Sack RB. Epidemiology of rotavirus and cholera in children aged less than five years in rural Bangladesh. J Health Popul Nutr. 2011 Feb;29(1):1-8. |  |  |  | |  | |  | |  | |  | |  | |  | |  | |  |  | | |  |  | |  | |  | |  | |  |  | | |  | X | |  | |  | |
| Simovan’ian EN, Loverdo RG, Zarubinski? VI, Kolpakov SA, Avrorov VP. Clinical course, diagnosis and treatment of Rotavirus infection in young children. Pediatriia 1989; 2: 47–51. |  |  |  | |  | | X | |  | |  | | X | |  | | X | |  |  | | |  |  | |  | |  | |  | |  |  | | |  |  | |  | |  | |
| Soares CC, Volotão EM, Albuquerque MCM, Da Silva FM, De Carvalho TRB, Nozawa CM, Linhares RE, Santos N. Prevalence of enteric adenoviruses among children with diarrhea in four Brazilian cities. J. Clin. Virol. 2002; 23(3): 171–7. |  |  |  | |  | | X | |  | |  | | X | |  | | X | |  |  | | |  |  | |  | |  | |  | |  |  | | |  |  | |  | |  | |
| Soenarto Y, Aman AT, Bakri A, Waluya H, Firmansyah A, Kadim M, Martiza I, Prasetyo D, Mulyani NS, Widowati T, Soetjiningsih, Karyana IPG, Sukardi W, Bresee J, Widdowson M-A. Burden of severe rotavirus diarrhea in indonesia. J. Infect. Dis. 2009; 200 SUPPL 1: S188–194. |  |  |  | |  | | X | |  | |  | | X | |  | | X | |  |  | | |  |  | |  | |  | |  | |  |  | | |  | X | |  | |  | |
| Sowmyanarayanan TV, Natarajan SK, Ramachandran A, Sarkar R, Moses PD, Simon A, Agarwal I, Christopher S, Kang G.Nitric oxide production in acute gastroenteritis in Indian children. Trans R Soc Trop Med Hyg. 2009 Aug;103(8):849-51. |  |  |  | |  | |  | |  | |  | |  | |  | |  | |  |  | | |  |  | |  | |  | |  | |  |  | | |  |  | |  | |  | |
| Spencer HC, Wells JG, Gary GW, Sondy J, Puhr ND, Feldman RA. Diarrhea in a non-hospitalized rural Salvadoran population: the role of enterotoxigenic Escherichia coli and rotavirus. Am. J. Trop. Med. Hyg. 1980; 29(2): 246–53. |  |  |  | |  | |  | |  | | X | |  | |  | |  | |  |  | | |  |  | |  | |  | |  | |  |  | | |  |  | |  | |  | |
| Spynu KI, Grushko TP, Vutkarev VP, Kostritsa SS. The results of a study of the contamination of bodies of water by rotaviruses against a background of gastroenteritis morbidity. Vopr. Virusol. 1991; 36(5): 423–6. |  |  |  | |  | | X | |  | |  | | X | |  | | X | |  |  | | |  |  | |  | |  | |  | |  |  | | |  |  | |  | |  | |
| Stewien KE, Mós EN, Yanaguita RM, Jerez JA, Durigon EL, Hársi CM, Tanaka H, Moraes RM, Silva LA, Santos MA, et al. Viral, bacterial and parasitic pathogens associated with severe diarrhoea in the city of Sao Paulo, Brazil. J Diarrhoeal Dis Res. 1993 Sep;11(3):148-52. |  |  |  | |  | |  | |  | |  | |  | |  | |  | | X | X | | |  | X | |  | | X | | X | |  |  | | | X | X | | X | | X | |
| Subekti D, Lesmana M, Komalarini S, Tjaniadi P, Burr D, Pazzaglia G. Enterotoxigenic Escherichia coli and other causes of infectious pediatric diarrheas in Jakarta, Indonesia. Southeast Asian J Trop Med Public Health. 1993 Sep;24(3):420-4. |  |  |  | |  | |  | |  | |  | |  | |  | |  | |  |  | | |  |  | |  | |  | |  | |  | X | | |  |  | |  | |  | |
| Subekti DS, Lesmana M, Tjaniadi P, Machpud N, Sriwati, Sukarma, Daniel JC, Alexander WK, Campbell JR, Corwin AL, Beecham HJ 3rd, Simanjuntak C, Oyofo BA. Prevalence of enterotoxigenic Escherichia coli (ETEC) in hospitalized acute diarrhea patients in Denpasar, Bali, Indonesia. Diagn Microbiol Infect Dis. 2003 Oct;47(2):399-405. |  |  |  | |  | |  | |  | |  | |  | |  | |  | |  |  | | |  |  | |  | |  | |  | |  | X | | |  |  | |  | |  | |
| Superti F, Diamanti E, Giovannangeli S, Dobi V, Xhelili L, Donelli G. Electropherotypes of rotavirus strains causing gastroenteritis in infants and young children in Tirana, Albania, from 1988 to 1991. Acta Virol. 1995 Dec;39(5-6):257-61. |  |  |  | |  | |  | |  | |  | |  | |  | |  | |  |  | | |  |  | |  | |  | |  | |  |  | | |  | X | |  | |  | |
| Suwatano O. Acute diarrhea in under five-year-old children admitted to King Mongkut Prachomklao Hospital, Phetchaburi province. J Med Assoc Thai. 1997 Jan;80(1):26-33. |  |  |  | |  | |  | |  | |  | |  | |  | |  | |  |  | | |  | X | | X | | X | | X | |  |  | | | X | X | | X | | X | |
| Tamendarova NC, Kumisbaeva ZN, Abenova UA. Use of the passive hemagglutination reaction for diagnosing rotavirus infections. Vopr. Virusol. 1989; 34(4): 501–3. |  |  |  | |  | | X | |  | |  | | X | |  | | X | |  |  | | |  |  | |  | |  | |  | |  |  | | |  |  | |  | |  | |
| Tanaka G, Faruque ASG, Luby SP, Malek MA, Glass RI, Parashar UD. Deaths from rotavirus disease in Bangladeshi children: estimates from hospital-based surveillance. Pediatr. Infect. Dis. J. 2007; 26(11): 1014–8. |  |  | X | |  | | X | |  | |  | | X | | X | | X | |  |  | | |  |  | |  | |  | |  | |  |  | | |  |  | |  | |  | |
| Tayeb HT, Balkhy HH, Aljuhani SM, Elbanyan E, Alalola S, Alshaalan M. Increased prevalence of rotavirus among children associated gastroenteritis in Riyadh Saudi Arabia Virol J. 2011 Dec 18;8:548.. |  |  |  | |  | |  | |  | |  | |  | |  | |  | |  |  | | |  |  | |  | |  | |  | |  |  | | |  | X | |  | |  | |
| Torres ME, Pírez MC, Schelotto F, Varela G, Parodi V, Allende F, Falconi E, Dell'Acqua L, Gaione P, Méndez MV, Ferrari AM, Montano A, Zanetta E, Acuña AM, Chiparelli H, Ingold E. Etiology of children's diarrhea in Montevideo, Uruguay: associated pathogens and unusual isolates. J Clin Microbiol. 2001 Jun;39(6):2134-9. |  |  |  | |  | |  | |  | |  | |  | |  | |  | |  |  | | |  | X | | X | | X | | X | |  | X | | | X | X | | X | | X | |
| Tran A, Talmud D, Lejeune B, Jovenin N, Renois F, Payan C, Leveque N, Andreoletti L. Prevalence of rotavirus, adenovirus, norovirus, and astrovirus infections and coinfections among hospitalized children in northern France. J Clin Microbiol. 2010 May;48(5):1943-6. |  |  |  | |  | |  | |  | |  | |  | |  | |  | |  |  | | |  |  | |  | |  | |  | |  |  | | |  |  | |  | |  | |
| Trimis G, Koutsoumbari I, Kottaridi C, Palaiologou N, Assimakopoulou E, Spathis A, Lebessi E, Konstantopoulos A, Kafetzis D, Karakitsos P, Papaevangelou V.Hospital-based surveillance of rotavirus gastroenteritis in the era of limited vaccine uptake through the private sector. Vaccine. 2011 Oct 6;29(43):7292-5. |  |  |  | |  | |  | |  | |  | |  | |  | |  | |  |  | | |  |  | |  | |  | |  | |  |  | | |  | X | |  | |  | |
| Tumwine JK, Kekitiinwa A, Bakeera-Kitaka S, Ndeezi G, Downing R, Feng X, Akiyoshi DE, Tzipori S. Cryptosporidiosis and microsporidiosis in ugandan children with persistent diarrhea with and without concurrent infection with the human immunodeficiency virus. Am. J. Trop. Med. Hyg. 2005; 73(5): 921–5. |  |  |  | | X | |  | |  | |  | |  | |  | |  | |  |  | | |  |  | |  | |  | |  | |  |  | | |  |  | |  | |  | |
| Uchida R, Pandey BD, Sherchand JB, Ahmed K, Yokoo M, Nakagomi T, Cuevas LE, Cunliffe NA, Hart CA, Nakagomi O. Molecular epidemiology of rotavirus diarrhea among children and adults in Nepal: detection of G12 strains with P[6] or P[8] and a G11P[25] strain. J. Clin. Microbiol. 2006; 44(10): 3499–505. |  |  |  | |  | | X | |  | |  | | X | |  | | X | |  |  | | |  |  | |  | |  | |  | |  |  | | |  |  | |  | |  | |
| Van Damme P, Giaquinto C, Huet F, Gothefors L, Maxwell M, Van der Wielen M. Multicenter prospective study of the burden of rotavirus acute gastroenteritis in Europe, 2004-2005: the REVEAL study. J. Infect. Dis. 2007; 195 Suppl 1: S4–S16. |  |  |  | |  | | X | |  | |  | | X | |  | | X | |  |  | | |  |  | |  | |  | |  | |  |  | | |  |  | |  | |  | |
| Van Man N, Luan LT, Trach DD, Thanh NTH, Van Tu P, Long NT, Anh DD, Fischer TK, Ivanoff B, Gentsch JR, Glass RI. Epidemiological profile and burden of rotavirus diarrhea in Vietnam: 5 years of sentinel hospital surveillance, 1998-2003. J. Infect. Dis. 2005; 192 Suppl 1: S127–132. |  |  |  | |  | | X | |  | |  | | X | |  | | X | |  |  | | |  |  | |  | |  | |  | |  |  | | |  | X | |  | |  | |
| Vasil’ev BI, Semenov NV, Moskvin AA, Sukhinin VP, Sirotkin AK. The etiologic role of rotaviruses in intestinal pathology in adults and children. Vopr. Virusol. 1989; 34(1): 106–9. |  |  |  | |  | | X | |  | |  | | X | |  | | X | |  |  | | |  |  | |  | |  | |  | |  |  | | |  |  | |  | |  | |
| Verma H, Chitambar SD, Gopalkrishna V. Astrovirus associated acute gastroenteritis in western India: predominance of dual serotype strains. Infect Genet Evol. 2010 May;10(4):575-9. |  |  |  | |  | |  | |  | |  | |  | |  | |  | |  | X | | |  |  | |  | |  | |  | |  |  | | |  | X | |  | |  | |
| Verma H, Chitambar SD, Varanasi G. Identification and characterization of enteric adenoviruses in infants and children hospitalized for acute gastroenteritis. J Med Virol. 2009 Jan;81(1):60-4. |  |  |  | |  | |  | |  | |  | |  | |  | |  | | X |  | | |  |  | |  | |  | |  | |  |  | | |  | X | |  | |  | |
| Viettro A, Monteverde N, Pinchak C. Caracteristicas clinicas y etiologicas de la enfermedad diarreica aguda en ninhos menores de cinco anhos hospitalizados en el Hospital Central de las Fuerzas Armadas (2004-2006). Arch Pediatr Urug 2009;80(1):17-22 |  |  |  | |  | |  | |  | |  | |  | |  | |  | |  |  | | |  |  | |  | |  | |  | |  |  | | |  | X | | X | | X | |
| Volotão EM, Soares CC, Maranhão AG, Rocha LN, Hoshino Y, Santos N. Rotavirus surveillance in the city of Rio de Janeiro-Brazil during 2000-2004: detection of unusual strains with G8P[4] or G10P[9] specificities. J. Med. Virol. 2006; 78(2): 263–72. |  |  |  | |  | | X | |  | |  | | X | |  | | X | |  |  | | |  |  | |  | |  | |  | |  |  | | |  |  | |  | |  | |
| Vorotyntseva NV, Mazankova LN, Shekoian LA, Drondina AN, Korolev MB. Incidence and clinical characteristics of rotavirus infection in children. Pediatriia 1984; 9: 50–3. |  |  |  | |  | | X | |  | |  | | X | |  | | X | |  |  | | |  |  | |  | |  | |  | |  |  | | |  |  | |  | |  | |
| Vorotyntseva NV, Mazankova LN, Shekoian LA, Ulisko IN. The significance of rotavirus and Escherichia associations in diarrheal diseases in children. Pediatriia 1988; 8: 55–8. |  |  |  | |  | | X | |  | |  | | X | |  | | X | |  |  | | |  |  | |  | |  | |  | |  |  | | |  |  | |  | |  | |
| Watson B, Ellis M, Mandal B, Dunbar E, Whale K, Brennand J. A comparison of the clinico-pathological features with stool pathogens in patients hospitalised with the symptom of diarrhoea. J. Infect. Dis. 1986; 18(6): 553–9. |  | X |  | |  | |  | |  | |  | |  | | X | |  | |  |  | | |  |  | |  | |  | |  | |  |  | | |  |  | |  | |  | |
| Wilopo SA, Soenarto Y, Bresee JS, Tholib A, Aminah S, Cahyono A, Gentsch JR, Kilgore P, Glass RI. Rotavirus surveillance to determine disease burden and epidemiology in Java, Indonesia, August 2001 through April 2004. Vaccine. 2009 Nov 20;27 Suppl 5:F61-6. |  |  |  | |  | |  | |  | |  | |  | |  | |  | |  |  | | |  |  | |  | |  | |  | |  |  | | |  | X | |  | |  | |
| Wu FT, Liang SY, Tsao KC, Huang CG, Lin CY, Lin JS, Su CY, Eng HL, Yang JY, Chen PJ, Yang CF. Hospital-based surveillance and molecular epidemiology of rotavirus infection in Taiwan, 2005-2007. Vaccine 2009; 27 Suppl 5: F50–54. |  |  |  | |  | | X | |  | |  | | X | |  | | X | |  |  | | |  |  | |  | |  | |  | |  |  | | |  | X | |  | |  | |
| Xu J, Yang Y, Sun J, Ding Y, Su L, Fang Z, Glass RI. Molecular epidemiology of rotavirus infections among children hospitalized for acute gastroenteritis in Shanghai, China, 2001 through 2005. J. Clin. Virol. 2009; 44(1): 58–61. |  |  |  | |  | | X | |  | |  | | X | |  | | X | |  |  | | |  |  | |  | |  | |  | |  |  | | |  | X | |  | |  | |
| Xu J, Yang Y, Sun J, Ding Y. Molecular epidemiology of norovirus infection among children with acute gastroenteritis in Shanghai, China, 2001-2005. J Med Virol. 2009 Oct;81(10):1826-30. |  |  |  | |  | |  | |  | |  | |  | |  | |  | |  |  | | | X |  | |  | |  | |  | |  |  | | |  |  | |  | |  | |
| Zaman K, Yunus M, Faruque AS, El Arifeen S, Hossain I, Azim T, Rahman M, Podder G, Roy E, Luby S, Sack DA. Surveillance of rotavirus in a rural diarrhoea treatment centre in Bangladesh, 2000-2006. Vaccine. 2009 Nov 20;27 Suppl 5:F31-4. |  |  |  | |  | |  | |  | |  | |  | |  | |  | |  |  | | |  |  | |  | |  | |  | |  |  | | |  | X | |  | |  | |
| Zaman R. Campylobacter enteritis in Saudi Arabia. Epidemiol. Infect. 1992; 108(1): 51–8. |  | X |  | |  | |  | |  | |  | |  | | X | |  | |  |  | | |  |  | |  | |  | |  | |  |  | | |  |  | |  | |  | |
| Zarubinskiĭ VI, Kolpakov SA. Use of the indirect hemagglutination reaction for the diagnosis of rotavirus gastroenteritis. Vopr. Virusol. 1989; 34(2): 250–4. |  |  |  | |  | | X | |  | |  | | X | |  | | X | |  |  | | |  |  | |  | |  | |  | |  |  | | |  |  | |  | |  | |
| Zuccotti G, Meneghin F, Dilillo D, Romanò L, Bottone R, Mantegazza C, Giacchino R, Besana R, Ricciardi G, Sterpa A, Altamura N, Andreotti M, Montrasio G, Macchi L, Pavan A, Paladini S, Zanetti A, Radaelli G. Epidemiological and clinical features of rotavirus among children younger than 5 years of age hospitalized with acute gastroenteritis in Northern Italy. BMC Infect Dis. 2010 Jul 22;10:218. doi: 10.1186/1471-2334-10-218. |  |  |  | |  | |  | |  | |  | |  | |  | |  | |  |  | | |  |  | |  | |  | |  | |  |  | | |  | X | |  | |  | |
| Zulfiqar A. Bhutta. Personal communication to Rafael Lozano. ; : . |  |  |  | |  | |  | |  | |  | | X | |  | | X | |  |  | | |  |  | |  | |  | |  | |  |  | | |  |  | |  | |  | |
| Zvizdić S, Telalbasić S, Beslagić E, Cavaljuga S, Maglajlić J, Zvizdić A, Hamzić S. Clinical characteristics of rotaviruses disease. Bosn J Basic Med Sci 2004; 4(2): 22–4. |  |  |  | |  | | X | |  | |  | | X | |  | | X | |  |  | | |  |  | |  | |  | |  | |  |  | | |  |  | |  | |  | |
