# Supplementary material for: Deconstructing the differences: a comparison of GBD 2010 and CHERG’s approach to estimating the mortality burden of diarrhea, pneumonia, and their etiologies
Source: BMC Infect Dis. 2015 Jan 16;15:16. doi: 10.1186/s12879-014-0728-4 (PMC4305232; doi:10.1186/s12879-014-0728-4)
Supplement: Supplementary file 4 — Supplementary Materials: Analytical models for estimating cause-specific mortality. [file 12879_2014_728_MOESM4_ESM.docx]

**Additional File 4: Supplementary Materials:**

## Analytical models for estimating cause-specific mortality

*GBD 2010*

The GBD 2010 modeling process used the following steps for pneumonia and diarrhea mortality estimation: 1) data identification, 2) data processing, 3) Cause of Death Ensemble models (CODEm) for modeling acute respiratory infections (ARI) and diarrheal deaths, 4) proportional model for dividing ARI into lower respiratory infections (LRI) and upper respiratory infections (URI) deaths, 5) DisMod-MR for estimating deaths due to LRI and diarrhea etiologies, and 6) CoDCorrect for scaling cause-specific mortality fractions to sum to one (Figure 4).

The first step of their analytical approach for estimating all causes of death for all age groups, including under-five (U5) mortality due to ARI and diarrhea and their respective etiologies, GBD 2010 identified all vital registration (VR) data sources, verbal autopsy (VA) studies, and Demographic Health Surveillance Sites data. After identifying all possible data, including published and unpublished sources, step two involved the preprocessing of this data in three ways. First, undefined or ill-defined causes of death (“garbage” codes) were redistributed. For example, deaths coded as fever or extreme dehydration were reassigned to pneumonia, malaria, and other relevant target causes. Next, different versions of the International Classification of Disease (e.g. ICD-8 and ICD-9) were mapped to match the ICD-10 causes of death. Data were adjusted to be comparable over time. Finally, the resulting data were then assessed for outliers. IHME’s website provides data visualizations which present data pre-and-post adjustment, as well as which data were identified as outliers: <http://viz.healthmetricsandevaluation.org/cod/?geo=USA&sex=1&adjType=0&cause=A.1.1&unit=8&minMax=self&userType=external>.

In step three, GBD 2010 used CODEm to model mortality for 133 of the 235 estimated causes of death, including ARI and diarrhea. Because there are more data available for ARI overall (i.e. LRI and URI combined), GBD 2010 began the modeling process by using CODEm to estimate the mortality due to ARI.

In step four, GBD 2010 used a fixed proportional model to split ARI deaths into URI and LRI deaths using all available cause of death data that distinguishes between URI and LRI. Ensemble modeling is a statistical technique in which the predictions of a group of base models are combined to generate a composite prediction which is believed to be more accurate than the prediction of any single model [1]. CODEm uses a three-step process to build models. 1) The first step is to create a large range of possible statistical models for each cause based on published studies and plausible relationships between covariates and the specific cause of death (the component models). 2) The second step is to assess the performance, or predictive capacity, of the component models and to generate and assess the ensemble models by randomly excluding 30% of the data and generating the component models using the remaining 70% of the data. Next, CODEm assesses the performance of the component model by using half of the excluded data (i.e., 15% of the total data) to predict mortality and to build various ensemble models from the component models. The last 15% of the data are then used to assess the predictive capacity of the ensemble models and all the component models. Unique to this process, data are randomly held out from the analysis (20 times for diarrhea and 25 times for ARI) using the same pattern of missing data as is present in the original database. This allows the predictive validity test to best represent the real or observed pattern of missing data. 3) The final step is to pick the best performing model, usually an ensemble model, based on the predictive validity test described above and estimate the cause-specific mortality rates using the full dataset [2]. CODEm provides a cause fraction and total number of deaths due to ARI and diarrhea for males and females aged 0–6, 7–27, and 28–356 days, and 1–4 years, independently. When the cause fractions for each cause of death are summed within each age group, the total may exceed one.

In step five, GBD 2010 built a database using data from existing systematic reviews and additional literature searches to split LRI and diarrhea into their respective etiologies. Calculations were performed on the number of deaths due to five etiologies of LRI (influenza, pneumococcal pneumonia, *H. influenzae* type b (Hib) pneumonia, respiratory syncytial virus (RSV), and other LRI) and ten etiologies of diarrhea (rotavirus, enteropathogenic *E. coli*, enterotoxigenic *E.* *coli*, *Campylobacter*, *Cryptosporidium*, *Shigella*, Cholera, Amoebiasis, other salmonella infection (Non-typhoidal salmonella: NTS), and other diarrhea pathogens) using the DisMod-MR data model, which is a Bayesian meta-regression tool [3, 4]. DisMod-MR is a nonlinear mixed effects model capable of integrating data on disease incidence, prevalence, remission, and mortality. When applied to estimating etiologic fractions of disease, DisMod-MR uses an age-standardizing negative-binomial spline model to combine measurements of disease from heterogeneous age groups, and from different years and geographic areas [5]. Random effects of country, region, and super-region quantify the geographic variation in the etiologic fractions. Fixed effects capture study-level variation, such as sex, method of diagnosis, and setting, and country-level variation, such as country income, malnutrition prevalence, and smoking prevalence. DisMod-MR produces point estimates and uncertainty intervals for each etiologic fraction for each country, sex, and year, using an empirical Bayes method.

Step six in the analytical approach is to use CoDCorrect to adjust the cause-specific mortality fractions to sum to one. The algorithm takes into account the fact that certain causes of death were known with greater precision than others and ‘penalizes’ those causes of death. CoDCorrect was applied in a hierarchal fashion based on the GBD 2010 cause hierarchy. Level 1 causes are known with the greatest precision and Level 3 with the least precision. For the causes under study in this discussion paper, diarrhea and ARI (LRI and URI combined), and all other Level 1 causes are fit into the mortality envelope generated for all-cause mortality. As described above, a fixed proportion model is then used to distribute ARI into the Level 2 causes: LRI and URI. The LRI and diarrhea etiologies (Level 3) are then fit into the LRI and diarrhea mortality envelopes respectively. This is implemented at the draw level (1000 draws that represent uncertainty) [2].

## *CHERG*

The CHERG approach used the following steps 1) data source identification, 2) data processing, 3) use of one of three models to estimate neonatal and postneonatal mortality separately according to mortality level, disease profile, and data availability, 4) development of a disease-specific model to estimate neonatal pneumonia and tetanus, as well as measles and pertussis among older children, and 5) adjusting deaths to sum to the total U5 mortality (Figure 5) [6].

After identifying data sources and cleaning the data, country-specific proportions of neonatal and postneonatal deaths were assigned to a specific cause using one of three different methods depending on the coverage and quality of VR data and the under-five mortality rate (U5MR) in each country:

1. Use of VR data: used when a country's coverage of the VR system was high (>80%) and quality was perceived as good. Data were adjusted for coverage incompleteness if needed by reassigning illogical causes of death and then grouped according to standard ICD-10 codes.
2. Use of a vital-registration-data-based multi-cause model (VRMCM): used in countries with inadequate vital registration system and lower U5MRs (≤35 deaths per 1000 live births). CHERG used univariate meta-regression and multivariate ordinary least squares regression to identify explanatory variables (risk factors) for the log ratio of each cause with pneumonia as the reference category. CHERG applied the results of the model to calculate the proportional mortality for each of the eight etiology groupings.
3. Use of a verbal-autopsy-data-based multi-cause model (VAMCM): used in countries with poor VR coverage and high U5MRs (>35 per 1000 live births). CHERG used ordinary least squares to determine inclusion of risk factor data in a multinomial logistic regression (with pneumonia as the reference category) [6]. CHERG applied country- and year-specific data points for each covariate to calculate the proportional mortality for each of the eight etiology groupings.

In an update of the CHERG approach by Liu et al., neonatal pneumonia deaths were estimated separately from the VAMCM and VRMCM models [6]. An additional 36 studies where deaths due to neonatal pneumonia were reported were used to estimate the proportion of neonatal deaths attributed to pneumonia using a logistic regression model.

Post-hoc adjustments were made to the country-level cause of death (COD) estimates for pneumonia, malaria, and meningitis to account for health intervention coverage and effectiveness. The deaths ‘saved’ by the intervention were reallocated to the other COD categories by relative importance. Pneumonia and meningitis deaths were adjusted for the use and effectiveness of three doses of Hib vaccine. Malaria deaths were adjusted for the use and effectiveness of insecticide treated nets.

For all neonatal and postneonatal VRMCMs and VAMCMs, 10% of the data were reserved for cross-validation of the models to compare out-of-sample prediction accuracy between model versions. The best-performing model was chosen as the final model with the smallest absolute prediction error. Bootstrapping was used to construct uncertainty ranges for estimates produced by VRMCMs and VAMCMs.

CHERG’s etiology-specific mortality estimates for pneumonia (Hib and pneumococcus only) and diarrhea were calculated using separate models [7, 8]. The conceptual and statistical approach for the pneumococcus- and Hib-specific pneumonia mortality estimates was based on a previous estimation project from the WHO, which aimed to estimate country-level pneumococcal and Hib morbidity and mortality for pneumonia, meningitis and non-pneumonia/non-meningitis in the year 2000 [9, 10]. For pneumonia etiologies, the conceptual approach applied the country-specific pneumonia mortality values to the proportion of cases due to pneumococcus and Hib. The etiologic fractions for the deaths were assumed to be the same as the proportion of chest x-ray confirmed pneumonias that are due to Hib and pneumococcus. In other words, if 33% of chest x-ray confirmed pneumonias were attributed to pneumococcus, then 33% of pneumonia deaths were attributed to pneumococcus. The latter proportion was calculated using values from randomized controlled trials of pneumococcal or Hib vaccines and generating a single global point estimate through a random effects meta-analysis. CHERG assumed that 21.3% of pneumonia deaths were due to Hib and 33.3% were due to pneumococcus. These two estimates were determined through meta-analyses of vaccine efficacy studies [7, 9, 10]. This approach assumes that the underlying etiologic distribution of pneumonia in the absence of Hib and pneumococcal vaccination is the same across all geographies. Because of sparse data for estimating the fraction of pneumonia deaths from viral etiologies, point estimates were not available for RSV and influenza mortality; therefore, the remaining 46% of pneumonia deaths were not assigned to an etiology. However, Nair et al. (2010 and 2011) from CHERG have published mortality upper and lower “bounds” which will be improved due to a current data collecting exercise funded by BMGF [11, 12].

To estimate the etiologies of diarrheal diseases, CHERG conducted a systematic review of studies that analyzed stool samples to assess causation, focusing on 13 specific pathogens: rotavirus, EPEC, ETEC, *Salmonella spp.* (excluding *S. typhi*), *Shigella spp.*, *Campylobacter spp.*, *Vibrio cholerae* O1 and O139, *Giardia lamblia*, *Cryptosporidium spp.*, *Entamoeba histolytica*, human Caliciviruses (genogroup I and II norovirus and sapovirus), astrovirus, and enteric adenovirus [8]. CHERG also used 2011 World Health Organization (WHO) Rotavirus Surveillance Network data from countries that have not introduced the rotavirus vaccine [13]. Rather than taking a modeling approach to estimating the etiologic fraction of each pathogen, CHERG used the age-adjusted median proportion from the studies found in the systematic review. They examined the data in several ways, including comparing the median etiologic proportions of studies that examined only one pathogen with those that examined 5–13 pathogens and scaling the etiologic proportions so that they summed to one. CHERG applied both the unscaled and scaled etiologic proportions to the total estimated number of diarrheal deaths to produce global estimates of cause-specific diarrheal deaths.

**Inclusion criteria for data**

*GBD 2010*

The GBD 2010 LRI etiology models used data from existing systematic reviews conducted by Black et al. [14], O’Brien et al. [10], Watt et al. [9], and a database of published studies of ARI compiled by the WHO. GBD 2010 included all studies from these reviews after applying additional exclusion criteria: studies not general population-representative (e.g., studies in refugee populations); studies that did not provide data on the prevalence, incidence or mortality due to LRI; studies that provided no numerator and denominator counts; studies conducted before 1980; and studies with a denominator less than 50. An additional systematic search was conducted using the same inclusion and exclusion rules for studies published between 2006–2011, which identified an additional 54 studies for analysis [2]. Ten vaccine efficacy studies were also used in the GBD 2010 LRI etiology models. Appendix 2 lists all studies used to model pneumonia etiologies by both GBD 2010 and CHERG.

The GBD 2010 diarrhea etiology models used the following exclusion criteria for studies: case-control studies that did not include data for controls, studies that provided no measurements (only estimates), studies that did not include the proportion of diarrhea cases with positive laboratory tests for at least one pathogen, studies that were conducted for fewer than 12 months (which did not account for potential effects of seasonality), studies that only reported data for HIV-positive patients, studies that assessed travelers, and studies of populations experiencing outbreaks of diarrhea due to a pathogen. GBD 2010 also used unpublished data from a large cohort study in Pakistan. In total they included 189 studies from 1975–2010 with rotavirus providing the largest number of studies at 126 [2].

**Model Covariates:**

*Pneumonia Covariates*

To estimate the number of deaths due to ARI, GDB 2010 used the following covariates in the CODEm model: Hib3 vaccine coverage, health system access, malnutrition (proportion <2 SD weight for age), indoor air pollution, outdoor air pollution, DTP3 coverage (proportion), rainfall (quintiles 4-5), proportion of population with access to improved sanitation, proportion of population with access to improved water, average years of education per capita, log lag-distributed income (USD per capita), and proportion of population in areas over 1,000 people per square kilometer. GBD 2010 used the following covariates to estimate LRI mortality for U5 children: healthcare access, Hib3 vaccine coverage, access to sanitation and clean water, malnutrition, education, population density, and the log of lag-distributed income.

For the LRI etiology models, Hib3 vaccine coverage was included as a covariate to estimate the proportion of LRI due to Hib, and health system access as a covariate for all of the five etiologies. GBD 2010 included as a study-level covariate whether the study investigated the etiology of LRI in inpatients or in an outpatient/community-based setting. Estimates for inpatients were assumed to be representative of severe cases of LRI, whereas estimates for outpatient or community-based settings were assumed to be representative of all cases of LRI. For the efficacy study data, they assumed that clinical pneumonia was analogous to all cases of LRI and chest radiography-positive pneumonia to be analogous to severe cases of LRI. Proportions corresponding to clinical and chest radiography-positive pneumonia were therefore coded as outpatient and inpatient, respectively. Inpatient studies were set as the reference category in the model so that the resultant proportions estimated would represent severe cases of LRI.

Because pneumonia was used as the reference category for CHERG’s VAMCM model, no specific covariates were used to estimate postneonatal pneumonia mortality. Instead, all covariates used to estimate the other seven causes of death informed the estimates for pneumonia mortality. For neonates, the following covariates were used in the VAMCM model for severe infection: neonatal mortality rate, DPT3 vaccination coverage, Quad-DPT coverage, and period (early, late, or overall). In the VRMCM for severe infection, early neonatal was modeled using access to antenatal care, female literacy and region, while late neonatal was modeled using female literacy, under-five mortality rate, births with skilled attendant, low birth weight, and region as covariates. The VAMCM model for neonatal pneumonia deaths used female literacy, low birth weight, birth with skilled attendant, and tetanus toxoid vaccine coverage at birth as covariates. In the VRMCM model, female literacy, neonatal morality rate, births will skilled attendant, and region were used as covariates for pneumonia deaths in early neonates, while access to antenatal care, region, GFR, and births with skilled attendant were used for late neonatal pneumonia. For the pneumonia etiology model, estimates for pneumococcal pneumonia and Hib deaths were based on each country’s pneumococcal and Hib3 vaccination coverage, respectively.

*Diarrhea Covariates*

GBD 2010 estimated the number of deaths due to diarrhea using the following covariates: health care access, rotavirus vaccine coverage, access to sanitation and water, percentage of children under 2 malnourished, education, population density, and the log of lag-distributed income ($ per capita).

CHERG used the following covariates in their neonatal VAMCM model for diarrhea deaths: neonatal mortality rate, DPT vaccination coverage, Quad-DPT vaccination coverage, and period (early, late, overall). For the postneonatal VRMCM model for diarrhea deaths the following covariates were used: U5 population, coverage of the third dose of DPT3 vaccine, WHO Europe Region vs. other WHO regions, and gross national income (GNI, per capita). For the VAMCM model for postneonatal diarrhea deaths year of study, percent urban population, U5 mortality rate, and oral rehydration therapy coverage were used as covariates.

**References:**

1. Foreman KJ, Lozano R, Lopez AD, Murray CJ: **Modeling causes of death: an integrated approach using CODEm.** *Popul Health Metr* 2012, **10**:1.

2. Lozano R, Naghavi M, Foreman K, Lim S, Shibuya K, Aboyans V, Abraham J, Adair T, Aggarwal R, Ahn SY, Alvarado M, Anderson HR, Anderson LM, Andrews KG, Atkinson C, Baddour LM, Barker-Collo S, Bartels DH, Bell ML, Benjamin EJ, Bennett D, Bhalla K, Bikbov B, Bin Abdulhak A, Birbeck G, Blyth F, Bolliger I, Boufous S, Bucello C, Burch M, et al.: **Global and regional mortality from 235 causes of death for 20 age groups in 1990 and 2010: a systematic analysis for the Global Burden of Disease Study 2010.** *Lancet* 2012, **380**:2095–128.

3. Vos T, Flaxman AD, Naghavi M, Lozano R, Michaud C, Ezzati M, Shibuya K, Salomon J a, Abdalla S, Aboyans V, Abraham J, Ackerman I, Aggarwal R, Ahn SY, Ali MK, Alvarado M, Anderson HR, Anderson LM, Andrews KG, Atkinson C, Baddour LM, Bahalim AN, Barker-Collo S, Barrero LH, Bartels DH, Basáñez M-G, Baxter A, Bell ML, Benjamin EJ, Bennett D, et al.: **Years lived with disability (YLDs) for 1160 sequelae of 289 diseases and injuries 1990–2010: a systematic analysis for the Global Burden of Disease Study 2010**. *Lancet* 2012, **380**:2163–2196.

4. Ferrari AJ, Charlson FJ, Norman RE, Flaxman AD, Patten SB, Vos T, Whiteford H a.: **The Epidemiological Modelling of Major Depressive Disorder: Application for the Global Burden of Disease Study 2010**. *PLoS One* 2013, **8**:e69637.

5. Barendregt JJ, Van Oortmarssen GJ, Vos T, Murray CJ: **A generic model for the assessment of disease epidemiology: the computational basis of DisMod II.** *Popul Health Metr* 2003, **1**:4.

6. Liu L, Johnson HL, Cousens S, Perin J, Scott S, Lawn JE, Rudan I, Campbell H, Cibulskis R, Li M, Mathers C, Black RE: **Global, regional, and national causes of child mortality: an updated systematic analysis for 2010 with time trends since 2000.** *Lancet* 2012, **379**:2151–61.

7. Rudan I, O’Brien KL, Nair H, Liu L, Theodoratou E, Qazi S, Lukšić I, Fischer Walker CL, Black RE, Campbell H: **Epidemiology and etiology of childhood pneumonia in 2010: estimates of incidence, severe morbidity, mortality, underlying risk factors and causative pathogens for 192 countries.** *J Glob Health* 2013, **3**:10401.

8. Lanata CF, Fischer-Walker CL, Olascoaga AC, Torres CX, Aryee MJ, Black RE: **Global causes of diarrheal disease mortality in children <5 years of age: a systematic review.** *PLoS One* 2013, **8**:e72788.

9. Watt JP, Wolfson LJ, O’Brien KL, Henkle E, Deloria-Knoll M, McCall N, Lee E, Levine OS, Hajjeh R, Mulholland K, Cherian T: **Burden of disease caused by Haemophilus influenzae type b in children younger than 5 years: global estimates.** *Lancet* 2009, **374**:903–11.

10. O’Brien KL, Wolfson LJ, Watt JP, Henkle E, Deloria-Knoll M, McCall N, Lee E, Mulholland K, Levine OS, Cherian T: **Burden of disease caused by Streptococcus pneumoniae in children younger than 5 years: global estimates.** *Lancet* 2009, **374**:893–902.

11. Nair H, Gessner BD, Nokes DJ, Theodoratou E, Rudan I, Madhi S a, Simões EA, Campbell H: **Childhood mortality due to respiratory syncytial virus – Authors’ reply**. *Lancet* 2010, **376**:872–873.

12. Nair H, Brooks WA, Katz M, Roca A, Berkley J a, Madhi S a, Simmerman JM, Gordon A, Sato M, Howie S, Krishnan A, Ope M, Lindblade K a, Carosone-Link P, Lucero M, Ochieng W, Kamimoto L, Dueger E, Bhat N, Vong S, Theodoratou E, Chittaganpitch M, Chimah O, Balmaseda A, Buchy P, Harris E, Evans V, Katayose M, Gaur B, O’Callaghan-Gordo C, et al.: **Global burden of respiratory infections due to seasonal influenza in young children: a systematic review and meta-analysis.** *Lancet* 2011, **378**:1917–30.

13. World Health Organization: **Rotavirus surveillance worldwide-2009**. *Wkly Epidemiol Rec* 2011:173–176.

14. Black RE, Cousens S, Johnson HL, Lawn JE, Rudan I, Bassani DG, Jha P, Campbell H, Walker CF, Cibulskis R, Eisele T, Liu L, Mathers C: **Global, regional, and national causes of child mortality in 2008: a systematic analysis.** *Lancet* 2010, **375**:1969–87.
